# Sequential FOLFIRI.3 + Gemcitabine Improves Health-Related Quality of Life Deterioration-Free Survival of Patients with Metastatic Pancreatic Adenocarcinoma: A Randomized Phase II Trial

**DOI:** 10.1371/journal.pone.0125350

**Published:** 2015-05-26

**Authors:** Amélie Anota, Guillaume Mouillet, Isabelle Trouilloud, Anne-Claire Dupont-Gossart, Pascal Artru, Thierry Lecomte, Aziz Zaanan, Mélanie Gauthier, Francine Fein, Olivier Dubreuil, Sophie Paget-Bailly, Julien Taieb, Franck Bonnetain

**Affiliations:** 1 National Quality of Life in Oncology Platform, Besançon, France; 2 Methodological and Quality of Life in Oncology Unit (EA 3181), University Hospital of Besançon, Besançon, France; 3 Department of Gastroenterology and Digestive Oncology, Georges Pompidou European Hospital, University of Paris Descartes, Paris, France; 4 Department of Gastroenterology, University Hospital of Besançon, Besançon, France; 5 Hepato-Gastro-Enterology and Digestive Oncology Department, Hospital Jean Mermoz, Lyon, France; 6 Department of Gastroenterology and Digestive Oncology, University Hospital of Tours- Trousseau Hospital, Chambray-Les-Tours, France; 7 Biostatistics and Quality of Life Unit, Centre Georges François Leclerc, Dijon, France; Weill Cornell Medical College Qatar, QATAR

## Abstract

**Background:**

A randomized multicenter phase II trial was conducted to assess the sequential treatment strategy using FOLFIRI.3 and gemcitabine alternately (Arm 2) compared to gemcitabine alone (Arm 1) in patients with metastatic non pre-treated pancreatic adenocarcinoma. The primary endpoint was the progression-free survival (PFS) rate at 6 months. It concludes that the sequential treatment strategy appears to be feasible and effective with a PFS rate of 43.5% in Arm 2 at 6 months (26.1% in Arm 1). This paper reports the results of the longitudinal analysis of the health-related quality of life (HRQoL) as a secondary endpoint of this study.

**Methods:**

HRQoL was evaluated using the EORTC QLQ-C30 at baseline and every two months until the end of the study or death. HRQoL deterioration-free survival (QFS) was defined as the time from randomization to a first significant deterioration as compared to the baseline score with no further significant improvement, or death. A propensity score was estimated comparing characteristics of partial and complete responders. Analyses were repeated with inverse probability weighting method using the propensity score. Multivariate Cox regression analyses were performed to identify independent factors influencing QFS.

**Results:**

98 patients were included between 2007 and 2011. Adjusting on the propensity score, patients of Arm 2 presented a longer QFS of Global Health Status (Hazard Ratio: 0.52 [0.31-0.85]), emotional functioning (0.35 [0.21–0.59]) and pain (0.50 [0.31 – 0.81]) than those of Arm 1.

**Conclusion:**

Patients of Arm 2 presented a better HRQoL with a longer QFS than those of Arm 1. Moreover, the propensity score method allows to take into account the missing data depending on patients’ characteristics.

**Trial registration information:**

Eudract N° 2006-005703-34. (Name of the Trial: FIRGEM).

## Introduction

The results of a phase II trial concerning untreated patient with metastatic Pancreatic Cancer (mPC) have shown that sequential treatment using FOLFIRI.3 and gemcitabine was effective and safe [1].

In first line treatment, FOLFIRINOX protocol and the association of nab-paclitaxel + gemcitabine improve overall survival (OS) [2,3] and represent a new therapeutic option in first line. However, the less favorable toxicity profiles of these new strategies could limit this option to younger patients with a good Performance Status (0 or 1) [4]. A sequential association of chemotherapy protocol without cross-resistance may increase anti-tumor effects and limit toxicities, preserving patient’s Health-related Quality of Life (HRQoL).

Prognosis of patients with mPC remains extremely poor. In consequence, HRQoL is a major subject of concern for these patients who are often painful and symptomatic at the time of diagnosis. Moreover, HRQoL appears to be an independent prognostic factor for OS alongside classical clinical and demographic factors [5]. In metastatic settings, the current discussion is to consider HRQoL as a co-primary endpoint along with a tumor parameter such as progression-free survival (PFS) [6,7].

However, HRQoL results remain poorly used to modify therapeutic strategies, due to the complexity of its longitudinal analysis and to a lack of standardization. Moreover, results should have the ability to translate findings into information that decision makers find understandable and compelling.

In recent years, time to event models like time until definitive HRQoL score deterioration (TUDD) have been proposed as a modality of longitudinal HRQoL analysis in oncology, especially in metastatic setting [8]. The TUDD method produces clinically meaningful results for clinicians like Kaplan-Meier survival curves and hazard ratio (HR). TUDD including death as an event was defined as "HRQoL deterioration-free survival" (QFS) [9].

One other major concern of longitudinal HRQoL studies is missing data [10], specifically in advanced cancer where attrition is common [11]. Patients may dropout before the end of the study, generally due to a health status deterioration or death. In this case, missing data can bias the analysis and interpretation [10,12–14], and should be considered to ensure accuracy and robustness of the results. Several methods have been investigated to handle with missing data [15,16]. The most well-known is the pattern-mixture model [17] but it is rarely applied due to its complexity [17,18].

Then it would be interesting to develop a method to use in conjunction with QFS to handle with informative missing data. Methods using the propensity score are often used in observational studies in order to reduce the bias of the absence of randomization and to allow causal inference [19]. The propensity score is used to model the probability of receiving a treatment conditionally to the variables observed before treatment. The main methods used with propensity score are stratification, matching and inverse probability weighting (IPW) methods [20]. In survival analyses, IPW method is recommended [21]. Indeed, IPW method of the propensity score was already proposed to take into account missing data [22].

The objective of this study was to compare longitudinal HRQoL according to treatment arm using QFS in a metastatic setting and secondary to investigate the application of the IPW method based on the propensity score in conjunction with the TUDD in order to take into account missing data depending on patients’ characteristics.

## Materials and Methods

### Patients and eligibility criteria

This study was a multicenter, randomized, non-comparative, open phase II trial, conducted in French centers. Inclusion criteria were: histologically or cytologically proven mPC, no previous chemotherapy (adjuvant chemotherapy with gemcitabine was allowed if administered more than 12 months before inclusion) or radiotherapy (unless at least one measurable target lesion was present outside the irradiated area) and WHO performance status <2. Exclusion criteria were bile ducts adenocarcinoma, ampulloma and a history of another cancer. All patients were fully informed of the study and provided signed written informed consent (see S1 Informed Consent). The protocol was approved by the ethics committees (“Comité de Protection des Personnes”). This study FIRGEM was registered with EudraCT (https://eudract.ema.europa.eu/; N° 2006-005703-34) before the start date. The design of this study has been extensively described elsewhere [1]. The protocol for this trial and supporting CONSORT checklist are available as supporting information (see S1 and S2 Protocol; see S1 Checklist). List of Ethics Committees is also available in supporting information (see S1 Authorization).

Using minimization technique, patients were randomly (ratio 1:1) assigned to receive sequentially FOLFIRI.3 every 14 days during two months (four courses per cycle), followed by gemcitabine (6 courses at days 1, 8, 15, 29, 36 and 43 per cycle) (Arm 2) or gemcitabine alone (Arm 1). A deterministic minimization was employed and stratification criteria were center (10 centers), performance status (0 vs. 1) and the number of metastatic sites (one vs. more than one).

### Health-related quality of life assessment

HRQoL was evaluated using the European Organisation for Research and Treatment of Cancer (EORTC) QLQ-C30 cancer specific questionnaire [23], at inclusion and every two months until progression, limiting toxicity, patient’s refusal or death. The QLQ-C30 includes 30 items and measures five functional scales (physical, role, emotional, cognitive and social functioning), global health status (GHS), financial difficulties and eight symptom scales (fatigue, nausea and vomiting, pain, dyspnea, insomnia, appetite loss, constipation, diarrhea) [23]. These scores vary from 0 (worst) to 100 (best) for the functional dimensions and GHS, and from 0 (best) to 100 (worst) for the symptom dimensions and were generated according to the EORTC Scoring Manual [24].

### Statistical analysis

#### Sample size calculation

The primary endpoint was the 6-month PFS rate. Secondary endpoints were OS, safety/tolerability, tumor response, PFS and QFS. The trial was based on a Fleming one-step design [25]. The expected 6-month PFS rate with the sequential treatment was 45%. A PFS rate of 25% was chosen as uninteresting rate of effectiveness (H0: 6-month PFS 25% = unacceptable efficacy, H1: 6-month PFS 45% = expected efficacy). With a unilateral type I error of 5% and a type II error of 10%, it was necessary to include 46 patients in each arm, rounded to 49 to compensate for an anticipated 5% rate of loss to follow-up.

Based on Fleming decision criteria, experimental arm will be considered uninteresting if 15 or less than 15 alive patients were free of progression. It will be considered as promising if 16 or more than 16 alive patients were free of progression.

The analysis was performed on intent-to-treat principle (all randomized patients irrespective of treatment received and eligibility criteria). Analyses of primary endpoint were done on the first randomized 46 patients with available PFS data (to match with Fleming criteria decision rules) while all other analyses were done on all randomized patients. Tumour responses were defined using RECIST (version 1.1) [26] and determined by investigators.

#### Population

Randomized patients whatever eligibility criteria with at least one HRQoL score were included in the QFS analysis (modified intent to treat analysis). Pre-specified targeted HRQoL dimensions were GHS (mITT1), physical (mITT2) and emotional functioning (mITT3), fatigue (mITT4) and pain (mITT5).

Since this is a non comparative randomized phase II trial and HRQoL was an exploratory secondary endpoint, no p-value was provided while effect size was presented using hazard ratio with 95% confidence interval (CI95%). A five-point difference in HRQoL scores was considered as the Minimal Clinically Important Difference [27].

#### Descriptive analysis

Baseline variables were described using means and standard deviations for continuous variables and percentages for qualitative variables. Baseline HRQoL scores were described by treatment arm. The number of HRQoL questionnaires completed at each measurement time was reported. The Most Common Grade 3 or 4 Adverse Events occurring during the study according to the National Cancer Institute Common Terminology Criteria for Adverse Events (version 3.0) [28] were reported by treatment arm.

#### Missing data analysis

The missing data patterns were patients with at least one missing HRQoL score during the follow-up (partial responders) versus patients with all available scores until their drop-out of the study or death (complete responders). The number and percentage of patients according to the missing data profile (partial vs. complete responders) were described at each measurement time by treatment arm. The number and percentage of complete responders, partial responders and non responders (patients who did not complete any HRQoL questionnaire) were described by treatment arm and the difference between the two treatment arms was compared using Chi-square test. All baseline variables that could be associated with missing data patterns (partial vs. complete responders) were tested with an univariate logistic regression model. Variables with an univariate *P*-value ≤ 0.20 were eligible for multivariate analysis. To prevent collinearity, when two variables were significantly correlated, one variable was retained according to its clinical relevance. The final multivariate model was chosen according to the Akaike criteria and the area under the ROC curve and described with Odds-Ratio (OR) and its 95%CI. Fitted values were then extracted from the model and constituted the propensity score [29].

#### Longitudinal analysis

The QFS was defined as the time from randomization to a first deterioration with a 5-point Minimal Clinically Important Difference as compared to the baseline score with no further improvement of more than 5 points as compared to the baseline sore, or all-cause of death [8]. Patients with no baseline score were censored at baseline (Day 0). Patients with no follow-up measure were censored just after baseline (Day 1). Patients with no deterioration before their drop-out and those with a deterioration followed by a significant improvement are censored at the time of the last follow-up or the last HRQoL assessment. Each targeted dimensions of the QLQ-C30 was studied.

Based on the intention-to treat principle and according to the worst possible scenario, a sensitivity analysis was performed integrating non responders patients and considering these patients in deterioration since baseline (Day 1).

QFS curves were calculated using the Kaplan-Meier estimation method and described using median and its 95%CI. Univariate Cox analyses were done as exploratory analysis to estimate effect of treatment arm size with the HR and 95%CI. Follow-up was calculated using reverse Kaplan-Meier estimation.

To take into account missing data, analyses were repeated by assigning a weight to patients according to the IPW method of propensity score [21]. The weight equals to the inverse of the propensity score value for partial responders and to the inverse of the opposite of the propensity score value for complete responders [21].

Multivariate Cox regression model was also conducted as exploratory analysis in order to investigate parameters which seem to be associated with QFS. All variables collected at baseline were tested in univariate analysis. Some interaction effects between treatment arm and clinical variables were investigated. Variables with 1 not included in the 95%CI of the HR were eligible for multivariate analysis. The same variables were kept in multivariate analysis for unweighted and weighted QFS analyses. The variable treatment arm was forced in multivariate analysis.

All analyses were performed with R software [30].

## Results

### Study population

**Fig 1:**
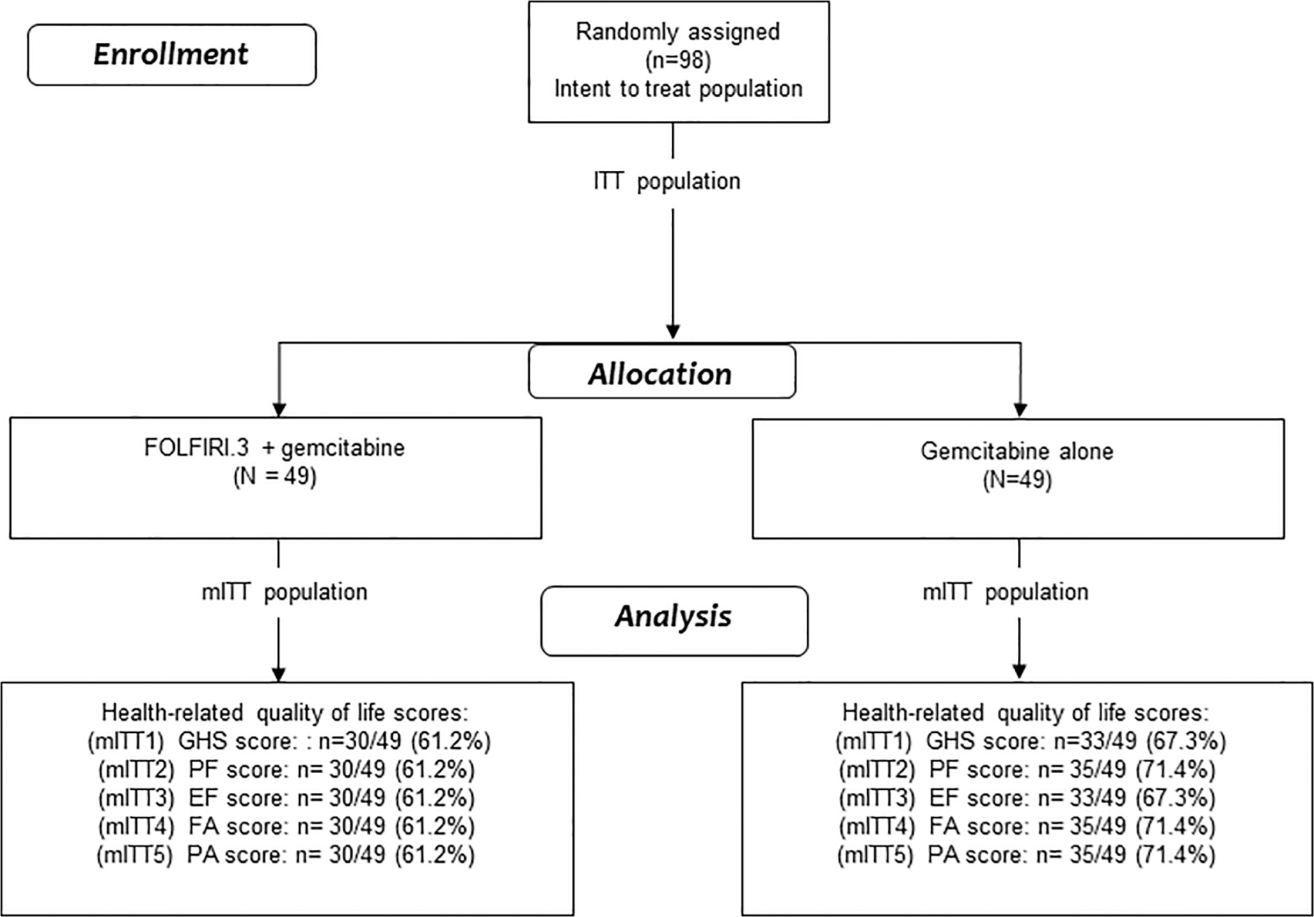
CONSORT Diagram for health-related quality of life analysis. ITT: intent to treat; mITT modified intent to treat; GHS global health status; PF: Physical functioning; EF: Emotional functioning; FA: Fatigue; PA: Pain.

Between October 2007 and May 2011, 98 patients (49 in each treatment arm) were enrolled in 10 French centers (Fig 1). Baseline characteristics of patients are summarized in Table 1. The median age was 62 years (range 38–76) and 59 patients (60.20%) were men. At baseline, 34 patients (69.4%) completed the QLQ-C30 questionnaire in Arm 1 and 30 patients (61.2%) in Arm 2. No difference of baseline HRQoL level was observed between treatment arms (S1 Table).

**Table 1:** Baseline characteristics of patients included according to treatment arm.

Variable	Response Category	Arm1 gemcitabine alone (N = 49)		Arm 2 FOLFIRI.3 + gemcitabine (N = 49)	
		*N*	%	*N*	%
**Sex**	male	28	57.1	31	63.3
	female	21	42.9	18	36.7
**WHO performance status**	0	16	32.6	16	32.7
	1	33	67.4	33	67.3
**Previous surgery**	no	40	81.6	38	77.6
	yes	9	18.4	11	22.4
**Surgery type**	curative	4	8.2	5	10.2
	palliative	5	10.2	5	10.2
	not applicable	40	81.6	38	77.6
	missing	0	0.0	1	2.0
**Number of metastatic sites**	1	35	71.4	33	67.3
	more than 1	14	28.6	16	32.7
**Previous chemotherapy**	no	40	81.6	44	89.8
	yes	3	6.1	2	4.1
	missing	6	12.2	3	6.1
**Primary tumor location**	head	29	59.2	18	36.7
	body	11	22.5	17	34.7
	tail	12	24.5	17	34.7
**Sites of metastasis**	liver	35	71.4	39	79.6
	lung	11	22.5	11	22.5
	lymph node	7	14.3	5	10.2
	peritoneal	10	20.4	16	33.7
	other	2	4.0	3	6.1
**Age (years)** ^a^		45	63 [41–76]	48	62 [38–76]
**Leukocytes (/mm3)** ^a^		49	7600 [3100–36500]	49	8300 [85–21700]
**neutrophils (/mm3)** ^a^		49	5000 [1800–32850]	48	5591.5 [2300–19530]
**Creatinine (μmol/l)** ^a^		48	71.0 [39–105]	48	70 [45–108]
**Glycaemia (mmol/l)** ^a^		29	6.2 [4.1–14]	25	5.8 [0.7–15]
**Bilirubin (μmol/l)** ^a^		48	11.6 [4–227]	45	12 [1–154]
**LDH (UI/L)** ^a^		23	271 [96–5022]	22	340.5 [133–766]
**Hemoglobin (g/dl)** ^a^		49	12.8 [7.9–16.5]	48	12.9 [9.4–16]
**Platelet (10^3/mm3)** ^a^		49	239 [94–570]	48	278.5 [111–634]
**ASAT (UI/L)** ^a^		49	26 [8–149]	46	41.5 [10–187]
**ALAT(UI/L)** ^a^		49	35 [8–155]	46	53.5 [10–348]
**Prothrombin (%)** ^a^		39	93 [26–109]	41	86 [19–122]

^a^Median [min-max] for continuous variables.

The median follow up was 32.5 months (95%CI 25.4–40.4).

The primary endpoint (6-month PFS rate), on 46 first randomized patients per arm using Fleming’s criterion was reached in Arm 2 with 20 patients alive and free of progression resulting in an observed 6-month PFS rate of 43.5% [95%CI 28.6–58.4] but not in Arm 1 with only 12 patients alive and free of progression resulting in a 6-month PFS rate of 26.1% [12.9–39.3].

Among all randomized patients, the estimated 6 months PFS rate was 25.7% [95%CI 14.4–38.6] for Arm 1 and 44.9% [30.7–58.0] for Arm 2. The objective response rate was 10.2% [1.4–19.0] for Arm 1 and 36.7% [22.7–50.7] for Arm 2. Median OS was 8.2 months [95%CI 5.3–9.2] in Arm 1 and 11 months [7.8–13.6] in Arm 2 [1].

The Most Common Grade 3 or 4 Adverse Events occurring during the study are reported in S2 Table.

### Missing data analysis

Table 2 gives the number and percentage of complete, partial and non-responders in each treatment arm.

**Table 2:** Proportion of complete, partial and non responders for HRQoL assessment in each treatment arm.

	Arm gemcitabine alone (N = 49)	Arm FOLFIRI + gemcitabine (N = 49)
Complete responders	15 (30.6)	11 (22.4)
Partial responders	15 (30.6)	25 (51.0)
Non responders	19 (38.8)	13 (26.5)

Among the 66 patients (67.3%) who had completed at least one HRQoL questionnaire during the study, 40 (60.6%) were partial responders (15 in Arm 1 (37.5%), 25 in Arm 2 (62.5%)) and 26 (39.4%) were complete responders (15 in Arm 1 (57.7%), 11 in Arm 2 (42.3%)) during the follow-up. The details of the HRQoL questionnaire completed at each follow-up measurement time according to treatment arm and missing data profile are given in Table 3.

**Table 3:** Completion of HRQoL questionnaire at each follow-up measurement time according to treatment arm and missing data profile.

		Complete responders			Partial responders	
	Arm 1	Arm 2	Total	Arm 1	Arm 2	Total
	N (%)	N (%)	N (%)	N (%)	N (%)	N (%)
cycle 1	14 (44.1)	15 (50.0)	29 (46.0)	19 (55.9)	15 (50.0)	34 (54.0)
cycle 2	13 (46.4)	7 (35.0)	20 (41.2)	15 (53.6)	13 (65.0)	28 (58.3)
cycle 3	7 (35.0)	1 (10.0)	8 (26.7)	13 (65.0)	9 (90.0)	22 (73.3)
cycle 4	4 (28.6)	1 (50.0)	5 (31.3)	10 (71.4)	1 (50.0)	11 (68.8)
cycle 5	3 (50.0)	1 (50.0)	4 (50.0)	3 (50.0)	1 (50.0)	4 (50.0)
cycle 6	1 (33.3)	0 (0.0)	1 (25.0)	2 (66.7)	1 (100.0)	3 (75.0)
cycle 7	1 (50.0)	0 (0.0)	1 (50.0)	1 (50.0)	0 (0.0)	1 (50.0)
cycle 8	0 (0.0)	0 (0.0)	0 (0.0)	1 (100.0)	0 (0.0)	1 (100.0)
cycle 9	1 (100.0)	0 (0.0)	1 (100.0)	0 (0.0)	0 (0.0)	0 (0.0)
cycle 10	1 (100.0)	0 (0.0)	1 (100.0)	0 (0.0)	0 (0.0)	0 (0.0)

Arm 1: gemcitabine alone; Arm 2: gemcitabine + FOLFIRI.3.

Based on the univariate analyses, variables associated with responder profiles and retained to build the propensity score were a primary tumor location at the pancreatic head (yes vs. no), presence of metastatic lymph node (yes vs. no), neutrophils, hemoglobin and platelet rates (dichotomized according to the median value). In multivariate analysis, a primary tumor location at the pancreatic head (OR = 2.72 [95%CI 0.86–9.16]), the presence of lymph node metastases (7.90 [95%CI 1.12–164.12]), a low neutrophils (2.13 [95%CI 0.64–7.25]) and platelets rate (2.77 [95%CI 0.84–9.72]) and a high hemoglobin rate (1.80 [95%CI 0.54–6.10]) were independently associated with partial responder profile but not statistically significant. The area under the ROC curve was equal to 0.76.

### Longitudinal analysis

In Arm 1 (gemcitabine alone) and Arm 2 (gemcitabine + FOLFIRI.3) respectively:

- 18 and 17 patients experienced a QFS of GHS, among the 63 patients retained (30 in Arm 1, 33 in Arm 2).
- 19 and 17 patients experienced a QFS of physical functioning, among the 65 patients retained (30 in Arm 1, 35 in Arm 2)
- 19 and 18 patients experienced a QFS of emotional functioning, among the 63 patients retained (30 in Arm 1, 33 in Arm 2).
- 18 and 17 patients experienced a QFS of fatigue, among the 65 patients retained (30 in Arm 1, 35 in Arm 2).
- 18 and 16 patients experienced a QFS of pain, among the 65 patients retained (30 in Arm 1, 35 in Arm 2).

**Fig 2:**
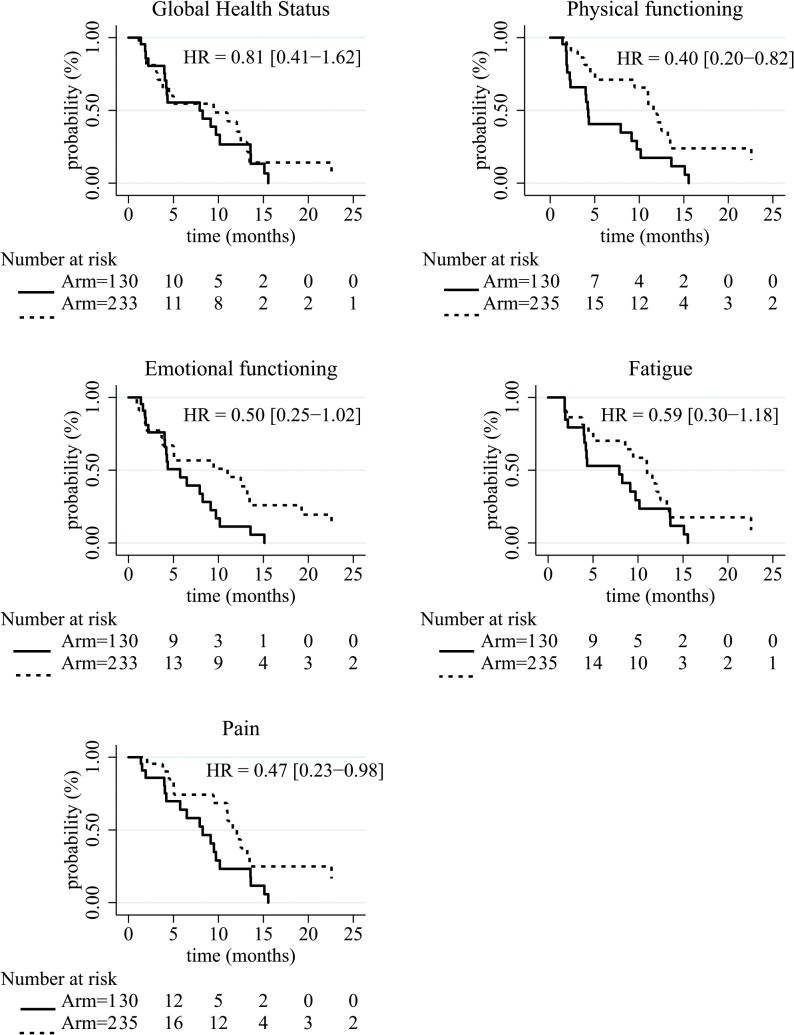
Kaplan-Meier survival curves of the HRQoL deterioration-free survival by treatment arm for the raw and the weighted analysis. Arm 1: gemcitabine alone, Arm 2: gemcitabine + FOLFIRI.3.

**Fig 3:**
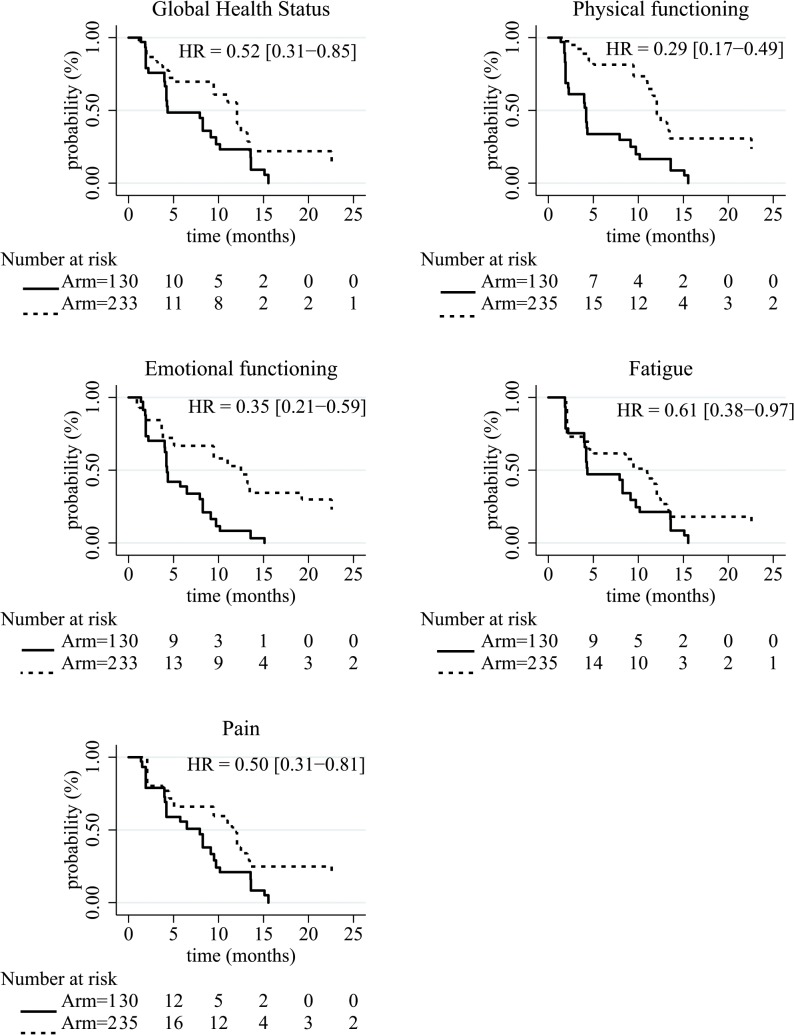
Kaplan-Meier survival curves of the HRQoL deterioration-free survival by treatment arm for the weighted analysis. Arm 1: gemcitabine alone, Arm 2: gemcitabine + FOLFIRI.3.

Regarding the unweighted analysis (Table 4), patients in FOLFIRI.3 + gemcitabine regimen tended to present a longer QFS than those of Arm 1 only for physical functioning (HR = 0.40 [95%CI 0.20–0.82]) with a median QFS of 7.92 months [95%CI 4.21–13.6] for Arm 1 and 9.42 [95%CI 3.81–13.47] for Arm 2. Regarding the weighted analysis, the same result was observed and patients in Arm 2 seemed to present a longer QFS of GHS (HR = 0.52 [95%CI 0.31–0.85]), emotional functioning (HR = 0.35 [95%CI 0.21–0.59]), and pain (HR = 0.50 [95%CI 0.31–0.81]). The median QFS of GHS was 4.34 months [95%CI 4.21–9.72] for Arm 1 and 12.06 [95%CI 9.46–13.47] for Arm 2. The median QFS of emotional functioning was 4.27 months [95%CI 4.04–7.92] for Arm 1 and 12.48 [95%CI 9.46–22.57] for Arm 2. Regarding pain, the median QFS was 7.92 months [95%CI 4.21–9.49] for Arm 1 and 11.60 [95%CI 9.46–13.21] for Arm 2. These QFS curves were described in Figs 2 and 3.

**Table 4:** Results of the Kaplan-Meier estimation of the health-related quality of life deterioration-free survival for a QLQ-C30 score and comparison between treatment arms.

			unweighted analysis		weighted analysis	
		*N* (events)	median	HR [CI 95%]	median	HR [CI 95%]
			[CI 95%]		[CI 95%]	
Global health status	Arm 1 ^a^	30 (18)	7.92 |4.21–13.6]	1	4.34 [4.21–9.72]	1
	Arm 2 ^b^	33 (17)	9.46 |3.81–13.47]	0.81 (0.41–1.62)	12.06 [9.46–13.47]	0.52 [0.31–0.85]
Physical functioning	Arm 1	30 (19)	4.27 [2.27–10.15]	1	4.21 [2.27–7.92]	1
	Arm 2	35 (17)	11.6 [9.46–26.25]	0.40 (0.2–0.82)	12.06 [11.6–22.57]	0.29 [0.17–0.49]
Emotional functioning	Arm 1	30 (19)	5.75 [4.04–9.72]	1	4.27 [4.04–7.92]	1
	Arm 2	33 (18)	11.01 [3.81–22.57]	0.50 (0.25–1.02)	12.48 [9.46–22.57]	0.35 [0.21–0.59]
Fatigue	Arm 1	30 (18)	7.92 [4.21–13.57]	1	4.34 [4.21–9.13]	1
	Arm 2	35 (17)	11.01 [8.57–13.47]	0.59 (0.30–1.18)	10.97 [5.03–12.06]	0.61 [0.38–0.97]
Pain	Arm 1	30 (18)	8.25 [5.75–13.57]	1	7.92 [4.21–9.49]	1
	Arm 2	35 (16)	11.6 [10.97-NA]	0.47 (0.23–0.98)	11.6 [9.46–13.21]	0.50 |0.31–0.81]

^a^ Arm 1: gemcitabine alone;

^b^ Arm 2: gemcitabine+FOLRIRI.3.

Variables retained for the Cox multivariate analysis were treatment arm (Arm 2 vs. Arm 1), number of metastatic sites (2 or more vs. 1) and an interaction effect between treatment arm and the number of metastatic sites, according to the univariate Cox regression analysis (data not shown).

In the unweighted analysis, all the 95%CI contained the value of 1. Regarding the weighted analyses, the treatment arm (gemcitabine + FOLFIRI.3) and the number of metastatic sites (one site) seemed to be independently associated with longer QFS of physical functioning (Table 5). The number of metastatic sites (more than one vs. one) seemed to be associated with a shorter QFS of GHS, fatigue and pain.

**Table 5:** Results of the multivariate Cox regression analysis for the QFS analysis of each targeted score of the QLQ-C30 for the raw and the weighed analysis.

			without IPW	with IPW
		*N* (events)	HR [CI 95%]	HR [CI 95%]
**Global health status**		63 (35)		
arm ^a^	(arm 2) vs.(arm 1)		0.86 [0.38–1.96]	0.58 [0.31–1.07]
number of metastatic sites	(2 or more) vs. 1		3.98 [1.21–13.71]	4.39 [2.03–9.49]
Interaction between arm and number of metastatic sites			0.38 [0.08–1.88]	0.41 [0.13–1.27]
**Physical functioning**		65 (36)		
arm ^a^	(arm 2) vs.(arm 1)		0.34 [0.14–0.82]	0.25 [0.13–0.48]
number of metastatic sites	(2 or more) vs. 1		2.80 [0.87–9.08]	2.71 [1.28–5.75]
Interaction between arm and number of metastatic sites			0.86 [0.18–4.16]	1.09 [0.36–3.30]
**Emotional functioning**		63 (37)		
arm ^a^	(arm 2) vs.(arm 1)		0.44 [0.19–1.03]	0.29 [0.15–0.56]
number of metastatic sites	(2 or more) vs. 1		2.72 [0.84–8.79]	2.59 [1.24–5.41]
Interaction between arm and number of metastatic sites			0.91 [0.19–4.47]	1.47 [0.50–4.37]
**Fatigue**		65 (35)		
arm ^a^	(arm 2) vs.(arm 1)		0.54 [0.23–1.24]	0.71 [0.40–1.24]
number of metastatic sites	(2 or more) vs. 1		3.27 [0.86–12.42]	3.40 [1.58–7.30]
Interaction between arm and number of metastatic sites			0.58 [0.11–3.17]	0.39 [0.13–1.11]
**Pain**		65 (34)		
arm ^a^	(arm 2) vs.(arm 1)		0.44 [0.18–1.07]	0.57 [0.32–1.03]
number of metastatic sites	(2 or more) vs. 1		3.04 [0.93–9.91]	3.15 [1.51–6.57]
Interaction between arm and number of metastatic sites			0.66 [0.13–3.31]	0.46 [0.16–1.33]

^a^ Arm 1: gemcitabine alone, Arm 2: gemcitabine + FOLFIRI.3.

As for the unweighted analysis, the same trends were observed for the sensitivity unweighted analysis integrating non-responders patients (see S1 Fig, S3 and S4 Tables).

## Discussion

As previously reported, patients treated with sequential chemotherapy FOLFIRI.3 + gemcitabine presented a benefit in PFS at 6 months (44.9% (30.7–58.0) vs. 25.7% (14.4–38.6)), OS (64.7%(49.5–76.4) vs. 62.8% (47.6–74.7)) and objective response rate (36.7% vs. 10.2%) [1].

Meanwhile to the recent progress in the improvement of OS, preserving HRQoL is of paramount importance considering the symptom burden and the poor prognosis of mPC. If several Phase III trials attempted to show a clinical benefit or improvement in HRQoL, few have achieved their goals [31,32]. Recently, the clinical trial comparing FOLFIRINOX to gemcitabine shown an improvement in HRQoL for FOLFIRINOX arm [5].

In our trial, HRQoL results support the efficacy profile of FOLFIRI.3 + Gemcitabine regimen. Patients in FOLFIRI.3 + Gemcitabine arm presented a longer QFS than those of gemcitabine alone arm whatever the HRQoL score considered in both QFS analyses even if patients in FOLFIRI.3 + Gemcitabine arm presented twice as much as those of Gemcitabine alone arm occurrence of grade 3 or 4 neutropenia. In multivariate weighted analysis, treatment with sequential FOLFIRI.3 + gemcitabine seemed to be associated with longer QFS in each HRQoL score considered including pain and fatigue score, two symptoms commonly present at time of diagnosis. It would be interested to study the impact of the occurrence of at least one grade 3–4 toxicity on the QFS.

Median QFS for each domain was shorter than median PFS irrespective of the use of IPW method. It is noteworthy that survival estimates depend on the QFS definition. Contrary to our definition, all-cause death was not integrated as an event in the definition of TUDD chosen by Gourgou-Bourgade et al. [5]. In consequence, median TUDD was not reached after a 26.6 months follow-up while the median PFS was 6.4 months in the FOLFIRINOX arm [2,5] which was not in agreement with clinical profiles of these patients. Moreover it is underlined that comparison across trials is not possible, stressing the need to adopt a common definition of TUDD or QFS [9].

If QFS is increasingly used in clinical trials, consensual methods to optimize management of missing data are still lacking [5,33–35]. In FOLFIRINOX trial, little information was provided on the method used to deal with missing data, except when authors declared that the two groups did not differ in terms of rate of missing data [5].

In our study, in both unweighted and weighted analyses, patients in Arm 2 presented a longer QFS than patients in Arm 1. In multivariate analyses, treatment arm (gemcitabine + FOLFIRI.3) and number of metastatic sites (one site) tended to be associated with longer QFS of physical functioning in the weighted analysis. The same trends were observed for the unweighted analysis.

In this way, using the IPW method of the propensity score influences the results of the multivariate analysis by underlining more significant associations. A high weight is assigned to patients with no missing data (mainly patients of Arm 1) and a low weight to partial responders (mainly patients of Arm 2). As in unweighted analysis, a longer QFS was yet observed for patients of Arm 2 as compared to those of Arm 1 for most HRQoL dimensions, the HR increased with the use of the IPW method. The use of the propensity score in conjunction with the TUDD method allowed reducing the bias due to the occurrence of missing data depending on patients’ characteristics during the follow-up. This bias cannot be totally eliminated because missing data can also depend on unobserved data. However, some logistic problems could explain the reasons for partial and non-responders because these patients were followed in the study for other endpoints. Another statistical approach to use in conjunction of the QFS method should be proposed to adequately take into account missing not at random data.

Besides primary prevention procedures for limiting missing data rate, additional work on statistical methods to handle with missing data is still needed. Multiple imputations on the HRQoL scores could also be performed but this method requires a larger sample and can only retain one or two factors associated with missing data [36], more variable can be retained in the propensity score. Then this approach could be suggested for the trials with limited sample size. Contrary to the pattern mixture models, the IPW method in conjunction with the TUDD approach is more appropriate to the design of oncology clinical trials, for which a lot of HRQoL measures are done. In fact, the number of possible patterns increases with the number of HRQoL measures. Austin et al. recommend to use IPW for time to event data [21]. Propensity score matching could also be performed for survival analysis but a higher sample size is needed. Finally, the IPW method is easy understandable (weighting observations according to the presence or absence of missing data) [21].

In conclusion, analyses of QFS supports that sequential strategy with FOLFIRI.3 followed by gemcitabine in patients with untreated mPC is feasible and, despite more toxicities, delayed the HRQoL deterioration. Moreover, using the propensity score allows controlling the imbalance of informative missing data between the two arms and provides more precise estimation of the treatment effect. This sequential treatment strategy will now be compared with FOLFIRINOX in a phase III trial (French study). This phase III clinical trial will allow to confirm or not these results raised from an exploratory analysis.

## Supporting Information

S1 Checklist CONSORT 2010 checklist of information to include when reporting a randomized trial. (DOC)

S1 Authorization English version of the authorization of the study. (DOCX)

S1 Fig Kaplan-Meier survival curves of the HRQoL deterioration-free survival by treatment arm^a^ considering non-responders patients in deterioration since baseline. ^a^Arm 1: gemcitabine alone, Arm 2: gemcitabine + FOLFIRI.3.
(EPS)

S1 Informed Consent French and English version of the informed consent. (DOCX)

S1 Protocol French version of the complete protocol. (DOC)

S2 Protocol English summary of the protocol. (DOC)

S1 Table Health-related quality of life scores at baseline according to treatment arm. (DOC)

S2 Table Most Common Grade 3 or 4 Adverse Events according to treatment arm. (DOC)

S3 Table Results of the Kaplan-Meier estimation of the health-related quality of life deterioration-free survival for a QLQ-C30 score considering non-responders patients in deterioration since baseline and comparison between treatment arms. (DOC)

S4 Table Results of the multivariate Cox regression analysis for the QFS analysis of each targeted score of the QLQ-C30 considering non-responders patients in deterioration since baseline. (DOC)
