# Supplementary material for: Sequential FOLFIRI.3 + Gemcitabine Improves Health-Related Quality of Life Deterioration-Free Survival of Patients with Metastatic Pancreatic Adenocarcinoma: A Randomized Phase II Trial
Source: PLoS One. 2015 May 26;10(5):e0125350. doi: 10.1371/journal.pone.0125350 (PMC4444351; doi:10.1371/journal.pone.0125350)
Supplement: S1 Authorization — (DOCX) [file pone.0125350.s002.docx]

C P P - ll e - d e - F r a n c e V I Groupe Hospitalier Pitié-Salpetriere

CPP n° 22-07

EudraCT : 2006-005703-34

Paris, 7th May 2007

the committee has received the : 19^th^ February 2007

a request for review to the research project:

"A randomized multicenter phase II trial of a regimen of gemcitabine or a sequential chemotherapy FOLFIRI.3 (CPT-11 plus folinic acid plus 5-FU) followed by gemcitabine in patients with previously untreated metastatic pancreatic adenocarcinoma: the FIRGEM Study"

. Protocole 04/20/07

. Investigator leaflet Campto® j uly 2005

. Information note and consent form of the 04/20/07

- Liste des iovestigateu rs du 20/4/07

project sponsored by: AGEO

whose coordinator is: Dr. J.TAIEB

The Committee reviewed the information on this project at its meeting on:

2^nd^ May 2007

Participated in the deliberation:

Claude ANDRE - Allergologue (T) Odile BALAND - Infirmiere (T) Nathalie BRION - Therapeute (S)

Laurent CAPELLE - Neuroch irurgien (T)

Christophe DEMONFAUCON - Representant des associations agreees de malades (T) Marie-Helene FIEV ET - Pharmacien hospitalier (T)

Jean-Louis GOLMARD - Biostatisticien (T) Philippe LECHAT - Pharmacologue (T)

Annie LE FRANC - Representante des associations agreees de malades (T) Fabienne LEVASSEUR - Qualifiee en matiere juridiq ue (T)

Marie-Ceci le MASURE - Psychologue hospital ier (S)

Michele MEUNIER-ROTlVAL - Chercheur en genetique (S) Anne-Laure MORIN - Qualifiee en matiere jur idique (T)

Marti n THrBl ERGE - Neurorad iologue (S)

The comittee adopted the following deliberation: favorable opinion

Motivation: The committee felt that the benefice/risk is acceptable for subjects participating in research.

The Session Chairman


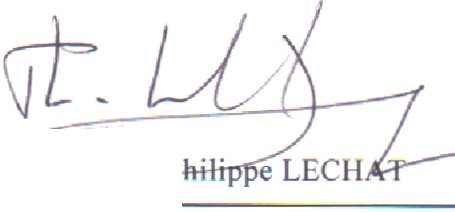


CPP IDF VI 47, Boulevard de l 'Hopital 75013 PARIS 'Je) :0142161683 Fax: 01421627 15
