# Supplementary material for: Sequential FOLFIRI.3 + Gemcitabine Improves Health-Related Quality of Life Deterioration-Free Survival of Patients with Metastatic Pancreatic Adenocarcinoma: A Randomized Phase II Trial
Source: PLoS One. 2015 May 26;10(5):e0125350. doi: 10.1371/journal.pone.0125350 (PMC4444351; doi:10.1371/journal.pone.0125350)
Supplement: S1 Informed Consent — (DOCX) [file pone.0125350.s004.docx]

# Consentement Eclaire (French version)

**essai de phase II randomisé multicentrique d'un traitement par gemcitabine ou d'un TRAITEMENT SEQUENTIEL Par Folfiri.3(CPT-11/Acide folinique/5-FU) / gEMcitabine chez les patients atteints d’un CAncer du pancréas métastatique NON PRE-TRAITE.**

**ETUDE firgem**

Je soussigné(e) NOM : .................................................... Prénom : ................................... certifie avoir été informé(e) par le Dr.................................................. sur le déroulement et l'objet de la recherche intitulée " **Essai de phase II, randomisé, multicentrique d'un traitement par gemcitabine ou d'un traitement séquentiel par FOLFIRI.3(cpt-11/acide folinique/5-fu) / gemcitabine chez les patients atteints d’un cancer du pancréas métastatique non pré-traité. Etude firgem**", évaluant la tolérance et l'efficacité dans ma maladie d’un traitement par l’association **Irinotecan/acide folinique/5-FU** en alternance avec la **gemcitabine**.

Je donne librement mon consentement pour participer à cette étude. Je peux à tout moment décider de me retirer de cette étude, sans avoir de justification à fournir et sans que cet arrêt n'affecte la qualité des soins que je suis en droit d'attendre.

Les objectifs, les bénéfices attendus, les contraintes et les risques m'ont été clairement présentés sur une note écrite qui m'a été remise.

J'ai eu la possibilité de poser les questions qui me paraissaient utiles et de recevoir des réponses claires.

J'accepte que les données me concernant ainsi que les résultats de mon traitement enregistrés à l'occasion de cette étude puissent faire l'objet d'un traitement informatisé, par le promoteur ou pour son compte. J'ai bien noté que le droit d'accès prévu par la loi "Informatiques & Libertés" (article 40) s'exerce à tout moment auprès du Dr ....................................... Je pourrai exercer mon droit de rectification auprès du Dr ...........................................

Je suis affilié à un régime de sécurité sociale.

J'accepte que mon dossier médical soit consulté directement par les Autorités de tutelle ou par le personnel mandaté par le promoteur soumis au secret professionnel. Dans tous les cas, mon nom restera confidentiel.

Si des informations nouvelles concernant l’**Irinotecan, l’acide folinique, le 5-Fu** et la **gemcitabine**, susceptibles de modifier mon consentement, survenaient pendant la durée de l'essai, j'en serais aussitôt averti(e).

Je pourrai demander tout complément d'information, si nécessaire, au Docteur .................................. au numéro de téléphone : ................................

Mon consentement ne décharge en rien l'investigateur et le promoteur de leurs responsabilités morales et légales et je conserve mes droits garantis par la loi.

Un double de ce consentement me sera remis après signature.

| Fait à ………… , le …….    Nom et Signature du Médecin : | Fait à ………… , le …….  Nom et Signature du Patient : |
| --- | --- |

**informed consent (english version)**

**A randomized multicenter phase II trial of a regimen of gemcitabine or a sequential chemotherapy FOLFIRI.3 (CPT-11 plus folinic acid plus 5-FU) followed by gemcitabine in patients with previously untreated metastatic pancreatic adenocarcinoma** **: firgem study**

I undersigned : LAST NAME : .................................................... First name: ................................... certify that I have been informed by Dr.................................................. on the conduct and purpose of the research project entitled "**A randomized multicenter phase II trial of a regimen of gemcitabine or a sequential chemotherapy FOLFIRI.3 (CPT-11 plus folinic acid plus 5-FU) followed by gemcitabine in patients with previously untreated metastatic pancreatic adenocarcinoma : the FIRGEM Study**", evaluating the safety and effectiveness in my illness of a treatment associating **Irinotecan/folfinic acid/5-FU** alternatively with **gemcitabine**.

I freely give my consent to participate in this study. I can at any time decide to withdraw from this study without having to provide justification and without that judgment does not affect the quality of care that I would expect.

Objectives, expected benefits, burdens and risks have been clearly presented to me in a written note that was given to me.

I had the opportunity to ask questions that seemed helpful and receive clear answers.

I agree that my data and the results of my treatment recorded during this study can be processed by computer, by the Promoter or on its behalf. I noted that the right of access under the law “Informatics & Freedoms” (article 40) be exercised at any time from the Dr ....................................... I may exercise my right to the rectification from the Dr ...........................................

I am affiliated to a social security scheme.

I agree that my medical records be consulted by the Trustee Authorities or staff designated by the sponsor legally privileged. In any case, my name will remain confidential.

If new information regarding the **Irinotecan, folinic acid, 5-Fu** and **gemcitabine**, likely to modify my consent, occurred during the duration of the trial, I would be immediately notified.

I can ask for more information if necessary to the Doctor .................................. with the phone number: ................................

My consent shall not release anything the investigator and the sponsor of their moral and legal responsibilities, and I retain my rights under the law.

A copy of this consent will be given to me after signing.

| Done at ………… , date …….    Name and Signature of the Dr: | Done at ………… , date …….  Name and Signature of the patient: |
| --- | --- |
