# Supplementary material for: Sequential FOLFIRI.3 + Gemcitabine Improves Health-Related Quality of Life Deterioration-Free Survival of Patients with Metastatic Pancreatic Adenocarcinoma: A Randomized Phase II Trial
Source: PLoS One. 2015 May 26;10(5):e0125350. doi: 10.1371/journal.pone.0125350 (PMC4444351; doi:10.1371/journal.pone.0125350)
Supplement: S1 Protocol — (DOC) [file pone.0125350.s005.doc]

| **essai de phase II randomisé multicentrique d'un traitement par gemcitabine ou d'un TRAITEMENT SEQUENTIEL Par Folfiri.3(CPT-11/Acide folinique/5-FU)) / gEMcitabine chez les patients atteints d’un CAncer du pancréas métastatique NON PRE-TRAITE. ETUDE firgem** |
| --- |

Essai de phase II randomisé

| **PROMOTEUR : AGEO**    **INVESTIGATEUR-COORDONNATEUR :** Pr Julien TAIEB  Service d’hépato-gastro-entérologie  hopital EuropÉen georges pompidou,  20 rue leblanc 75908 PARIS  Tel : 01.56.09.35.51  Fax : 01. 56.09.35.29  **CENTRE DE RANDOMISATION – ANALYSE :** Franck BONNETAIN (PhD)  Unité de biostatistique et d’épidémiologie  Registre des tumeurs gynécologiques de côte d’Or  DIM  Centre George François Leclerc  1 rue Prof. Marion  BP 77980  21079 DIJON Cedex  Tél : 03 80 73 77 84  Fax : 03 80 73 77 34 |
| --- |

N° eudract : 2006-005703-34

Numéro de la Version : 3-2(01/05/2008) (protocole amendé, amendements n°1 et 2)

Avis favorable du CPP Ile de France 6: lieu :Pitié-Salpêtrière date: 02/05/2007

Autorisation d'Essai Clinique de l'AFSSAPS date: 14/05/2007

LISTE DES ABREVIATIONS ET DEFINITION DES TERMES

| **AMM** | Autorisation de mise sur le marché |
| --- | --- |
| **CA19-9** | Cancer antigen 19-9 |
| **CPM** | Cancer du pancréas métastatique |
| **CPLA** | Cancer du pancréas localement avancé |
| **CPT-11** | Irinotecan ou CAMPTO® |
| **ECG** | Electrocardiogramme |
| **γ GT** | Gamma-Glutamyl Transferase |
| **GEM** | Gemcitabine |
| **GB** | Globules blancs |
| **IRM** | Imagerie par résonance magnétique |
| **ITT** | Intention de traiter |
| **IV** | Intraveineux |
| **LDH** | Lactico-déshydrogénase |
| **LSN** | Limite supérieure de la normale |
| **NCI-CTC** | National Cancer Institute-Common Toxicity Institute |
| **NFS** | Numération Formule Sanguine |
| **OMS** | Organisation Mondiale de la Santé |
| **PAL** | Phosphatases Alcalines |
| **PNN** | Polynucléaires neutrophiles |
| **PS** | Performance Status = indice de performance |
| **RO** | Réponse Objective |
| **TP** | Taux de Prothrombine |
| **5-FU** | 5 –fluorouracile |
| **AF** | Acide folinique |
| **PD** | Maladie Progressive |
| **SD** | Maladie Stable |
| **SC** | Sous cutané |

SOMMAIRE

[1. Résumé du protocole 5](#__RefHeading___Toc176711668)

[2. synopsis of THE PROTOCOL 8](#__RefHeading___Toc176711669)

[3. INVESTIGATEURS ET STRUCTURES ADMINISTRATIVES DE L’ÉTUDE 11](#__RefHeading___Toc176711670)

[4. Données de la littérature et pré-requis 11](#__RefHeading___Toc176711671)

[4.1. Le cancer du pancréas : une pathologie fréquente et grave 12](#__RefHeading___Toc176711672)

[4.2. Gemcitabine : traitement de référence -mais limité- du cancer du pancréas avancé 12](#__RefHeading___Toc176711673)

[4.3. GEMOX et cancer du pancréas avancé : une promesse non tenue 12](#__RefHeading___Toc176711674)

[4.4. Irinotécan : une nouvelle drogue utile dans l’adénocarcinome pancréatique ? 13](#__RefHeading___Toc176711675)

[4.5. « Thérapies ciblées » et cancer du pancréas avancé : une nouvelle déception. 13](#__RefHeading___Toc176711676)

[4.6. FOLFIRI.3 et cancer du pancréas 13](#__RefHeading___Toc176711677)

[4.7. FOLFIRI.3/ gemcitabine en séquentiel : rationnel de l’étude. 14](#__RefHeading___Toc176711678)

[5. BIBLIOGRAPHIE 15](#__RefHeading___Toc176711679)

[6. Objectifs de la recherche (Hypothèses testées, objectif primaire, objectifs secondaires) 18](#__RefHeading___Toc176711680)

[6.1. Objectif principal 18](#__RefHeading___Toc176711681)

[6.2. Objectifs secondaires 18](#__RefHeading___Toc176711682)

[6.3. Critères de jugement : 18](#__RefHeading___Toc176711683)

[7. Plan expérimental 19](#__RefHeading___Toc176711684)

[7.1. Description et Justification 19](#__RefHeading___Toc176711685)

[7.2. Comité de relecture iconographique 19](#__RefHeading___Toc176711686)

[7.3. Sélection de la population étudiée 19](#__RefHeading___Toc176711687)

[7.3.1. Critères d'Inclusion 19](#__RefHeading___Toc176711688)

[7.3.2. Critères de Non Inclusion 19](#__RefHeading___Toc176711689)

[7.3.3. Arrêt des traitements à l’étude 20](#__RefHeading___Toc176711690)

[8. Schéma et conduite de la recherche 20](#__RefHeading___Toc176711691)

[8.1. Durée totale prévisionnelle de la recherche 20](#__RefHeading___Toc176711692)

[8.2. Procédures d'évaluation 21](#__RefHeading___Toc176711693)

[8.2.1. Bilan d’inclusion 21](#__RefHeading___Toc176711694)

[8.2.2. Bilan de surveillance pendant le traitement 21](#__RefHeading___Toc176711695)

[8.2.3. A l’arrêt des traitements à l’étude 22](#__RefHeading___Toc176711696)

[8.2.4. Suivi post-thérapeutique 22](#__RefHeading___Toc176711697)

[8.2.5. Synopsis de la surveillance : 22](#__RefHeading___Toc176711698)

[9. CHARACTERISTIQUES DES TRAITEMENTS A L’ETUDE 23](#__RefHeading___Toc176711699)

[9.1. Présentation des produits 23](#__RefHeading___Toc176711700)

[9.2. Administration des Produits à l'Essai 23](#__RefHeading___Toc176711701)

[9.2.1. ⏺Bras A (bras expérimental) : alternativement, 23](#__RefHeading___Toc176711702)

[9.2.2. ⏺Bras B (traitement de référence) : 23](#__RefHeading___Toc176711703)

[9.3. Durée du traitement 25](#__RefHeading___Toc176711704)

[9.4. Précautions d’emploi 25](#__RefHeading___Toc176711705)

[9.5. Adaptation posologique en fonction de la tolérance: 25](#__RefHeading___Toc176711706)

[9.6. Traitements concomitants 26](#__RefHeading___Toc176711707)

[9.7. Arrêt de la recherche 27](#__RefHeading___Toc176711708)

[10. Critères d’EVALUATION 27](#__RefHeading___Toc176711709)

[10.1. Evaluation de la tolérance 27](#__RefHeading___Toc176711710)

[10.2. Evaluation de l’efficacité anti-tumorale 27](#__RefHeading___Toc176711711)

[11. gestion des donnees statistiques 28](#__RefHeading___Toc176711712)

[11.1. Choix du nombre de patients 28](#__RefHeading___Toc176711713)

[11.2. Description des populations étudiées 29](#__RefHeading___Toc176711714)

[11.3. Analyses statistiques 29](#__RefHeading___Toc176711715)

[11.3.1. Méthodes statistiques 29](#__RefHeading___Toc176711716)

[11.3.2. Description de la population 29](#__RefHeading___Toc176711717)

[11.3.3. Exposition au traitement 29](#__RefHeading___Toc176711718)

[11.3.4. Evaluation de l'efficacité 30](#__RefHeading___Toc176711719)

[12. EVENEMENTS INDESIRABLES - EVENEMENTS INTERCURRENTS 30](#__RefHeading___Toc176711720)

[12.1. Définitions 30](#__RefHeading___Toc176711721)

[12.2. Recueil des événements 31](#__RefHeading___Toc176711722)

[12.3. Procédure à suivre pour la notification des événements indésirables graves 32](#__RefHeading___Toc176711723)

[12.4. Surdosage 33](#__RefHeading___Toc176711724)

[12.5. Arrêt Prématuré de Traitement pour Evénement Indésirable 33](#__RefHeading___Toc176711725)

[13. OBLIGATIONS DE L'INVESTIGATEUR ET DU PROMOTEUR 33](#__RefHeading___Toc176711726)

[13.1. Ethique 33](#__RefHeading___Toc176711727)

[13.2. Modifications du Protocole 34](#__RefHeading___Toc176711728)

[13.2.1. Amendements 34](#__RefHeading___Toc176711729)

[13.2.2. Changements Administratifs 34](#__RefHeading___Toc176711730)

[13.3. Audits et Inspection 34](#__RefHeading___Toc176711731)

[13.4. Considérations Administratives 34](#__RefHeading___Toc176711732)

[13.4.1. Concernant l'investigateur-coordonnateur 34](#__RefHeading___Toc176711733)

[13.4.2. Concernant l'Investigateur 35](#__RefHeading___Toc176711734)

[13.4.3. Concernant le Pharmacien de l'Etablissement 35](#__RefHeading___Toc176711735)

[13.4.4. Concernant le Directeur d'Etablissement 35](#__RefHeading___Toc176711736)

[13.4.5. Concernant le Promoteur 36](#__RefHeading___Toc176711737)

[13.5. Consentement Eclairé du Patient 36](#__RefHeading___Toc176711738)

[13.6. Recueil des Données 37](#__RefHeading___Toc176711739)

[13.7. Conservation des Documents - Identification des Patients 37](#__RefHeading___Toc176711740)

[13.8. Responsabilité et Assurances 38](#__RefHeading___Toc176711741)

[13.9. Confidentialité - Utilisation des Données et Publication des Résultats 38](#__RefHeading___Toc176711742)

[14. ARRET PREMATURE DE L'ETUDE 39](#__RefHeading___Toc176711743)

[ANNEXE I : Liste des investigateurs 41](#__RefHeading___Toc176711744)

[ANNEXE II : Indices de performance 43](#__RefHeading___Toc176711745)

[ANNEXE III : echelle de toxicite nci-ctcae (version 3) 44](#__RefHeading___Toc176711746)

[ANNEXE IV : Resumes des caractéristiques des produits 52](#__RefHeading___Toc176711751)

[IV-1 Irinotécan 52](#__RefHeading___Toc176711752)

[IV-2 Gemcitabine 64](#__RefHeading___Toc176711753)

[IV-3 5-Fluorouracile (5-FU) 71](#__RefHeading___Toc176711754)

[IV-3 5-Fluorouracile (5-FU) 71](#__RefHeading___Toc176711755)

[IV-4 Acide folinique 76](#__RefHeading___Toc176711756)

[ANNEXE V : lettre d'information au patient(V3-1) 78](#__RefHeading___Toc176711757)

[ANNEXE VI : Consentement Eclaire(V3-1) 82](#__RefHeading___Toc176711758)

[ANNEXE VIi : DECLARATION D'EVENEMENT INDESIRABLE GRAVE 83](#__RefHeading___Toc176711759)

[ANNEXE ViII : assurance 85](#__RefHeading___Toc176711760)

[ANNEXE IX : avis du cPP 86](#__RefHeading___Toc176711761)

[ANNEXE X : autorisation de l'AFSSAPS 87](#__RefHeading___Toc176711762)

# Résumé du protocole

| **Titre de l'essai** | **Essai de phase II, randomisé, multicentrique d'un traitement par gemcitabine ou d'un traitement séquentiel par FOLFIRI.3(cpt-11/acide folinique/5-fu) / gemcitabine chez les patients atteints d’un cancer du pancréas métastatique non pré-traité. Etude firgem** |
| --- | --- |
| **Promoteur** | AGEO |
| **Investigateur coordonateur** | Professeur Julien TAIEB  Hôpital Européen Georges Pompidou  20 rue Leblanc 75908 PARIS |
| **Centres investigateurs** | Multicentrique/ AGEO |
| **Objectifs de l'essai** | Objectif principal :   - Taux de survie sans progression à 6 mois   Objectifs secondaires:   - Taux de réponse objective (RECIST) - Survie sans progression - Survie globale - Survie jusqu’à échappement au traitement (pour le bras A) - Tolérance - Bénéfice clinique - Qualité de vie (EORTC QLQ-C30)[[1]](#footnote-2) |
| **Diagnostic & critères principaux d'inclusion et de non inclusion** | - Diagnostic histologique d’adénocarcinome du pancréas sur le primitif ou une localisation métastatique - 18 ≤ âge ≤ 75 ans - espérance de vie > 12 semaines - OMS < 2 - Pas de chimiothérapie antérieure (sauf chimiothérapie adjuvante par gemcitabine si réalisée plus de 12 mois avant l'inclusion). - Pas de radiothérapie antérieure (sauf si terminée depuis au moins 8 semaines avant le début du traitement et s’il existe au moins une cible mesurable en dehors du territoire irradié). - Douleur équilibrée. - Polynucléaires neutrophiles ≥ 1500/mm^3^, plaquettes ≥ 100000/mm^3^, Hb≥ 9g/dl - Bilirubine ≤ 1,5 LSN - ASAT et ALAT < 5 fois LSN - Créatinine ≤ 130 μmol/l ou clairance ≥ 60 ml/min. - Pas de métastase cérébrale ni osseuse connue (pas d’imagerie systématique) - Examen d'imagerie de référence (scanner ou IRM) effectué dans les 3 semaines précédant le début du traitement - Consentement éclairé signé avant inclusion dans l'étude |
| **Méthodologie** | Phase II multicentrique, ouverte, randomisée(technique de minimisation) avec stratification sur les critères suivants :  - Centre  - IP OMS 0 vs 1  - Un vs plusieurs sites métastatiques |
| **Nombre de sujets/patients** | 98 patients |
| **Traitement à l'essai et durée du traitement** | **Les patients seront randomisés (1/1) pour recevoir les traitements suivants :**  **⏺Bras A (bras expérimental): alternativement,**  **- FOLFIRI.3 (4 injections ≈ 2 mois au total)**  J1 : CPT11, 90 mg/m² en perfusion intraveineuse de 60 minutes  J1 : • H_0_ - H_2_ , acide folinique forme dl 400 mg/m² (ou L 200 mg/m²) en Y  • H_2_ - H_48_ , 5-FU perfusion continue de 2000 mg/m²  J3 : CPT11 90 mg/m² administré en perfusion intraveineuse de 60 minutes à H_48_  **Ce traitement sera répété tous les 14 jours pour 4 cures consécutives (J1= J15)**  **Une évaluation clinique, biologique et morphologique est alors réalisée, puis**  **- gemcitabine (6 injections, 1 injection 3 semaines sur 4, soit ≈ 2 mois au total)**  J1 : gemcitabine 1000 mg/m² dans 500 ml de sérum physiologique en perfusion à débit constant 10 mg/m²/min (soit 100 min).  **Ce traitement sera réalisé à J1, J8 et J15 et répété à J29, J36, J43.**  **Une évaluation clinique, biologique et morphologique est alors réalisée.**  **Cette séquence thérapeutique (FOLFIRI.3 suivi de gemcitabine) sera répétée en l’absence de progression ou toxicité limitante lors de l’administration précédente.**  **En cas de progression tumorale ou de toxicité limitante sous l’un de ces deux schémas, l’autre schéma thérapeutique sera poursuivi jusqu’à progression tumorale, toxicité limitante, ou refus du patient.**  **⏺Bras B (traitement de référence) :**  J1 : gemcitabine 1000 mg/m² dans 500 ml de sérum physiologique en perfusion à débit constant 10 mg/m²/min (soit 100 min).  **Ce traitement sera réalisé à J1, J8 et J15 et répété à J29, J36, J43. Une évaluation clinique, biologique et morphologique est alors réalisée.**  **Ce traitement sera poursuivi jusqu’à progression tumorale, toxicité limitante, ou refus du patient (J1= J57).** |
| **Critères d'évaluation** | - Evaluation des toxicités selon les critères NCI-CTG v3.0 à chaque cycle. - Evaluation de l’activité antitumorale tous les 2 mois par scanner, selon les critères RECIST. - On notera la réponse et la progression tumorale obtenue dans chaque bras. De plus, on notera la réponse et la progression tumorale obtenue avec chaque schéma thérapeutique (FOLFIRI.3 **et** gemcitabine) pour le bras A. - La survie sans progression est définie par le temps entre la randomisation et la progression selon les critères RECIST ou le décès, quelque soit le bras de traitement. La survie sans progression sera analysée pour évaluer l’objectif principal de l’étude : taux de survie sans progression à 6 mois. - La survie jusqu'à échappement au traitement sera définie, dans le bras expérimental (bras A), par le temps entre la randomisation et la progression tumorale (RECIST) sous chacun des deux schéma thérapeutique évalué (FOLFIRI.3 **et** gemcitabine) ou le décès Une analyse de sensibilité sera effectuée sur ce paramètre à six mois en utilisant les critères de Fleming. |
| **Suivi post thérapeutique** | Les toxicités liées aux traitements seront analysées jusqu’à trente jours après la dernière administration d’un des traitements à l’étude.  Le patient sera ensuite suivi par le clinicien idéalement tous les 2 mois jusqu’à son décès |
| **Méthodes statistiques** | L’objectif principal de cette étude de phase II est le taux de survie sans progression à 6 mois. Une méthode de Fleming en une étape a été utilisée pour calculer le nombre de patients nécessaire, avec les hypothèses suivantes :  H0 : survie sans progression à 6 mois = 25%  H1 : survie sans progression à 6 mois = 45%  Avec un risque α de type I de 5% et β de type II de 10%, il faut inclure 46 patients.  Au terme de l’étude sur les 46 patients :  - si on observait 15 patients ou moins sans progression ou non décédé (32.6%), la survie sans progression à 6 mois sera significativement inférieure à 25%. Le traitement devra être considéré comme non intéressant pour une étude de phase III.  - si l’on observait au moins 16 patients non progressifs, non décédés (34.8%), la survie sans progression à 6 mois sera significativement supérieure à 25%. Le traitement pourra être considéré comme intéressant pour une étude de phase III.  Un total de 49 patients est nécessaire si l’on considère un risque de perdu de vue ou d’éventuels événements imprévus de 5%.  Soit un total de 98 patients |
|  | Les analyses seront réalisées en intention de traiter.  Les courbes de survie sans progression et globale seront estimées selon la technique de Kaplan-Meier.  Les données de tolérance seront consignées pour tous les patients ayant commencé le traitement, et résumées en fonction du traitement reçu (analyses per protocole).  Les données de tolérance des différents schémas perfusés seront évaluées séparément. |
| **Durée de l'essai** | Durée totale prévue (incluant la période de suivi) est de 30 mois: novembre 2007-juin 2010  Durée des inclusions de 24 mois : novembre 2007-décembre 2009  Durée prévisionnelle de suivi individuelle de 6 à 30 mois.  Analyses finales : 2,5 ans après le début de l’étude (juin 2010). |
| **Assurances** | SM Biomedic |

# synopsis of THE PROTOCOL

| **Title of the trial** | A randomized multicenter phase II trial of a regimen of gemcitabine or a sequential chemotherapy FOLFIRI.3 (CPT-11 plus folinic acid plus 5-FU) followed by gemcitabine in patients with previously untreated metastatic pancreatic adenocarcinoma : the FIRGEM Study |
| --- | --- |
| **Sponsor** | AGEO |
| **Coordinating investigator** | Professeur Julien TAIEB  Hôpital Européen Georges Pompidou  20 rue Leblanc 75908 PARIS |
| **Investigation site** | Multicenter/ AGEO |
| **Objectives** | Primary :   - 6 months progression free survival rate   Secondary:   - Objective response rate - Progression free survival - Overall survival - Survival to treatment failure (arm A) - Tolerance - Clinical benefit - Quality of life (EORTC QLQ-C30)[[2]](#footnote-3) |
| **Target population and eligibility criteria** | - Pathologically proven pancreatic adenocarcinoma (on primitive or metastatic lesion) - 18 ≤ age ≤ 75 years - Life expectancy >12 weeks - Performance status OMS < 2 - No prior chemotherapy (adjuvant chemotherapy by gemcitabine is permited if it was realised more than 12 months before the inclusion) - No prior radiation therapy (except if there is at least one mesurable target outside irradiation area) - Pain well controlled before the inclusion - Neutrophiles ≥1500/mm^3^, platelets ≥ 100000/mm^3^, Hb ≥9g/dl - ASAT and ALAT < 5 UNL - Serum bilirubin ≤ 1,5 UNL - Normal renal function test (serum creatinine concentration ≤ 130 μmol/l or creatine clearance ≥ 60 ml/min) - No known brain or bone metastasis (no need of systematic CTscan) - The exam of reference prints (CTscan or RMN) must be made within 3 weeks before the treatment's initiation - Signed written informed consent obtained prior the inclusion |
| **Method** | Multicentre, randomized, open phase II trial. Randomization will be done using minimization technique with the following stratification criteria :  -Center  -OMS PS 0 vs 1  -One vs >1 metastatic sites |
| **Number of subjects** | 98 patients |
| **Trial design and treatment duration** | **Patients will be randomized (1/1) to receive :**  **⏺ Arm A (experimental arm) : alternately,**  **- FOLFIRI.3 (4 courses per cycle; so ≈ 2 month)**  Day 1 : CPT11, 90 mg/m² intravenously (i.v.) for 60 minutes  Day 1 : • H_0_ - H_2_ , folinic acid dl 400 mg/m² (or L 200 mg/m²)  • H_2_ - H_48_ , continuous infusion of 5-FU at the dose of 2000 mg/m²  Day 3 : CPT11, 90 mg/m² intravenously for 60 minutes at H_48_  **This treatment will be repeated every 14 days (d1=d15)**  **Clinical, biological and morphological will be done after.**    **- GEMCITABINE (6 courses, one course three weeks out of four; so ≈ 2 month per cycle)**  Day 1: gemcitabine at the dose of 1000 mg/m² in 500 ml normal saline infusion at a fixed dose rate of 10 mg/m²/min (i.e. 100 min). This treatment is administered at D1, D8, D15 and at D29, D36, D43.  **Clinical, biological and morphological will be done after.**  **This therapeutic sequence (FOLFIRI.3 followed by GEMCITABINE) will be repeated until disease progression or unacceptable toxicity.**  **The response or the progression with each treatment will be censored. In case of progression or limiting toxicity with one of these two treatments, the other one will be continued until tumoral progression, limiting toxicity or patient’s refusal.**  **⏺ Arm B (reference arm) :**  Day 1: gemcitabine at the dose of 1000 mg/m² in 500 ml normal saline infusion at a fixed dose rate of 10 mg/m²/min (i.e. 100 min). This treatment is administered at D1, D8, D15 and at D29, D36, D43.  **Clinical, biological and morphological will be done after.**  **This treatment will be continued until tumoral progression, limiting toxicity or patient’s refusal (D1=D57).** |
| **Assessment criteria** | - Adverse events are graded according the NCI-CTG v3.0 criteria before each cycle. - Tumor response is evaluated every 2 months by a CT-scan according to the RECIST criteria. - Response and progression will be documented in each arm. Moreover the response or the progression with each treatment (FOLFIRI.3 and gemcitabine) in arm A - Progression free survival is defined in both arm as time from randomisation to first progression using the RECIST criteria or death. Progression free survival will be censored to assess study primary objective : 6 months progression free survival rate. - Survival to treatment failure will be defined for the experimental arm (A) as time from randomisation to time to progression (RECIST) with each treatment (FOLFIRI.3 and gemcitabine), or death. A sensitivity analysis will be performed on this parameter at 6 months using the fleming criteria. |
| **Follow up after study treatment** | Patient will be considered as study outgoing thirty days after last administration of traitment.  Patients' survey will be done by the physician every 2 months for 2 years after the end of treatment or patient’s death. |
| **Statistical analysis** | The primary end point of this phase II study is progression free survival at 6 Months. A Fleming one step design was used to plan this trial. With the following assumptions  H0 : progression free survival at 6 months = 25% (unacceptable efficacy)  H1 : progression free survival at 6 months = 45%  With an α type I error = 5%and a β type II error = 10%, it was required to include 46 patients/arm.  At the end of the study among the 46 patients/arm:  - if we observed 15 or less than 15 patients with no progression or death (32.6%), the 6 Months progression free survival was significantly lesser than 30%. Treatment would be declared as not interesting for a phase III trial  - if we observed 16 or more than 16 patients with no progression or death (34.8%), the 6 Months progression free survival was significantly greater than 30%. Treatment would be declared interesting for a phase III trial  A total of 49 patients/arm is needed considering the risk of 5% of lost of sight patients. Finally it will be necessary to include a total of 98 patients.  The analyses will be performed on intent-to-treat principle.  Progression free survival and survival curves will be estimated using the Kaplan-Meier technique.  The safety data will be presented for all patients who start treatment, summarized according to the actual treatment received. (per protocol analyses)  The safety data of the different infusional schedules will be evaluated separately. |
| **Length of the study** | Overall length study (including the follow up period) is 30months: November 2007–June 2010. Recruitment period is 24 months: November 2007 – December 2009. Individual length survey : 6 to 30 months. Final analysis: 2,5 years after the beginning of the study (June 2010). |
| **INSURANCE** | SM Biomedic |

# INVESTIGATEURS ET STRUCTURES ADMINISTRATIVES DE L’ÉTUDE

Le promoteur de cette étude est l’Association des gastroentérologues oncologues (AGEO).

L’investigateur coordonnateur est le Pr. Julien TAIEB, service d’Hépato-Gastro-Entérologie, Hôpital Européen Georges Pompidou, 20 rue Leblanc, 75908 PARIS

Onze centres participent à cette étude :

- Hôpital de la Pitié-Salpêtrière, Paris 13ème. Service d’Hépato-Gastro-Entérologie. Investigateur :Dr Touraj MANSOURBAKHT
- Hôpital Bichat Claude Bernard, Paris 18ème. Service d’Hépato-Gastro-Entérologie. Investigateur : Dr Thomas APARICIO.
- Hôpital Européen Georges Pompidou, Service d'Hépato-Gastro-Entérologie, Paris 15ème. Co-Investigateurs : Dr Bruno LANDI, Dr Samy LOUAFI, Dr Astrid LIEVRE, Dr Gabriel RAHMI.
- Hôpital d’instruction des Armées du Val de Grâce, Paris 5^ème^. Service d’Hépato-Gastro-Entérologie. Investigateur : Dr Jérome DESRAME.
- Clinique Saint-Jean, Lyon. Investigateurs : Dr Pascal ARTRU et Dr Gérard LLEDO.
- CHU Jean Minoz, Besançon, Service d’Hépato-Gastro-Entérologie. Investigateur : Dr Anne-Claire DUPONT-GOSSARD.
- Polyclinique Jeanne d'Arc, Gien, Service d’Hépato-Gastro-Entérologie. Investigateur : Dr David FALLIK
- CHU de Tours-Hôpital Trousseau, Chambray-Les-Tours. Service d’Hépato-Gastro-Entérologie. Investigateur : Dr Thierry LECOMTE
- Hôpital Antoine BECLERE, Service d'Hépato-Gastro-Entérologie, 92141 CLAMART. Investigateur : Dr Amani ASNACIOS. Co-investigateurs : Pr. Sylvie NAVEAU, Dr. Barbara DAUVOIS, Dr. Nadège BARRI-OVA, Dr. Micheline NIJKE NAKSEU
- Hôpital Bicetre, Service d'Hépato-Gastro-Entérologie, 94275 LE KREMLIN-BICETRE. Investigateur : Dr Anne THIROT-BIDAULT
- Centre hospitalier René DUBOS, Service d'Hépato-Gastro-Entérologie, 95303 CERGY PONTOISE. Investigateur : Dr Sophie MANET-LACOMBE

La surveillance de l’étude et le data management seront réalisés par l’AGEO; la randomisation et l’analyse statistique seront assurées par Franck BONNETAIN (PhD),Unité de biostatistique et d’épidémiologie, Registre des tumeurs gynécologiques de côte d’Or, DIM, Centre George François Leclerc, 1rue Prof. Marion, P 77980, 21079 DIJON Cedex, Tél : 03 80 73 77 84, Fax 03 80 73 77 34.

PFIZER (France) fournira l’irinotécan (CAMPTO®).

Les scanners d'évaluation seront réalisés soit dans le département de radiologie de chaque centre investigateur, soit par un centre de radiologie privé choisi par le patient.

L'évaluation du taux plasmatique d'ACE et du CA19-9 sera réalisée dans le département de biologie de chaque centre investigateur.

Les prélèvements sanguins pour les analyses hématologiques ou biochimiques pourront être réalisés soit par les centres investigateurs, soit en laboratoires privés en ville.

# Données de la littérature et pré-requis

## Le cancer du pancréas : une pathologie fréquente et grave

Le cancer du pancréas est la 5^ème^ cause de décès par cancer dans les pays occidentaux. A cause des difficultés diagnostiques, de l'agressivité de ce cancer et du manque de thérapeutique efficace, seuls 1 à 4 % des patients atteints d'un adénocarcinome du pancréas sont vivants à 5 ans (1). Au moment du diagnostic 50 % des patients ont des métastases viscérales détectables et leur médiane de survie est de 3 à 6 mois. Parmi les autres, près de 10% ont des métastases péritonéales occultes, 30% ont une maladie localement avancée et une médiane de survie de 6 à 9 mois. Seulement 10 à 20 % du groupe initial aura une résection à visée curative, avec une médiane de survie de 15 à 19 mois ; plus de 80% d’entre eux recidivant dans les 2 ans qui suivent la chirurgie (2).

## Gemcitabine : traitement de référence -mais limité- du cancer du pancréas avancé

Pour la majorité des patients métastatiques, la chimiothérapie systémique reste la seule alternative thérapeutique. Dans la plupart des études en mono ou en poly-chimiothérapie chez des patients ayant des maladies avancées, les taux de réponses objectives sont faibles, avec un impact extrêmement modéré sur la survie. En utilisant des critères stricts, le taux de réponses objectives dépasse rarement 20 %(4). La raison de cette chimio-résistance est multifactorielle. Le gène MDR (Multi Drug Resistance) est fortement exprimé dans le pancréas normal et tumoral. Il en est de-même de la Glutathion S-Transferase. L’intérêt d’une chimiothérapie systémique potentiellement toxique chez ces patients, présentant souvent une altération de l’état général marquée et un indice de performance médiocre, a longtemps été discuté.

Depuis 1996, cette attitude thérapeutique est légitimée par une étude randomisée, rapportée par Glimelius et al., ayant montré que l’association 5-FU-acide folinique combinée ou non au VP-16, malgré un faible taux de réponse, augmentait significativement le confort et la survie des patients (3). Le 5-FU seul, ou en association avec le cisplatine, a longtemps été la référence thérapeutique dans cette maladie. Parallèlement, plusieurs études intégrant des données de qualité de vie, ont montré que des patients peuvent avoir une meilleure qualité de vie grâce à la chimiothérapie, sans avoir pour autant une réponse objective à l'imagerie. C’est ainsi que la gemcitabine est devenue la molécule de référence suite à une publication de 1997 montrant son effet bénéfique sur la qualité de vie des malades (5). Néanmoins, les réponses tumorales et la survie sans progression observées restent médiocres et des progrès thérapeutiques sont urgents pour améliorer le devenir de nos patients.

## GEMOX et cancer du pancréas avancé : une promesse non tenue

Nous avons participé à l’élaboration de deux schémas thérapeutiques utilisant deux cytotoxiques modernes : le schéma FOLFIRI.3 (5-FU/AF/CPT-11) (6) et le schéma GEMOX (Gemcitabine/Oxaliplatine) (7).

Le schéma GEMOX comparé à la gemcitabine seule, publié par Louvet et al. a permis d’obtenir à la fois un bénéfice en terme de réponse objective et de survie sans progression ainsi qu’un bénéfice clinique. Bien que cette étude ait atteint son objectif principal (amélioration de la survie globale de 2 mois), la différence n’a pas été significative pour ce paramètre, en raison d’une survie de 7 mois dans le bras contrôle (gemcitabine seule) jamais constatée jusqu’alors. Ceci a conduit à un manque de puissance de l’étude pour faire la démonstration de la supériorité du GEMOX. Ce traitement était néanmoins devenu le standard thérapeutique dans beaucoup de centres français et européens.

Une méta-analyse européenne présentée par Louvet et collaborateurs à l’ASCO cette année a montré chez près de 500 patients que l’ajout d’un sel de platine à la gemcitabine (que ce soit de l’oxaplatine ou du cisplatine) semble apporter un bénéfice en terme de survie globale. Malheureusement, une autre étude de phase III présentée également à l’ASCO 2006 par Poplin et col. n’a pas confirmé de supériorité en terme de survie globale ni de la gemcitabine haute dose à débit constant (1500mg/m² en 150mn) ni de l’association GEMOX par rapport à la gemcitabine seule à 1000mg /m² en 30mn. Le GEMOX n’est donc aujourd’hui pas retenu comme une possibilité thérapeutique en première ligne de traitement du cancer du pancréas métastatique.

## Irinotécan : une nouvelle drogue utile dans l’adénocarcinome pancréatique ?

L’Irinotécan, ou CPT-11, appartient à une nouvelle classe d'agents antimitotiques, les inhibiteurs de l'ADN-topoisomérase de type 1. Son développement a été réalisé essentiellement dans le cancer colorectal (8,9). Les évaluations pré-cliniques et cliniques montrent une activité du CPT-11 dans d'autres cancers, dont l’adénocarcinome du pancréas. Après plusieurs études préliminaires publiées sous forme d'abstracts et une étude de phase II publiée in extenso (9) testant le CPT-11 en monothérapie en traitement de cancers du pancréas localement avancés ou métastatiques, les résultats sont décevants (taux de réponse < 20% et médiane de survie globale < 6 mois) (10).

L’association gemcitabine/ irinotécan, dans 2 études de phase II avait montré une certaine efficacité avec 20% à 25% de taux de réponse pour une toxicité acceptable (11, 12). Mais malgré une augmentation du taux de réponse objective, il n’a pas été observé d’avantage sur la survie globale par rapport à la gemcitabine seule dans une étude randomisée de phase III (13). Récemment, une équipe française a évalué une triple association FOLFIRINOX (irinotécan/oxaliplatine/5-FU) chez 47 patients ayant un cancer du pancréas, métastatique dans 76% des cas, avec des résultats intéressants : 26% de réponse objective, une médiane de survie sans progression de 8.2 mois, et de survie globale de 10.2 mois avec une toxicité non négligeable (52% de toxicités hématologiques de grade 3-4) (14).

Bien que ces résultats soient prometteurs ils restent insuffisants. En effet, aucun des essais randomisés de phase III testant des polychimiothérapies versus la gemcitabine seule n’a permis d’augmenter la survie globale, qui reste très faible de l’ordre de 10 à 20% à 1 an, avec le bras standard. De plus des traitements de deuxième ligne jusqu’à récemment considérés comme inutiles dans cette maladie ont montré des résultats intéressants en terme d’efficacité (15-17) et posent ainsi le problème de la stratégie thérapeutique optimale chez ces patients. Il est donc urgent de trouver de meilleures combinaisons thérapeutiques et de meilleures stratégies pour augmenter la survie des patients atteints de cancer du pancréas métastatique.

## « Thérapies ciblées » et cancer du pancréas avancé : une nouvelle déception.

Une des voies de « recherche clinique», en plein essor en cancérologie, est l’apport des biothérapies utilisées depuis peu en pratique dans d’autres localisations tumorales (colorectale (18), mammaire (19)). Ainsi, plusieurs études de phases II ont testé différentes thérapies ciblées comme l’Avastin® (bevacizumab, anti-VEGF), l’Erbitux® (cetuximab, anti-EGFR) et l’Herceptin® (trastuzumab, anti-HER2-Neu) dans les cancers avancés du pancréas. Les résultats ont été le plus souvent décevants, avec des survies sans progression (PFS) de 3.8 à 5.4 mois, et une survie globale d’environ 7 à 8 mois (20-22). Néanmoins ces traitements sont toujours en cours d’évaluation et aucune conclusion définitive quand à leur efficacité n’est possible aujourd’hui. Van Cutsem et al. - dans un essai randomisé de phase III, en double aveugle, ayant inclus 688 patients - n’ont pas trouvé de gain de survie avec l’association du tipifarnib (inhibiteur de la farnésyltransférase) et de la gemcitabine par rapport au bras contrôle (gemcitabine + placebo) (23). Seul l’essai multicentrique, randomisé, en double aveugle de Moore et al, rapporté sous forme d’abstract à l’ASCO en 2005, retrouve un gain de survie chez les patients ayant reçu de la gemcitabine + Tarceva® (erlotinib, anti-EGF) ; cependant, la survie globale à un 1 an était de 24% dans ce bras, et la différence de survie globale entre les 2 bras était « seulement » de 12 jours au prix d’une élévation majeure des coûts de traitement (24). Plus récemment 2 études randomisées de phase 3 portant sur plus de 600 patients chacune et évaluant les combinaisons gemcitabine plus cetuximab ou bévacizumab en comparaison avec la gemcitabine seule n’ont pas permis de mettre en évidence un bénéfice de l’une ou l’autre de ces 2 biothérapies (*Kindler et al. 4508 ; Philip et Al. A4509 ; ASCO 2007, Chicago)* .

## FOLFIRI.3 et cancer du pancréas

Nous avons testé dans une étude de phase II multicentrique, un schéma optimisé appelé FOLFIRI.3, associant le 5-FU et le CPT-11, où la perfusion de 5-FU est encadrée par des injections d’irinotécan. En effet, les observations de l’INSERM U 417 ont montré l’intérêt d’administrer le CPT-11 à la fois avant et après le 5-FU en raison d’un effet synergique de chaque drogue sur l’autre ; ce schéma a permis d’obtenir des taux de réponse jamais observés ainsi que des survies intéressantes en 2^ème^ ligne de traitement chez des patients atteints de cancer colorectal (25). Dans notre étude, 40 patients atteints de cancer du pancréas métastatique (73%) ou localement avancé (27%), naïfs de tout traitement, ont été inclus. Les résultats ont été très encourageants. En effet, le taux de réponse objective observé était de 37,5%, le taux de contrôle de la maladie (RO et SD) de 65%. Les survies globale et sans progression étaient de 12,1 mois et 5,6 mois, respectivement. Les toxicités de grade 3-4 observées étaient : 35% de neutropénie, 25% de diarrhée; 3 patients ont présenté une neutropénie fébrile grade 4 sans apport de facteurs de croissance. Au total 5 patients ont reçu des G-CSF pendant 13 cycles. Aucune récidive de neutropénie grade 3-4 n’est survenue après réduction de dose et/ou utilisation d’un G-CSF. Aucun décès toxique n’est survenu et la majorité des effets secondaires a pu être maîtrisée par des traitements symptomatiques (6).

## FOLFIRI.3/ gemcitabine en séquentiel : rationnel de l’étude.

Au vu des résultats encourageants obtenus avec le FOLFIRI.3 lors de cette étude et compte tenu des coûts élevés et de l’absence d’argument en faveur d’une efficacité notable des biothérapies dans les premières études effectuées, nous proposons une nouvelle approche pour améliorer le devenir des patients atteints d’adénocarcinome pancréatique évolué. Cette approche se propose d’utiliser les schémas FOLFIRI.3 et gemcitabine en monothérapie, de manière séquentielle, afin d’augmenter la survie des patients dans de bonnes conditions de qualité de vie. En effet, certains auteurs ont rapporté qu’outre le développement de schémas thérapeutiques incluant des nouvelles molécules (CPT-11, oxaliplatine, bevacizumab, cetuximab), l’administration de différents schémas successifs de polychimiothérapies était indépendamment lié à la survie globale des patients atteints de cancers colo-rectaux métastatiques (passant de 10 à 20 mois) (26). L’intérêt de polychimiothérapies séquentielles pour prolonger la survie dans le cancer du pancréas a aussi été évoqué par Klapdor et al. (27). Cette équipe rapporte une médiane de survie exceptionnelle de plus de 12 mois chez 55 patients (en majorité métastatiques) après réalisation de lignes successives de chimiothérapie. Les mécanismes d’action et les profils de toxicité du schéma FOLFIRI.3 et de la gemcitabine pourraient permettre d’éviter les phénomènes de chimiorésistance croisée et de limiter les toxicités, cumulatives ou non, inhérentes à l’un ou l’autre des traitements.

Nous proposons d’associer ces 2 schémas chez des patients porteurs d’adénocarcinome pancréatique métastatique, naïfs de tout traitement, dans le cadre d’une étude de phase II randomisée en comparaison au traitement de référence actuel qui est la gemcitabine. Ainsi la moitié des patients sera traitée par une séquence FOLFIRI.3 pendant deux mois puis de la gemcitabine les 2 mois suivants –et ce jusqu’à progression, toxicité inacceptable ou refus du patient- Un tel schéma permet de traiter la maladie avec 3 molécules efficaces en 4 mois, plutôt que de tester une trithérapie plus coûteuse et surtout plus toxique chez ces patients souvent fragiles. L’autre moitié sera traitée par le traitement de référence actuel, la gemcitabine jusqu’à progression, toxicité inacceptable ou refus du patient-

# BIBLIOGRAPHIE

**1. Gudjonsson B**. Cancer of the pancreas: 50 years of surgery. Cancer Res 1987; 60:2284-303.

**2**. **André T BJ, Louvet C, Gligorov J, Callard P, De Gramont A, GERCOR et Izraël V.** Adénocarcinome du pancréas. Caractères généraux. Presse Med. 1998; 11:533-45.

**3**. **Glimelius B, Hoffman K, Sjoden PO, Jacobsson G, Sellstrom H, Enander LK, et al.** Chemotherapy improves survival and quality of life in advanced pancreatic and biliary cancer. Ann Oncol 1996; 7(6):593-600.

**4**. **Ahlgren JD**. Chemotherapy for pancreatic carcinoma. Cancer 1996; 78(3 Suppl):654-63.

**5. Burris HA 3rd, Moore MJ, Andersen J, Green MR, Rothenerg ML, Modiano MR, Cripps MC, Portenoy RK, Storniolo AM, Tarassoff P, Nelson R, Dorr FA, Stephens CD, Von Hoff DD.** Improvements in survival and clinical benefit with gemcitabine as first-line therapy for patients with advanced pancreas cancer: a randomized trial. J Clin Oncol 1997 Jun; 15(6):2403-13.

**6. Taieb J, Lecomte T, Aparicio T, Asnacios A, Mansourbakht T, Artru P, Fallik D, Spano J, Landi B, Lledo G, Desrame J.** FOLFIRI.3, a new regimen combining 5-fluorouracil, folinic acid and irinotecan, for advanced pancreatic cancer: results of an Association des Gastro-Enterologues Oncologues (Gastroenterologist Oncologist Association) multicenter phase II study. Ann Oncol. 2006 Dec 8

**7. Louvet C, Labianca R, Hammel P, Lledo G, Zampino MG, Andre T, et al.** Gemcitabine in combination with oxaliplatin compared with gemcitabine alone in locally advanced or metastatic pancreatic cancer: results of a GERCOR and GISCAD phase III trial. J Clin Oncol 2005; 23(15):3509-16.

**8. Cunningham D, Pyrhonen S, James RD, Punt CJ, Hickish TF, Heikkila R, et al.** Randomised trial of irinotecan plus supportive care versus supportive care alone after fluorouracil failure for patients with metastatic colorectal cancer. Lancet 1998; 352(9138):1413-8.

**9. Wagener DJ, Verdonk HE, Dirix LY, Catimel G, Siegenthaler P, Buitenhuis M, et al.** Phase II trial of CPT-11 in patients with advanced pancreatic cancer, an EORTC early clinical trials group study. Ann Oncol 1995; 6(2):129-32.

**10. Bleiberg H**. CPT-11 in gastrointestinal cancer. Eur J Cancer 1999; 35(3):371-9.

**11. Rocha Lima C, Savareses D, al.:** A multicenter phase II trial of first-line irinotecan and gemcitabine in patients with locally advanced or metastatic pancreatic cancer. Proc Am Soc Clin Oncol 2000; 19:abstract 1023.

**12. Stathopoulos GP, Rigatos SK, Dimopoulos MA, Giannakakis T, Foutzilas G, Kouroussis C, et al.** Treatment of pancreatic cancer with a combination of irinotecan (CPT-11) and gemcitabine: a multicenter phase II study by the Greek Cooperative Group for Pancreatic Cancer Ann Oncol 2003; 14(3):388-94.

**13. Rocha Lima CM, Green MR, Rotche R, Miller WH, Jr., Jeffrey GM, Cisar LA, et al**. Irinotecan plus gemcitabine results in no survival advantage compared with gemcitabine monotherapy in patients with locally advanced or metastatic pancreatic cancer despite increased tumor response rate. J Clin Oncol 2004; 22(18):3776-83.

**14. Conroy T, Paillot B, Francois E, Bugat R, Jacob JH, Stein U, et al.** Irinotecan plus oxaliplatin and leucovorin-modulated fluorouracil in advanced pancreatic cancer. A Groupe Tumeurs Digestives of the Fédération Nationale des Centres de Lutte Contre le Cancer study. J Clin Oncol 2005; 23(6):1228-36.

**15. Klapdor R, Bahlo M, Babinski A, et al**: Sequential polychemotherapy in exocrine pancreatic cancer. Anticancer Res 2003; 23:841-4

**16. Oettle H, Arnold D, Hempel C, et al**: The role of gemcitabine alone and in combination in the treatment of pancreatic cancer. Anticancer Drugs 2000; 11:771-86.

**17. Tsavaris N, Kosmas C, Skopelitis H, et al**: Second-line treatment with oxaliplatin, leucovorin and 5-fluorouracil in gemcitabine-pretreated advanced pancreatic cancer: A phase II study. Invest New Drugs 2005; 23:369-75.

**18. Hurwitz H, Fehrenbacher L, Novotny W, Cartwright T, Hainsworth J, Heim W, et al.** Bevacizumab plus irinotecan, fluorouracil, and leucovorin for metastatic colorectal cancer. N Engl J Med 2004; 350(23):2335-42.

**19. Piccart-Gebhart MJ, Procter M, Leyland-Jones B, Goldhirsch A, Untch M, Smith I, et al.** Trastuzumab after adjuvant chemotherapy in HER2-positive breast cancer. N Engl J Med 2005; 353(16):1659-72.

**20. Safran H, Iannitti D, Ramanathan R, Schwartz JD, Steinhoff M, Nauman C, et al.** Herceptin and gemcitabine for metastatic pancreatic cancers that overexpress HER-2/neu. Cancer Invest 2004; 22(5):706-12.

**21. Kindler HL, Friberg G, Singh DA, Locker G, Nattam S, Kozloff M, et al.** Phase II trial of bevacizumab plus gemcitabine in patients with advanced pancreatic cancer. J Clin Oncol 2005; 23(31):8033-40.

**22. Xiong HQ, Rosenberg A, LoBuglio A, Schmidt W, Wolff RA, Deutsch J, et al.** Cetuximab, a monoclonal antibody targeting the epidermal growth factor receptor, in combination with gemcitabine for advanced pancreatic cancer: a multicenter phase II Trial. J Clin Oncol 2004; 22(13):2610-6.

**23. Van Cutsem E, van de Velde H, Karasek P, Oettle H, Vervenne WL, Szawlowski A, et al.** Phase III trial of gemcitabine plus tipifarnib compared with gemcitabine plus placebo in advanced pancreatic cancer. J Clin Oncol 2004; 22(8):1430-8.

**24. M. J. Moore DG, J. Hamm, A. Figer, J. Hecht, S. Gallinger, H. Au, K. Ding, J. Christy-Bittel, W. Parulekar.** Erlotinib plus gemcitabine compared to gemcitabine alone in patients with advanced pancreatic cancer. A phase III trial of the National Cancer Institute of Canada Clinical Trials Group [NCIC-CTG]. Proc Am Soc Clin Oncol 2005 (Abstract1).

**25. M. Mabro PA, M. Flesch, F. Maindrault-Goebel, T. Andre, G. Lledo, B. Landi, T. Aparicio, P. Colin, A. De Gramont, GERCOR; Hopital Foch, Suresnes Cedex, France; Clinique St Jean, Lyon, France; Hopital Devron, Dijon, France; Hopital St Antoine, Paris, France; Hopital Tenon, Paris, France; Hopital Georges Pompidou, Paris, France; Hopital Bichat, Paris, France; Clinique Courlancy, Reims, France.** Irinotecan, 5-fluorouracil infusion and leucovorin, (FOLFIRI-3) in pretreated patients with metastatic colorectal cancer: Results of a multicenter phase II study. Proc Am Soc Clin Oncol 2003; l 22:280 (abstr 1125).

**26. Kelly H, Goldberg RM**. Systemic therapy for metastatic colorectal cancer: current options, current evidence. J Clin Oncol 2005; 23(20):4553-60.

**27. Klapdor R, Bahlo M, Babinsky A.** Further evidence for prolongation of survival of pancreatic cancer patients by efficacy orientated sequential polychemotherapy (EOSPC) based on serial tumor marker determinations (CA 19-9/CEA). Anticancer Res 2005; 25(3A):1687-91.

# Objectifs de la recherche (Hypothèses testées, objectif primaire, objectifs secondaires)

## Objectif principal

- Evaluer le taux de survie sans progression à 6 mois.

## Objectifs secondaires

- Evaluer le taux de réponse objective

- Evaluer la survie sans progression

- Evaluer la survie globale

- Evaluer la survie jusqu’à échappement au traitement(pour le bras A)

- Evaluer la tolérance

- Evaluer le bénéfice clinique

- Evaluer la qualité de vie (scores de santé globale, capacité physique, douleur et fatigue)

## Critères de jugement :

- Principal :

Le taux de survie sans progression à 6 mois sera défini par le pourcentage de patient vivant sans progression ou décès à 6 mois du début du traitement. L’analyse sera réalisée en intention de traiter.

- Secondaires

- L’efficacité anti-tumorale sera évaluée sur le taux de réponses objectives selon les critères RECIST (« Respons Evaluation Criteria In Solid Tumors »).

- La (ou les) toxicité(s) du traitement gradée(s) selon les critères NCI-CTC V3.0.

- La durée des réponses objectives, définie comme le temps entre la première évaluation d’une réponse objective (complète ou partielle) et la progression de la maladie ou le décès chez les patients ayant eu une réponse objective.

- La durée de survie sans progression, définie par la durée entre la date de randomisation et l’observation de la première progression de la maladie ou le décès du patient. Les patients vivants sans progression seront censurés aux dernières nouvelles.

- La survie jusqu'à échappement au traitement sera définie, dans le bras expérimental (bras A), par le temps entre la randomisation et la progression tumorale (RECIST) sous chacun des deux schémas thérapeutiques évalués(FOLFIRI.3 **et** gemcitabine) ou le décès.

- La survie globale définie comme la durée entre la date de première administration du traitement et la date de décès quelqu’en soit la cause.

- Le bénéfice clinique défini par une prise de poids de plus de 10%, une amélioration de l’indice de performance (OMS) d’un point minimum, et une réduction significative (passage au palier inférieur ou diminution de plus de 30% de la dose quotidienne administrée) ou un arrêt des traitements antalgiques initialement pris par le patients.

- Le temps jusqu’à première occurrence d’un OMS grade 3-4 sera défini comme l’intervalle de temps entre la randomisation et l’occurrence d’un OMS grade 3-4 ou le décès. Les patients vivants sans OMS 3-4 seront censurés aux dernières nouvelles.

- La qualité de vie EORTC QLQ-C30 : le temps jusqu’à détérioration définitive (l’intervalle de temps entre la date d’inclusion et la date de diminution définitive du score de qualité de vie (diminution du score de 5 points sans amélioration ultérieure de plus de 5 points ou la date dernières nouvelles) du score de santé globale).

# Plan expérimental

## Description et Justification

Il s’agit d’un essai DE PHASE II

ouvert

randomisé

MULTICENTRIQUE

La randomisation sera effectuée à l’aide de la technique de minimisation avec stratification selon :

1 – Le centre

2 - l’IP OMS 0 vs 1

3 - Un vs plusieurs sites métastatiques

La randomisation sera effectuée via internet grâce au logiciel du Consortium Tenalea (<http://fr.tenalea.net/leclerc/>) disponible 24h/24. Après remplissage des critères d’éligibilité, la randomisation sera réalisée avec affectation d’un numéro d’identification et du bras de traitement. Un e-mail et/ou un fax sera adressé automatiquement à l’investigateur principal de l’étude, à l’investigateur et au méthododologiste. Chaque investigateur disposera d’un code d’accès sécurisé.

## Comité de relecture iconographique

Comité de relecture des documents iconographiques ayant servi à établir la réponse.

## Sélection de la population étudiée

On inclura 98 patients prospectivement.

### Critères d'Inclusion

1. Adénocarcinome pancréatique histologiquement prouvé sur le primitif ou sur une localisation secondaire
2. Maladie métastatique
3. Espérance de vie > 12 semaines
4. Au moins une cible tumorale mesurable selon les critères de RECIST en territoire non irradié
5. 18 ≤ âge ≤ 75 ans
6. Indice de performance inférieur à 2 (OMS<2)
7. Polynucléaires neutrophiles ≥ 1500/mm3, plaquettes ≥ 100000/mm3, Hb ≥ 9g/dl
8. Créatinine ≤ 130 μmol/l ou clairance ≥ 60ml/min
9. Bilirubinémie ≤ 1,5 LSN
10. ASAT et ALAT < 5 LSN
11. Absence de métastase cérébrale ou osseuse connue (pas d’imagerie systématique)
12. Examen d'imagerie de référence (scanner ou IRM) effectué dans les 3 semaines avant le début du traitement
13. Consentement éclairé signé du patient
14. Patient potentiellement compliant aux règles du traitement et du suivi de l'étude
15. Patient inclus, traité et suivi dans le centre investigateur
16. Contraception efficace durant toute la durée du traitement et 4 semaines après la fin de celui-ci

### Critères de Non Inclusion

1. Chimiothérapie antérieure (sauf chimiothérapie adjuvante par gemcitabine si réalisée plus de 12 mois avant l'inclusion)
2. Radiothérapie antérieure (radiothérapie acceptée si terminée depuis au moins 8 semaines avant le début du traitement et s’il existe au moins une cible mesurable en dehors du territoire irradié)
3. Grossesse et allaitement
4. Hypercalcémie non contrôlée ou persistante à l’inclusion
5. Ampullomes de Vater et adénocarcinomes des voies biliaires
6. Maladies inflammatoires chroniques de l'intestin (maladie de Crohn, RCH...) et diarrhées chroniques
7. Occlusion ou sub-occlusion intestinale non résolue
8. Infection sévère non contrôlée
9. Défaillance organique majeure (notamment cardiaque ou respiratoire)
10. Antécédent d'autre cancer de moins de 5 ans (en dehors des tumeurs cutanées baso-cellulaires et du cancer in situ du col de l'utérus traités de façon adéquate et à visée curative)
11. Autre traitement antitumoral concomitant
12. Patients dont le suivi régulier est impossible pour des raisons psychologiques, familiales, sociales ou géographiques
13. Participation à un autre essai clinique dans les 30 jours précédant l’inclusion

### Arrêt des traitements à l’étude

**Motif d’arrêt de traitement :**

- Une progression tumorale objective
- Une toxicité inacceptable
- Retard de traitement de plus de 21 jours du fait de la non récupération d’une toxicité digestive ou hématologique de grade > 2
- Refus d’un des traitements par le patient

Pour le bras A, chacun des schémas thérapeutiques (FOLFIRI.3 ou gemcitabine) sera arrêté individuellement si le patient présente l’un de ces motifs.

Tous les traitement à l’étude seront arrêtés si l’un de ces motifs est rempli sous FOLFIRI.3 **et** sous gemcitabine.

Tout patient sera suivi après la fin des traitements à l’étude idéalement tous les 2 mois jusqu’au décès.

En cas de sortie du protocole, l’attitude thérapeutique ultérieure est laissée au choix de l’investigateur. Néanmoins il ne sera pas possible d’administrer un schéma à base d’irinotecan (n’ayant pas d’AMM dans les tumeurs du pancréas) aux patients randomisés dans le bras B (gemcitabine). Un traitement de deuxième ligne à base d’oxaliplatine ou de cisplatine est en revanche possible. Tout traitement administré sera consigné dans le CRF.

# Schéma et conduite de la recherche

## Durée totale prévisionnelle de la recherche

- La durée planifiée des inclusions (intervalle de temps entre le premier sujet/patient sélectionné et le dernier sujet/patient inclus) est de 24 mois (novembre 2007- décembre 2009).
- L’analyse finales des données de l’étude sera réalisée au moins 6 mois après l’inclusion du dernier patient (juin 2010).
- La durée totale de l’essai sera donc en théorie de 2,5 ans (novembre 2007-juin 2010).

## Procédures d'évaluation

### Bilan d’inclusion

Il doit être réalisé dans les 3 semaines précédant le début du traitement en ce qui concerne l'évaluation tumorale et dans les 7 jours précédant la 1^ère^ administration du traitement en ce qui concerne le bilan clinique et biologique. Il comprend :

#### Un bilan clinique :

- Histoire de la maladie

- Principaux antécédents avec mention des traitements antérieurs et concomitants.

- Examen clinique complet avec appréciation de l'état général (IP, OMS) et mention du poids, taille, surface corporelle

- Mensurations précises des cibles tumorales cliniques éventuelles (nodule de perméation cutané, adénopathie…)

- ECG

- Vérification préalable des critères d’inclusion et d’exclusion

- Questionnaire de qualité de vie EORTC QLQ-C30

#### Un bilan biologique :

- NFS, Plaquettes

- Ionogramme sanguin, urée, créatinine

- Bilan biologique hépatique comprenant GGT, PAL, ASAT, ALAT, Bilirubine totale, libre et conjuguée

- Hémostase complète (TP, TCK, facteur V)

- Dosage sérique du CA19-9 et ACE

- Test de grossesse de moins de 7 jours chez les femmes en age de procréer

#### Un bilan tumoral :

Evaluation des cibles par les examens appropriés. Un scanner thoraco-abdomino-pelvien est recommandé, néanmoins une IRM hépatique peut être réalisée en remplacement du scanner abdominal si elle est jugée plus pertinente par l’investigateur. Ces examens doivent être les mêmes que ceux qui seront pratiqués pour la mesure des cibles lors des bilans d'évaluation de l'efficacité anti-tumorale du traitement à l'étude.

### Bilan de surveillance pendant le traitement

En cas d'anomalie d'un des bilans clinique ou biologique, un contrôle sera réalisé jusqu'à récupération d'un grade <2.

Le calendrier du bilan biologique de surveillance peut être modifié et adapté en fonction des toxicités observées.

#### Avant chaque cure :

- Examen clinique comprenant poids, état général (OMS)

- Evaluation des toxicités observées depuis le cycle précédent

- Bilan biologique (NFS – plaquettes, créatinine, Bilirubine totale, libre et conjuguée)

#### Tous les 2 mois

- Evaluation de l’efficacité anti-tumorale du traitement avec les mêmes critères et le même type d'examen que ceux ayant servi à l'évaluation initiale (TDM et/ou IRM)

- Dosage sérique des ACE, CA19-9, GGT, PAL, ASAT et des ALAT ainsi que le bilan biologique de la cure (NFS – plaquettes, créatinine, Bilirubine totale, libre et conjuguée)

- Questionnaire de qualité de vie EORTC QLQ-C30

### A l’arrêt des traitements à l’étude

- - Un examen clinique complet avec appréciation des toxicités maximales et une vérification de toutes les anomalies biochimiques rencontrées durant le traitement par AF/5-FU/irinotécan ou par gemcitabine seront effectués
  - Questionnaire de qualité de vie EORTC QLQ-C30

**La raison et la date de fin de traitement de tous les patients seront documentées dans le cahier d’observation (c’est à dire, les perdus de vue, les retraits de consentement, toxicité inacceptable, maladie progressive…). L’investigateur essayera de compléter toutes les procédures de fin de traitement, au moment où le patient arrête le traitement**.

### Suivi post-thérapeutique

Un suivi clinique et biologique sera réalisé dans les 4 semaines suivant l’administration du dernier traitement à l’étude afin de documenter de manière exhaustive les toxicités observées.

Un suivi sera ensuite réalisé tous les 2 mois, idéalement jusqu'au décès du patient.

Les patients ayant arrêté le traitement avant la progression devront également être suivis. La date de progression devra être renseignée.

Si une deuxième (voire une troisième) ligne de traitement est pratiquée, elle devra être consignée dans le CRF.

### Synopsis de la surveillance :

|  | ***bilan initial***  ***(entre J-7 et J-1)*** | ***Avant chaque cure*** | ***evaluation tous les 2 mois et en fin de traitement*** |
| --- | --- | --- | --- |
| ***Examen clinique*** | ***+*** | ***+*** | ***+*** |
| ***Poids*** | ***+*** | ***+*** | ***+*** |
| ***Etat général (OMS)*** | ***+*** | *+* | *+* |
| ***Consentement éclairé*** | ***+*** |  |  |
| ***NFS plaquettes*** | ***+*** | ***+*** | ***+*** |
| ***Ionogramme/créatinine*** | ***+*** | ***+*** | ***+*** |
| ***Bilirubine totale et conjuguée*** | ***+*** | ***+*** | ***+*** |
| ***SGOT/SGPT*** | ***+*** |  | ***+*** |
| ***γ GT/PAL*** | ***+*** |  | ***+*** |
| ****Marqueurs tumoraux(ACE, Ca19.9)*** | ***+*** |  | ***+*** |
| ***ECG*** | ***+*** |  |  |
| ****Scanner ou IRM*** | ***+*** |  | ***+*** |
| ****Autres examens nécessaires à l'évaluation de la réponse tumorale*** | ***+*** |  | ***+*** |
| ***Toxicité et effets indésirables*** |  | ***+*** | ***+*** |
| ***Questionnaire de qualité de vie EORTC QLQ-C30*** | ***+*** |  | ***+*** |

**Au maximum dans les 3 semaines précédant le début du traitement*

# CHARACTERISTIQUES DES TRAITEMENTS A L’ETUDE

## Présentation des produits

- la gemcitabine sera fourni par la pharmacie du centre investigateur (AMM dans cette indication)
- Le 5-FU et l’acide folinique seront fournis par la pharmacie du centre investigateur
- L'irinotécan sera fourni par le laboratoire PFIZER sous forme de flacons prêts à l'emploi : flacons de 5 ml contenant 100 mg de produit actif et de 2 ml contenant 40 mg

## Administration des Produits à l'Essai

Les patients seront randomisés (1/1) pour recevoir les traitements suivants :

### ⏺Bras A (bras expérimental) : alternativement,

**- 4 injections de FOLFIRI.3 (≈ 2 mois au total)**

J1 : CPT11, 90 mg/m² en perfusion intraveineuse de 60 minutes

J1 : • H_0_ - H_2_ , acide folinique forme dl 400 mg/m² (ou L 200 mg/m²) en Y

• H_2_ - H_48_ , 5-FU perfusion continue de 2000 mg/m²

J3 : CPT11, 90 mg/m² administré en perfusion intraveineuse de 60 minutes à H_48_

**Ce traitement sera répété tous les 14 jours pour 4 cures consécutives (J1= J15)**

**Une évaluation clinique, biologique et morphologique est alors réalisée.**

**- 6 injections de gemcitabine ( ≈ 2 mois au total)**

J1 : gemcitabine 1000 mg/m² dans 500 ml de sérum physiologique en perfusion à débit constant 10 mg/m²/min (soit 100 min).

**Ce traitement sera réalisé J1, J8 et J15 et répété à J29, J36, J43 du régime gemcitabine.**

**Une évaluation clinique, biologique et morphologique est alors réalisée.**

**Cette séquence thérapeutique (FOLFIRI.3 suivi de gemcitabine) sera répétée en l’absence de progression ou toxicité limitante lors de l’administration précédente.**

**En cas de progression tumorale ou de toxicité limitante sous l’un de ces deux schémas, l’autre schéma thérapeutique sera poursuivi jusqu’à progression tumorale, toxicité limitante, ou refus du patient.**

### ⏺Bras B (traitement de référence) :

J1 : gemcitabine 1000 mg/m² dans 500 ml de sérum physiologique en perfusion à débit constant 10 mg/m²/min (soit 100 min).

**Ce traitement sera réalisé J1, J8 et J15 et répété à J29, J36, J43.**

**Une évaluation clinique, biologique et morphologique est alors réalisée.**

**Ce traitement sera poursuivi jusqu’à progression tumorale, toxicité limitante, ou refus du patient (J1= J57).**

**SCHEMA DU TRAITEMENT SEQUENTIEL(bras expérimental, Bras A)**

Procédure

FOLFIRI.3

4 cures/injections

j0

C4*

FOLFIRI3

C4

+ 14j

Ev1

Procédure

gemcitabine

6 cures/injections

C6*

GEMZAR

C6

+ 14j

Ev2

Ev2=PR, SD

Ev2=PD

Ev1=PR, SD

Ev1=PD

gemcitabine

jusqu’à PD ou toxicité limitante

Ev1=PR, SD

Ev1=PD

Sortie d'essai

Procédure FOLFIRI.3 jusqu’à PD ou toxicité limitante

* Si toxicité limitante ou progression clinique avant évaluation, réaliser l’évaluation et passer à l'autre procédure jusqu’à progression ou toxicité limitante

Ev = évaluation

PR = réponse partielle

PD = progression

Figure 1

SD = réponse stable

## Durée du traitement

La 1ère évaluation sera effectuée après environ 2 mois de traitement soit 6 injections de gemcitabine ou 4 injections de FOLFIRI.3. De nouvelles évaluations de l’efficacité anti-tumorale seront réalisées tous les 2 mois.

1. En cas de réponse objective ou de stabilité le traitement sera poursuivi.
2. En cas de progression tumorale (RECIST) ou de toxicité limitante le traitement sera interrompu ; l’autre schéma thérapeutique sera poursuivi pour les patients du bras A (voire figure 2).
3. En cas de réponse complète ou partielle, la réponse devra être confirmée au minimum 4 semaines après la première constatation de la réponse. La durée du traitement sera discutée au cas par cas.

## Précautions d’emploi

**Toute nouvelle administration du traitement ne sera débutée qu’après avoir vérifié avant chaque cure :**

- La NFS : PNN > 1500/mm^3^ et plaquettes > 100 000/mm^3^.

En cas de non récupération, une NFS sera pratiquée tous les 7 jours jusqu’à obtention des chiffres requis. Dès récupération hématologique, l’administration du traitement sera réalisée. En cas de report de cure de plus de 21 jours après la dernière administration, le patient sortira du protocole.

- L’absence d’insuffisance rénale.

- L’absence d’élévation de la bilirubinémie au delà de 1,5 fois la normale pour les patients traités par FOLFIRI.3 : dans le cas contraire, le FOLFIRI.3 est arrêté jusqu’à récupération d’une bilirubinémie < 1,5 fois la normale. En cas de report de cure de plus de 21 jours après la dernière administration, le patient sortira du protocole.

- L’absence de toute toxicité clinique et en particulier digestive : en cas de diarrhée de grade 3 ou 4, le traitement sera retardé jusqu’à récupération d'un grade <2. En cas de report de cure de plus de 21 jours après la dernière administration, le patient sortira du protocole.

## Adaptation posologique en fonction de la tolérance:

Une modification de dose sera réalisée en cas de toxicité  de grade 3-4 (avec ou sans report de cure). Les reports de cures se feront par tranches de 7 jours. Les modalités d’adaptation de doses de FOLFIRI.3 et de gemcitabine en cas de toxicité hématologique ou non sont décrites dans le tableau n°1.

**Tableau n°1** **Toxicité hématologique ou non : adaptation de doses de FOLFIRI.3 et de gemcitabine**

| **Toxicité/grade NCI** | **1** | **2** | **3-4** |
| --- | --- | --- | --- |
| Anémie | Pas de réduction de doses | Pas de réduction de doses | Pas de réduction de dose |
| Neutropénie  Thrombopénie | Pas de réduction de doses | Pas de réduction de doses*, ** | - 5-FU 1600 mg/m² (48h)  - CPT-11 80mg/m² à J1 et J3  - gemcitabine 800mg/m² |
| Diarrhée | Pas de réduction de doses | Pas de réduction de doses*, ** | - 5-FU Pas de réduction  - CPT-11 80mg/m² à J1 et J3 |
| Autre*** | - | - | - 5-FU 1600 mg/m² (48h)  - CPT-11 80mg/m² à J1 et J3  - gemcitabine 800mg/m² |

* après récupération ( toxicité de grade 0 ou 1)

** en cas de non récupération des toxicités à J14 : digestives (persistance de diarrhée de grade > 2) ou hématologiques (persistance d'un grade > 2 pour les PNN ou d'un grade > 1 à J21 pour les plaquettes), on réalisera les réductions de doses comme pour grade 3-4.

*** sauf : alopécie, syndrome cholinergique, nausées/vomissements en l'absence de traitement adéquat

**L’introduction de facteurs de croissances hématopoïétiques : EPO et G-CSF est autorisée en cas de toxicité hématologique confirmée et en respectant les AMM de ces différents produits.**

## Traitements concomitants

- Compte tenu de la fréquence et de l’importance du syndrome algique associé au cancer du pancréas, une stabilité ou un contrôle des douleurs sous traitement antalgique bien conduit avant inclusion est requis, les modifications du traitement antalgique seront recueillies durant la période de traitement par chimiothérapie.
- Traitement préventif du syndrome cholinergique précoce (sueurs, hypersalivation, troubles visuels, crampes abdominales, diarrhée précoce) par atropine 0,25mg SC en dehors des contre-indications habituelles (glaucome, adénome prostatique).
- Traitement préventif des nausées et vomissements par sétrons et corticoïdes selon les recommandations de l’ASCO/ESMO.
- Traitement de la diarrhée tardive (plus de 24 heures après la perfusion de CPT-11) par lopéramide 4mg (2 gélules) dès la première selle liquide, puis 2 mg (1 gélule) toutes les 2 heures jusqu’à 12 heures après la dernière selle liquide, sans excéder 48 heures de traitement.

Une ordonnance de prescription du traitement anti-diarrhéique sera remise au patient à la sortie du service où le traitement aura été réalisé. **Le lopéramide ne doit pas être prescrit en traitement préventif même pour des patients ayant présenté une diarrhée au cours des traitements antérieurs.**

- Hospitalisation en cas de :

- persistance de la diarrhée au delà de 48h malgré un traitement adéquat par lopéramide,

- diarrhée concomitante à une fièvre,

- diarrhée sévère nécessitant une réhydratation parentérale.

- Antibiothérapie à large spectre (type fluoroquinolones) en cas de :

- neutropénie de grade > 3 associée à la diarrhée tardive,

- diarrhée de grade > 3 ou fébrile.

- Radiothérapie externe :

la possibilité de radiosensibilisation grave justifie qu'un intervalle d'au moins 4 semaines sépare la chimiothérapie par gemcitabine d'une radiothérapie externe. Ce délai peut être raccourci si l'état du patient l'exige.

- Autres traitements :

- Tous les traitements symptomatiques nécessaires au confort du patient sont autorisés à condition que leur nature, leur posologie et leur durée d'administration soient précisées dans le cahier de recueil des données.

Il en est de même des autres traitements spécifiques justifiés par une indication médicale.

- Les systèmes de réfrigération du cuir chevelu sont autorisés.

- Les facteurs de croissance hématopoïétique ne sont pas autorisés de façon préventive.

## Arrêt de la recherche

La gemcitabine est le traitement de référence du cancer du pancréas et plusieurs dizaines de millier de patients ont été traités par cette molécule dans le monde (Chua et al Best Pract Res Clin Gastroenterol. 2006). Son profil de tolérance est donc bien connu. Le FOLFIRI.3 a déjà été testé par notre équipe dans cette population lors d'une étude de phase II antérieure. Son efficacité interessante s'obtient au prix d'une toxicité légèrement supérieure à celle observée avec la gemcitabine mais restant acceptable (Taïeb et al. Ann Oncol 2007). La séquence de ces deux traitements ne devrait pas modifier le profil de tolérance de chacun d'entre eux. De plus dans l'étude de phase II sus citée plus de la moitié des patients ont reçu, après échappement au FOLFIRI.3, une chimiothérapie à base de gemcitabine sans qu'aucune toxicité inattendue ou augmentation de fréquence ou d'intensité des toxicités attendues soient observées.

Néanmoins la recherche pourra être arrêtée:

- En cas de survenue d'un décès précoce (dans les 30 jours suivant le début du traitement) chez plus de 20% des patients quelqu'en soit la cause
- En cas de survenue d'un décès clairement lié au traitement chez plus de 10% des patients
- En cas de survenue d'évènements indésirables graves inattendus chez plus de 25% des patients

Les EIG et à fortiori les décès seront comptabilisés en temps réel lors du monitoring de l'étude.

# Critères d’EVALUATION

## Evaluation de la tolérance

Tous les patients ayant reçu au moins une cure seront évaluables pour la tolérance.

Les toxicités seront évaluées selon la classification NCI-CTG (jointe en annexe).

Tous les autres événements n'entrant pas dans l'échelle de cotation des toxicités NCI-CTG seront rapportés en léger, modéré, sévère ou menaçant le pronostic vital.

Les critères témoignant d'une amélioration du bénéfice clinique (évolution de l'état général (PS), du poids et des symptômes, prise d’antalgiques) seront évalués.

## Evaluation de l’efficacité anti-tumorale

Evaluabilité des patients

Ne seront considérés comme évaluables pour l'efficacité d’un traitement que les patients ayant eu au moins un examen d’évaluation après l’examen morphologique initial, et les patients ayant arrêté le traitement pour progression clinique et/ou radiologique évidente ayant reçus au moins une cure de traitement.

Tous les patients n'ayant pas été évalués et sortis d'étude pour une autre cause que progression seront non évaluables pour l'efficacité.

Critères d'évaluation

La réponse tumorale sera évaluée selon les critères RECIST.

Toutes les réponses objectives doivent être confirmées au minimum 4 semaines après leur première documentation.

Les documents iconographiques ayant servi à l'évaluation de la réponse seront revus par un comité iconographique constitué de deux radiologues indépendants.

Le taux de survie sans progression à 6 mois, la durée de survie sans progression, la durée de réponse au traitement, la survie globale et le bénéfice clinique ont été définis au paragraphe 6.3.

Qualité de vie :

Elle se fera à l’aide de l’EORTC–QLQC-30 (version 3) et du module STO22. Le questionnaire QLQC30 est un auto-questionnaire spécifique du cancer (Aaronson NK, et al. The European Organization for Research and Treatment of Cancer QLQ-C30: a quality-of-life instrument for use in international clinical trials in oncology. J Natl Cancer Inst [85], 365-376. 1993). La validation interne de ce questionnaire, composé de 30 items, a permis d’identifier 15 dimensions et de calculer 15 scores: 5 scores d’aptitude fonctionnelle (capacité physique, aptitude à travailler ou à accomplir toute tâche ménagère, capacité cognitive, état émotionnel, état social), un score de qualité de vie globale, un score de problèmes financiers et 8 scores de symptômes (fatigue, nausée/vomissement, douleur, dyspnée, perturbation du sommeil, perte d’appétit, constipation, diarrhée). Les modalités de réponses aux items varient de 4 à 7 sur une échelle de type Likert. Si au moins la moitié des items de chacun des scores sont renseignés alors les scores peuvent être générés. Les scores de santé globale et fonctionnels varient de 0 (pire) à 100 (meilleure) alors que les scores de symptômes varient de 100 (pire) à 0 (meilleure). Le temps moyen de remplissage est de 10 minutes. (Ringdal GI, Ringdal K. Testing the EORTC Quality of Life Questionnaire on cancer patients with heterogenous diagnoses. *Qual Life Res* 1993; 2: 129–140/).

Les scores de santé globale, capacité physique, de fatigue, de douleur seront plus spécifiquement étudié, une différence de 5 points sera considéré comme la plus petite différence de score ayant un sens clinique.

Elle sera évaluée à l’inclusion, puis à chaque consultation pour bilan d’évaluation tumoral ainsi qu’à la fin du traitement

# gestion des donnees statistiques

Un plan d'analyse statistique détaillé sera établi avant le gel de la base de données.

## Choix du nombre de patients

Un plan en une étape de Fleming a été utilisé pour planifier cet essai. L’objectif principal de cet essai de phase II est la survie sans progression à 6 mois d’une séquence thérapeutique de FOLFIRI-3 et gemcitabine. Le taux de survie sans progression à 6 mois attendu pour ce traitement séquentiel est de 45 %. Les meilleurs taux obtenus dans les essais récents avec la chimiothérapie sont de l’ordre de 30 %. Un taux de 25% est donc choisi comme taux d’efficacité minimal.

Plan en une étape de Fleming :

L’objectif principal de cette étude de phase II est le taux de survie sans progression à 6 mois. Une méthode de Fleming en une étape a été utilisée pour calculer le nombre de patients nécessaire, avec les hypothèses suivantes :

H0 : taux de survie sans progression à 6 mois = 25%

H1 : taux de survie sans progression à 6 mois = 45%

Avec un risque α de type I de 5% et β de type II de 10%, il faut inclure 46 patients par bras.

Au terme de l’étude sur les 46 patients :

- si on observait 15 patients ou moins sans progression ou non décédé (32.6%), la survie sans progression à 6 mois sera significativement inférieure à 25%. Le traitement devra être considéré comme non intéressant pour une étude de phase III.

- si l’on observait au moins 16 patients non progressifs, non décédés (34.8%), la survie sans progression à 6 mois sera significativement supérieure à 25%. Le traitement pourra être considéré comme intéressant pour une étude de phase III.

- la probabilité de conclure à l'inefficacité à l'étape 1 alors que p = 45,0%, est beta1 = 6,0%

- la probabilité de conclure à l'efficacité à l'étape 1 alors que p = 25,0%, est alpha1 = 9,0%

Un total de 49 patients par bras est nécessaire si l’on considère un risque de perdu de vue ou d’éventuels événements imprévus de 5%. Soit un total de 98 patients

## Description des populations étudiées

Population en ITT

Il s'agit de l’ensemble des patients inclus dans l’étude à l’exception de ceux perdu de vue dès l’inclusion

Population des patients évaluables pour la réponse

Voir 10.2

Population des patients éligibles

Il s'agit des patients n'ayant pas de déviations majeures.

Les déviations majeures seront définies dans le plan d'analyse statistique.

Population per protocole

Il s'agit des patients évaluables pour la réponse ayant reçu au moins une cure de traitement et éligibles.

## Analyses statistiques

Un plan d’analyse sera rédigé avant le gel de la base de données. Comme il s’agit d’un phase II, aucune comparaison statistique n’est planifiée.

Toutefois les critères de jugement pourront être comparés selon le traitement à titre exploratoire, les résultats n’auront alors valeur d’hypothèses

### Méthodes statistiques

Les analyses statistiques seront descriptives.

Les variables quantitatives seront décrites par la médiane, le minimum, le maximum, la moyenne et l'écart type.

Les variables qualitatives seront décrites par leurs fréquences et les pourcentages associés.

Les données de survie, paramètres dépendant du temps, seront estimées par la méthode de Kaplan Meier.

### Description de la population

Le nombre de patients dans chaque population (en intention de traiter, évaluable pour la réponse, éligible et per protocole), les déviations au protocole, les raisons d'arrêt de traitement ainsi que les motifs de décès seront décrits.

### Exposition au traitement

Le nombre médian de cycles reçus, ainsi que le nombre de patients à chaque cycle seront décrits.

La dose intensité sera calculée par cycle et par patient.

### Evaluation de l'efficacité

Paramètres d'efficacité : les analyses seront réalisées sur les 2 populations : population en ITT et population des patients évaluables per protocole, à l’exception de la survie globale qui ne sera analysée que sur la population en ITT.

**Critère principal**

Le critère principal est le taux de survie sans progression à 6 mois.

Il sera estimé sur la population en ITT.

L'intervalle de confiance à 95 % sera calculé à l’aide de la méthode exacte.

On calculera également l'intervalle de confiance à 95% du taux de survie sans progression sur la population des patients évaluables et éligibles per protocole.

**Critères secondaires**

La durée de la réponse, le temps jusqu'à progression (ou jusque récidive en cas de résection chirurgicale rendue possible par le traitement), la survie globale, et le temps jusqu’à occurrence d’un OMS Grade 3-4 seront estimés à l'aide de la méthode de Kaplan Meier.

La survie jusqu'à échappement au traitement sera définie, dans le bras expérimental (bras A), par le temps entre la randomisation et la progression tumorale (RECIST) sous chacun des deux schéma thérapeutique évalué (FOLFIRI.3 **et** gemcitabine) ou le décès. Une analyse de sensibilité sera réalisée sur ce paramètre à 6 mois en utilisant les critères décisionnels de Fleming.

Le pourcentage de patients devenus résécables et mis en rémission complète après chirurgie sera estimé.

On décrira également la survie sur le sous-groupe des patients réséqués.

Chacun des scores de qualité de vie sera décrit à l’aide de la moyenne (standard deviation), de la médiane et de l’étendue (Minimum – Maximum). Le caractère aléatoire des données manquantes sera exploré. Des modèles mixtes d’analyse de variance pour mesures répétées permettront d’étudier l’évolution temporelle de chacun des scores. Le temps jusqu’à détérioration définitive sera définit comme l’intervalle de temps entre la date d’inclusion et la date de diminution définitive du score de qualité de vie (diminution du score de 5 points sans amélioration ultérieure de + de 5 points) ou la date de dernières nouvelles.

**Analyse de la tolérance**

La tolérance sera étudiée sur la population en ITT.

Les toxicités hématologiques et non hématologiques seront décrites par cycle et par patients, selon leur grade NCI-CTCAE V 3.0 (cf annexe n°III), leur imputabilité et leur sévérité.

# EVENEMENTS INDESIRABLES - EVENEMENTS INTERCURRENTS

## Définitions

Un événement indésirable est tout symptôme, signe, maladie ou réaction qui se développe ou s'aggrave pendant la durée de l'essai. Toute maladie ou accident (blessure) intercurrent doit être considéré comme un événement indésirable. Des résultats anormaux d'examens paracliniques sont considérés comme des événements indésirables si l'anomalie observée :

- entraîne une sortie d'essai,
- est associée à un événement indésirable grave,
- est associée à des signes cliniques ou des symptômes,
- nécessite un traitement ou des examens complémentaires,
- est considérée par l'investigateur comme ayant une signification clinique,
- Les événements indésirables sont classés en graves ou mineurs.

Un événement indésirable grave répond à l'un au moins des critères suivants :

- est fatal,
- met en jeu le pronostic vital,
- nécessite une hospitalisation ou une prolongation de l'hospitalisation (pour un patient déjà hospitalisé),
- entraîne une invalidité ou une incapacité,
- est une anomalie congénitale,
- est un événement médical important,

quel que soit le caractère attendu ou inattendu de l'événement constaté, et la relation avec le produit à l'essai.

Un événement médical important est un événement qui peut ne pas mettre immédiatement en jeu le pronostic vital, mais qui, à l'évidence a une grande signification clinique. Il peut faire courir un risque au patient et nécessiter une intervention médicale pour prévenir une issue correspondant à l'un des critères de gravité mentionnés ci-dessus (ex. : hépatite traitée à domicile).

*Un cancer, une grossesse, un surdosage ou un abus de médicament doit normalement être considéré comme grave.*

Un événement indésirable mineur est un événement qui ne présente pas de critères de gravité.

*Une hospitalisation programmée avant le début de l'essai n'est pas un événement indésirable grave.*

Le promoteur déclarera à l’agence du médicament tout événement indésirable grave susceptible d’être dû à la recherche.

Du fait de la gravité de la maladie dans cette étude, certaines conditions définies comme EIG seront exclues de la procédure de déclaration d’un EIG, soit :

- Hospitalisation ou chirurgie liées spécifiquement au traitement de la maladie.

- Hospitalisation réalisée pour simplifier les traitements ou les procédures de l’étude.

## Recueil des événements

La période de l'essai pour recueillir les événements indésirables est normalement définie comme suit : de la date d'inclusion à 30 jours après la dernière administration du traitement à l'étude.

A chaque contact avec le(s) patient(s), l'investigateur recherchera systématiquement des événements indésirables/intercurrents par questionnaire (ouvert ou spécifique) et, si nécessaire, par des examens cliniques ou biologiques. Tous les événements indésirables survenant pendant la durée de l'essai doivent être enregistrés. Les informations sur les événements indésirables/intercurrents devront être reportées immédiatement sur le cahier d'observation à la place prévue à cet effet : module d'événements indésirables/intercurrents pour tous les événements plus le formulaire spécial pour les événements indésirables graves. Tous les signes, symptômes et procédures diagnostiques anormales reliés entre eux devront chaque fois que possible être groupés et enregistrés sous un diagnostic unique ; les composants du diagnostic pouvant être décrits pour vérification.

L'évolution de chaque événement sera suivie jusqu'à guérison ou retour à l'état antérieur, ou stabilisation ou décès ou jusqu'à ce qu'il soit clairement établi que l'événement n'est pas lié au produit à l'essai, ni à la recherche en cours.

Les événements indésirables graves qui sont toujours en cours à la fin de la période de l'essai doivent être suivis pour en déterminer l'évolution.

Tout événement indésirable qui survient après la période de l'essai et qui est considéré comme possiblement relié au produit à l'essai ou à la recherche, doit être notifié immédiatement au Promoteur.

## Procédure à suivre pour la notification des événements indésirables graves

En cas de survenue d'un événement indésirable grave, le patient doit être traité de façon appropriée et cela peut justifier l'arrêt du traitement à l'essai.

Tout événement indésirable grave, y compris le décès quelle qu'en soit la cause, survenant à un moment quelconque de l'essai, fera l'objet d'une investigation selon la procédure suivante :

Tout événement indésirable grave doit être rapporté dans les 24 heures par téléphone ou par fax ou, au plus tard, le jour ouvré (travaillé) suivant.

Les personnes à contacter sont : Professeur Julien TAIEB

Service d’hépato-gastro-entérologie

Hôpital Européen Georges Pompidou,

20 rue Leblanc 75908 PARIS

Tél : 01.56.09.35.51/50.42

Fax : 01.56.09.35.29

Dr. Astrid LIEVRE

Service d’hépato-gastro-entérologie

Hôpital Européen Georges Pompidou,

20 rue Leblanc 75908 PARIS

Tél : 01.56.09.35.51

Fax : 01.56.09.35.29

Fabrice LACAN

Service d’hépato-gastro-entérologie

Hôpital Européen Georges Pompidou,

20 rue Leblanc 75908 PARIS

Tél : 01.56.09.35.51

Fax : 01.56.09.35.29

Dès que l'investigateur est informé de l’événement, il le signale immédiatement au Promoteur par téléphone ou par fax.

L'investigateur complète le formulaire spécial pour les événements graves et l’adresse par fax dans les 24 heures ou, au plus tard, le jour ouvré (travaillé) suivant au responsable de l’essai. Un minimum d'informations devra être fourni à savoir :

- n° de l'essai,
- n° du centre,
- n° du patient/sujet,
- date de début de traitement, (phase de l'essai pendant laquelle est survenu l'événement),
- la description de l'événement,
- la date de début et l'état clinique actuel,
- le motif de gravité,
- la causalité de l'investigateur,
- l'issue.

L’investigateur fournit également toute autre information disponible (antécédents, traitements concomitants, examens complémentaires....) qui pourrait aider à la compréhension de l'événement.

L'investigateur complète si nécessaire la déclaration initiale, c’est-à-dire si celle-ci ne portait pas toutes les informations demandées, et l'adresse, le plus rapidement possible, au promoteur.

L'investigateur transmet par la ensuite un suivi de déclaration initiale mentionnant les résultats de tout examen approprié et le compte rendu des traitements réalisés par l'investigateur et/ou d'autres médecins.

Les informations ainsi recueillies seront communiquées au promoteur, selon les mêmes règles, au fur et à mesure de leur disponibilité.

Une visite de suivi spécifique pourra être réalisée par le promoteur.

## Surdosage

En cas de surdosage et même si l'état clinique du patient ne s'en trouve pas affecté, la procédure d’événements graves décrite ci-dessus sera suivie.

Aucun cas de surdosage n'a été rapporté à ce jour. Au cours des phases I, des hautes doses de Campto® de 750mg/m² ont été administrées. Les toxicités rapportées étaient des neutropénies sévères et des diarrhées. En cas de surdosage, les patients devront être surveillés dans un service spécialisé; Il n'existe pas d'antidote.

## Arrêt Prématuré de Traitement pour Evénement Indésirable

Tout patient qui présente un événement indésirable peut sortir de l'essai sur sa demande ou/et à la discrétion de l'investigateur, du promoteur ou des autorités de santé.

Si l'événement indésirable est en rapport avec un surdosage du produit à l'essai l'investigateur devra se reporter à la brochure investigateur pour connaître et prendre les mesures spécifiques.

Si un patient est retiré de l'essai pour événement indésirable, les pages spécifiques de fin d'essai du cahier d'observation devront être complétées, en plus des modules d'événements intercurrents. Le promoteur de l'essai devra être informé immédiatement de tous les arrêts pour événement indésirable.

# OBLIGATIONS DE L'INVESTIGATEUR ET DU PROMOTEUR

Cet essai doit être mené conformément à la Législation en vigueur, aux standards des Bonnes Pratiques Cliniques (*ICH E6, GCP recommandations Mai 1996*) et en accord avec la dernière révision de la déclaration d’Helsinki (amendement Octobre 1996 République d’Afrique du Sud).

## Ethique

Le présent protocole, le modèle de consentement et la notice d'information au patient, doivent être soumis à un Comité de Protection des Personnes (CPP). Celui-ci délivrera un avis quant à la conduite de l’essai. Cet avis mentionnera la date de délibération, la liste des membres ayant participé à la délibération, les éléments authentifiant la version du protocole, du consentement et de la notice d'information au patient qui lui ont été soumis. Il sera joint au dossier de demande d'autorisation d'essai clinique adressé à l'Agence Française de Sécurité Sanitaire des Produits de Santé (AFSSAPS). L'essai clinique ne pourra débuter qu'après obtention à la fois de l'avis favorable du CPP et de l'autorisation d'essai clinique accordée par l'AFSSAPS.

Une copie de l'avis du CPP et de l'autorisation d'essai clinique accordée par l'AFSSAPS seront fournies à tous les centres participants. Ces informations seront également délivrées à tous les patients avant leur inclusion.

Il incombe à l'investigateur-coordonnateur d'informer le CPP et l'AFSSAPS de tout fait nouveau significatif concernant le produit à l'essai.

## Modifications du Protocole

Le Promoteur est seul autorisé à modifier le protocole.

### Amendements

Un amendement est défini comme une "modification substantielle" du protocole, c’est-à-dire toute modification qui affecte la conduite de l'essai ou la sécurité des patients, incluant des modifications des objectifs de l'essai, du schéma de l'essai, de la population, des procédures de l'essai, des aspects administratifs importants.

Tout amendement doit faire l'objet d'une procédure identique à celle décrite au paragraphe 13.1. Un exemplaire de l'amendement est ensuite adressé à tous les investigateurs participant à l'essai à fin de signature.

En cas d'amendement, le protocole fait l'objet d'une révision. La version amendée du protocole est alors transmise à tous les investigateurs.

### Changements Administratifs

Un changement administratif est défini comme une modification mineure du protocole ou une clarification sans retentissement sur la conduite de l'essai.

Le changement administratif fait l'objet d'un agrément entre le Promoteur et l'investigateur-coordonnateur.

Il peut être communiqué au CPP et à l'AFSSAPS.

## Audits et Inspection

La participation à cet essai implique l'acceptation d’éventuels audits émanant du Promoteur ou d'une Inspection éventuelle des Autorités de Santé françaises ou étrangères.

## Considérations Administratives

### Concernant l'investigateur-coordonnateur

L'investigateur-coordonnateur de cet essai multicentrique est le **Professeur TAIEB.**

Il est l'un des investigateurs participants.

L'investigateur-coordonnateur soumet le protocole et les éventuels amendements à l'avis du Comité de Protection des Personnes (CPP) île de France 6 et à l'autorisation d'essai clinique de l'AFSSAPS, participe aux réunions organisées au cours de l'essai, est responsable de la transmission au CPP et à l'AFSSAPS de tout fait nouveau significatif concernant le produit à l'essai.

### Concernant l'Investigateur

L'investigateur est un médecin gastro-entérologue oncologue.

L'investigateur s'engage à respecter pendant l'essai les dispositions légales et réglementaires qui lui incombent et notamment celles du Code de la Santé Publique, du Code des Assurances, du Code de la Sécurité Sociale et du Code du Travail.

L'investigateur est responsable du déroulement de l'essai dans son centre. L’investigateur accepte de faciliter tout contrôle sur les lieux de l'essai (visites de suivis effectuées par un représentant du Promoteur, audit à la demande du Promoteur ou inspection émanant des Autorités de Santé françaises ou étrangères), il accepte l’accès direct aux documents sources (c’est-à-dire dossier médical, originaux des examens de laboratoire, livre des rendez-vous, etc.) et doit réserver un temps suffisant pour ces visites.

L'investigateur s’engage :

- à fournir au Promoteur au début de l'essai :
- son curriculum vitae daté et signé,
- un exemplaire, daté et signé, du protocole de l’essai,
- un exemplaire, daté et signé, de la convention financière,
- un accusé de réception des produits qui lui ont été remis par le promoteur (sauf pour les établissements de soins).
- à réaliser l'essai comme défini dans le protocole :
- recueillir le consentement éclairé de chaque patient (*voir paragraphe 13.5 - Consentement Eclairé du Patient*),
- assurer la conservation des produits qui lui ont été confiés, en accord avec les B.P.C. et dispenser ces produits conformément au protocole et en accord avec la législation en vigueur (sauf les établissements de soins),
- enregistrer les données correctement dans le cahier d'observation (*voir paragraphe 13.6 - Recueil des Données*).
- à remettre en fin d’essai au Promoteur :
- les produits utilisés ou non, ainsi que les emballages vides, sauf cas particulier,
- les informations relatives à l'évolution après l'essai, d'éventuels événements indésirables graves observés au cours de l'essai, jusqu'à guérison ou retour à l'état clinique antérieur du patient.
- à conserver en archive les documents relatifs à l'essai aussi longtemps que l'exige la législation en vigueur (*voir paragraphe 13.7 - Conservation des Documents*).

### Concernant le Pharmacien de l'Etablissement

Le pharmacien assure la conservation des produits qui lui sont confiés en accord avec les BPC et dispense les produits destinés à l'essai conformément au protocole et en accord avec la législation. Il doit fournir l'accusé de réception des produits destinés à l'essai.

### Concernant le Directeur d'Etablissement

Le directeur d’établissement établit une convention prenant en charge les frais supplémentaires occasionnés par l'essai, conformément aux informations transmises par le Promoteur.

### Concernant le Promoteur

Le Promoteur informe le Directeur de l'Etablissement des modalités de réalisation de l'essai, avant le début de l'essai, et ce conformément à la législation en vigueur en France.

Le Promoteur fournit au Pharmacien Hospitalier (si applicable):

- les informations sur l'essai et le produit,
- les produits destinés à l'essai, la formule qualitative et quantitative du produit à l'essai ainsi que tous les documents requis par les autorités en ce qui concerne les produits à l'essai.

Le Promoteur fournit à l'Investigateur :

- avant le début de l'essai :
- un classeur investigateur comprenant :

- la brochure de l'investigateur actualisée contenant les données biochimiques, pharmacologiques, toxicologiques et cliniques disponibles sur le produit,

- le protocole de l'essai et ses annexes,

- une copie de l'attestation d'assurance,

- une copie de l’avis du CPP ,

- une copie de l'autorisation d'essai clinique donnée par l'AFSSAPS,

- la liste des investigateurs pressentis pour participer à l'essai,
- les cahiers d'observation à remplir,
- la notice d'information donnée au patient,
- les formulaires de consentement éclairé par écrit,
- au cours de l'essai :
- toute information non publiée qui peut influer sur le déroulement de l'essai,
- toute mise à jour de la brochure de l'investigateur,
- les mises à jour de la liste des investigateurs participant à l'essai,
- une copie d’éventuels amendements/changements administratifs,
- une copie de l’avis du CPP concernant d’éventuels amendements,
- une copie de l'autorisation d'essai clinique donnée par l'AFSSAPS concernant d’éventuels amendements.

Le Promoteur s’assure du bon déroulement de l’essai dans les différents centres investigateurs. Cet essai est suivi par un représentant du Promoteur : l'Assistant de Recherche Clinique. Des visites sur les lieux d'essai sont faites avant le début de l'essai et à intervalles réguliers pendant l'essai. En outre, les communications téléphoniques et le courrier sont utilisés en fonction des nécessités.

Le Promoteur a le droit d'arrêter prématurément l'essai.

## Consentement Eclairé du Patient

Préalablement au recueil du consentement du patient, l'investigateur est chargé d'informer le patient :

- de l'objectif de l'essai, de sa méthodologie, de sa durée,
- des bénéfices attendus, des contraintes et des risques prévisibles, y compris en cas d'arrêt de l'essai avant son terme,
- de l'avis du CPP
- de l'autorisation d'essai clinique donnée par l'AFSSAPS.

Ces informations sont données oralement et résumées par écrit en des termes et dans un vocabulaire que le patient peut comprendre. Le résumé écrit des informations données au patient est soumis à l'avis du CPP et à l'autorisation d'essai clinique donnée par l'AFSSAPS.

L'investigateur informe le patient de son droit d'arrêter sa participation à l'essai à tout moment sans avoir à donner de justification.

L'investigateur est tenu d'obtenir par écrit le consentement éclairé de chaque patient avant son inclusion dans l'essai, c’est-à-dire avant démarrage de toute procédure imposée par le protocole, et ce conformément à la législation en vigueur en France. Un exemplaire du formulaire de consentement éclairé signé est remis au patient.

Dès l'inclusion, l'investigateur envoie au Promoteur le formulaire de déclaration d'inclusion attestant le recueil du consentement écrit du patient. Le formulaire de consentement éclairé complété par écrit par le patient est présenté à l'Assistant de Recherche Clinique du Promoteur*,* et un double lui est remis sous enveloppe cachetée, pour archivage dans le dossier de l'essai**.**

Ces consentements sont conservés par l’investigateur, comme l'exige la législation en vigueur.

## Recueil des Données

Un cahier d'observation (fourni par le Promoteur) est nécessaire pour tout patient ayant été inclus. Ce cahier d'observation est conçu pour une saisie et une analyse informatique. Toutes les données doivent être enregistrées pour en permettre une interprétation correcte. Les cahiers d'observation sont remplis au stylo à bille noir, afin de permettre des copies nettes de tous les feuillets des cahiers d'observation. La correction des données sur les cahiers d'observation ne peut être faite qu'en barrant la donnée incorrecte (d'un simple trait) et en écrivant à côté la donnée exacte. Chaque correction doit être paraphée et datée par l'investigateur. La dernière page du cahier d'observation est datée et signée par l'investigateur.

L'investigateur fournit régulièrement au Promoteur les cahiers d'observation ou segments de cahiers d'observation remplis aussi bien pour les observations complètes que pour celles interrompues.

## Conservation des Documents - Identification des Patients

Il est de la responsabilité de l'investigateur de conserver suffisamment d'informations pour permettre de retrouver les patients participants à l'essai et leurs dossiers et être en mesure de les communiquer aux Autorités de Santé ou au Promoteur, si nécessaire.

Les documents relatifs à l'essai sont les suivants :

- l'exemplaire daté et signé du protocole de l’essai,
- les CV datés et signés des investigateurs du centre,
- la brochure de l'investigateur,
- les formulaires (originaux) complétés et signés de consentement éclairé pour chacun des patients inclus,
- l'avis du CPP,
- l'autorisation d'essai clinique donnée par l'AFSSAPS,
- l'attestation d'assurance souscrite par le Promoteur,
- le double du formulaire de mouvement des produits,
- les accusés de réception du matériel de l'essai,
- la liste en clair des patients inclus dans l'essai (nom, prénoms, n° du coffret de traitement et date d'inclusion, n° de dossier hospitalier si applicable),
- la liste des investigateurs participants,
- le cahier d'observation de chaque patient,
- les originaux des documents sources pour chacun des patients inclus,
- toute la correspondance relative à l'essai.

Ces documents doivent être conservés confidentiellement pendant un minimum de quinze ans après la fin de l'essai.

## Responsabilité et Assurances

Les patients, participant à l'essai, sont pris en charge par l'assurance du Promoteur souscrite pour cet essai, dans l'hypothèse où ils subiraient un dommage du fait de leur participation à l'essai.

Le Promoteur déclare avoir souscrit une police d'assurance garantissant les conséquences pécuniaires de sa responsabilité civile ainsi que celle de tout intervenant à la recherche et notamment celle des investigateurs et de leurs collaborateurs pour les accidents ou dommages relevant de l'administration du produit ou d'examens paracliniques directement liés à la réalisation de l'essai, sous réserve du respect des conditions légales définies par le Code de la Santé Publique (recherche effectuée sous la direction et la surveillance d'un médecin justifiant d'une expérience appropriée, recueil du consentement éclairé du patient, avis préalable du CPP, l'autorisation d'essai clinique donnée par l'AFSSAPS, lieu de recherche autorisé pour les recherches biomédicales, absence de décision d'interdiction ou de suspension de la recherche).

Cette garantie d'assurance est acquise pendant toute la durée de l'essai et pendant une période de 10 ans, à compter de la date de dernière remise d'unité de traitement.

L'investigateur doit vérifier qu'il est couvert par une assurance personnelle couvrant sa responsabilité civile pour tous dommages consécutifs à une faute de sa part (et notamment non respect du protocole) dans le cadre de cet essai.

## Confidentialité - Utilisation des Données et Publication des Résultats

Il est convenu que les résultats de cet essai ne peuvent être communiqués lors des réunions scientifiques ou publiés dans des revues scientifiques ou sur tout autre support qu'avec l'accord préalable et par écrit du Promoteur sur le contenu des résumés, articles, présentations visuelles ou orales. Ceci s’applique également à toute modification qui pourrait ensuite être demandée par un éditeur, un comité de lecture ou un comité de rédaction.

Les auteurs seront :

- l’investigateur coordonnateur qui sera premier ou dernier auteur
- l’investigateur ayant participé à la rédaction du premier projet s’il est différent de l’investigateur coordonnateur qui sera premier ou dernier auteur
- les investigateurs associés classés en fonction de leur niveau de participation à l’étude
- le statisticien ayant permis les hypothèses initiales et l’analyse des données s’il y a lieu

D’autres auteurs peuvent également être pressentis après accord des investigateurs.

Les auteurs conviendront, d’un commun accord, du choix de l’éditeur et de la revue de première publication auxquels ils accorderont les droits de première publication.

Le Promoteur se réserve le droit d’utiliser les résultats de cet essai dans son information médicale sur les médicaments, et de distribuer des tirés-à-part, des publications. Les auteurs cèdent au Promoteur leurs droits patrimoniaux d'auteur, de représentation et de reproduction, y compris de traduction en toutes langues, sur tous supports et par tous moyens, au niveau mondial, sur toutes leurs publications dans le cadre de l'essai.

Des tiers ne peuvent user de ces droits sans l’accord écrit préalable du Promoteur*.*

# ARRET PREMATURE DE L'ETUDE

Tout arrêt de l'étude sera justifié par écrit par le coordonnateur.

Cette lettre sera adressée à l'Agence Française de Sécurité Sanitaire et des Produits de Santé.

**ANNEXES**

##### Annexe 1 : Liste des investigateurs

Annexe 2 : Indice de performance

Annexe 3 : Toxicité NCI/CTCAE

Annexe 4 : Lettre d’information au patient

Annexe 5 : Consentement éclairé

Annexe 6 : Déclaration d’événement indésirable grave

Annexe 7: Critères de réponses OMS

Annexe 8 : Assurance

Annexe 9 : Avis du CPP

Annexe 10 : Autorisation d'essai clinique de l'AFSSAPS

# ANNEXE I : Liste des investigateurs

- **HOPITAL DE LA PITIE SALPETRIERE :** 75013 PARIS.

| **Investigateur principal**  Service d'Hépato-Gastro-Entérologie  Tél : 01.42.16.10.27/01  Fax : 01.42.16.14.25  47-83 Bd de l'Hôpital | Dr Touraj MANSOURBAKHT |
| --- | --- |
|  |  |

- **HOPITAL BICHAT-CLAUDE BERNARD :** 46 rue Henri Huchard, 75877 PARIS Cedex 18.

| **Investigateur principal**  Service d'Hépato-Gastro-Entérologie  Tél : 01 40 25 82 39 | Dr APARICIO |
| --- | --- |

- **HOPITAL EUROPEEN GEORGES POMPIDOU :** 20 rue Leblanc, 75908 PARIS Cedex 15.

| **Investigateur principal**  Service d'Hépato-Gastro-Entérologie  Tél : 01 56 09 35 51  Fax : 01 56 09 35 29 | Pr. Julien TAIEB (**Investigateur-Coordonnateur de l'étude**) |
| --- | --- |
| **Co-investigateurs de l'étude** | Dr Bruno LANDI  Dr Astrid LIEVRE  Dr Gabriel RAHMI |

- **HOPITAL D’INSTRUCTION des ARMEES du VAL DE GRACE** : 74 boulevard de Port Royal, 75230 PARIS Cedex 05

| **Investigateur principal**  Service d'Hépato-Gastro-Entérologie  Tél : 01.40.51.40.26 | Dr Jérome DESRAME |
| --- | --- |

- **CHU JEAN MINOZ :** 2 boulevard Fleming, 25030 BESANÇON Cedex.

| **Investigateur principal**  Service d'Hépato-Gastro-Entérologie  Tél : 03.81.66.86.09  Fax : 03.81.66.88.05 | Dr Anne-Claire DUPONT-GOSSARD |
| --- | --- |
| **Co-investigateurs de l'étude** | Dr Franck CARBONNEL  Dr Francine FEIN  Dr Maria NACHURY |

- **CLINIQUE ST JEAN : 30 rue bataille, 69008 LYON.**

| Investigateur principal  Service d'Hépato-Gastro-Entérologie  Tél : 04 78 78 10 51/10,  Fax : 04 78 74 07 92 | Dr Pascal ARTRU |
| --- | --- |
| **Co-investigateurs de l'étude** | Dr Gérard LLEDO |

- **POLYCLINIQUE JEANNE D'ARC :** 37 rue de la Marne, 45500 GIEN.

| **Investigateur principal**  Service d'Hépato-Gastro-Entérologie  Tél : 02 38 37 64 45  Fax : 02 38 37 64 87 | Dr David FALLIK |
| --- | --- |

**CHU de TOURS-Hôpital Trousseau :** Avenue de la République, 37170 CHAMBRAY-LES-TOURS.

| **Investigateur principal**  Service Gastro-Entérologie  Tél : 02 47 47 59 16  Fax : 02 47 47 84 28 | Dr Thierry LECOMTE |
| --- | --- |
| **Co-investigateurs de l'étude** | Dr Etienne DORVAL  Dr Philippe ASSOR  Dr Jérôme VIGUIER |

**Hôpital Antoine BECLERE :** 157, rue de la Porte de Trivaux 92141 CLAMART Cedex

| Investigateur principal  Service Gastro-Entérologie  Tél : 01 45 37 42 23  Fax : 01 45 37 40 44 | Dr Amani ASNACIOS |
| --- | --- |
| **Co-investigateurs de l'étude** | Pr Sylvie NAVEAU  Dr Barbara DAUVOIS  Dr Nadège BARRI-OVA  Dr Micheline NJIKE NAKSEU |

**Hôpital BICETRE**, 78, rue du Général Leclerc, 94275 LE KREMLIN-BICETRE

| Investigateur principal  Service Gastro-Entérologie  Tél : 01 45 21 28 40  Fax : 02 45 21 20 42 | Dr Anne THIROT-BIDAULT |
| --- | --- |

**Centre hospitalier René DUBOS:** 6 avenue de l’Ile de France, 95303 CERGY PONTOISE

| Investigateur principal  Service Gastro-Entérologie  Tél : 01 30 75 40 40/ poste 56 75  Fax : 01 30 75 44 31 | Dr Sophie MANET-LACOMBE |
| --- | --- |
| **Co-investigateurs de l'étude** |  |

# ANNEXE II : Indices de performance

| OMS | IK | Description |
| --- | --- | --- |
|  |  |  |
| 0 | 100 | Normal, ne se plaint de rien : pas de signe de la maladie |
|  | 90 | Capable d’une activité normale. Signes minimes de la maladie |
|  |  |  |
| 1 | 80 | Capable d’une activité normale avec effort. Quelques signes de la maladie |
|  |  |  |
| 2 | 70 | Prend soin de lui-même, mais est incapable d’une activité normale ou d’un travail |
|  |  |  |
| 3 | 60 | Requiert une assistance épisodique mais subvient à la plupart de ses besoins. |
|  | 50 | A besoin d’une assistance importante et de soins médicaux fréquents |
|  |  |  |
| 4 | 40 | Impotent. A besoin d’une assistance et de soins spéciaux |
|  | 30 | Sévèrement atteint. L’hospitalisation est souhaitable bien que la mort ne soit pas imminente |
|  | 20 | Gravement malade, hospitalisation et soins intensifs indispensables |
|  | 10 | Moribond |

# ANNEXE III : echelle de toxicite nci-ctcae (version 3)

| **Grade** | **0** | **1** | **2** | **3** | **4** |
| --- | --- | --- | --- | --- | --- |
| **Allergy** | | | | | |
| HS LER Allergy | none | transient rash, fever < 38°C, 100.4°F | urticaria, fever=38°C, 100.4°F, mild bronchospasm | serum sickness, bronchospasm, req. parenteral meds | anaphylaxis |
|  | Fever felt to be caused by drug allergy should be coded as ALLERGY (HS LER). Non-allergic drug fever (e.g. as from biologies should be coded under FLU-LIKE SYMPTOMS (FL FEV). If fever is due to infection, code INFECTION only (IN FEC or IN NEU). NB: Protocols requiring detailed reporting of hypersensitivity reactions, will include a Hypersensitivity Reaction module. | | | | |
| **Blood Bone Marrow (SI units)** | | | | | |
| BL WBC White Blood Count (WBC) | ≥4.0 10^6^/l | 3.0-3.9 | 2.0-2.9 | 1.0-1.9 | <1.0 |
| BL PLT Platelets | WNL 10^6^/l | 75.0-normal | 50.0-74.9 | 25.0-49.9 | <25.0 |
| BL HGB Hemoglobin(Hgb) | WNL g/L | 100-normal | 80-99 | 65-79 | <65 |
| BL GRA Granulocytes (i.e. neuts + bands) | ≥2.0 10^6^/l | 1.5-1.9 | 1.0-1.4 | 0.5-0.9 | <0.5 |
| BL LYM Lymphocytes | ≥2.0 10^6^/l | 1.5-1.9 | 1.0-1.4 | 0.5-0.9 | <0.5 |
| BL HEM Hemorrhage  resulting from thrombo-cytopenia (clinical) | none | mild, no transfusion (incl bruise/hematoma, petechiae) | gross, 1-2 units transfusion per episode | gross, 3-4 units transfusion per episode | massive, >4 units transfusion per episode |
| BL OTH Other* | none | mild | moderate | severe | life threatening |
| **Cancer Related Symptoms** | | | | | |
| CA DEA Death  malignant disease within 30 days of treatment* (grade=5) | - | - | - | - | - |
| CA PAI Cancer pain* | none | pain, but no treatment req. | pain controlled with non-opioids | pain controlled with opioids | uncontrollable pain |
| CA SEC Second malignancy* | none | - | - | present | - |
| CA OTH Other* | none | mild | moderate | severe | life threatening |
| **Cardiovascular** | | | | | |
| CD ART Arterial* (non myocardial) | none | - | - | transient events  (eg. transient ischemic attack) | permanent event (eg. cerebral vascular accident) |
| CD VEN Venous | none | superficial (excl IV site reactionàcode SK LTO) | deep vein thrombosis not req anticoagulant therapy | deep vein thrombosis req anticoagulant therapy | pulmonary embolism |
| CD DYS Dysrhythmias | none | asymptomatic, transient, req no therapy | recurrent or persistent, no therapy req | req treatment | req monitoring, or hypotension, or ventricular tachycardia, or fibriliation |
| CD EDE Edema* (eg. peripheral edema) | none | 1+ or dependent in evening only | 2+ or dependent throughout day | 3+ | 4+, generalized anasarca |
| CD FUN Function | none | asymptomatic, decline of resting ejection fraction of ≥ 10% but < 20% of baseline value | asymptomatic, decline of resting ejection fraction by >20% of baseline value | mild CHF, responsive to therapy | severe or refractory CHF |
| CD HBP Hypertension | none or no change | asymptomatic, transient increase by >20mm Hg (D) or to >150/100 if previously WNL. No trt req | recurrent or persistent increase by >20mm Hg (D) or to >150/100 if previously WNL. No trt req | req therapy | hypertensive crisis |
| CD LBP Hypotension | none or no change | changes req no therapy (incl transient orthostatic hypotension) | req fluid replacement or other therapy but no hospitalization | req therapy & hospitalization;  resolves within 48hrs of stopping agent | req therapy & hospitalization for >48hrs after stopping agent |

**ANNEXE III (suite 1) : ECHELLE DE TOXICITE NCI-CTCAE (VERSION 3)**

| **Grade** | **0** | **1** | **2** | **3** | **4** |
| --- | --- | --- | --- | --- | --- |
| CD ISC Ischemia (myocardial) | none | non-specific T wave flattening | asymptomatic, ST & T wave changes suggesting ischemia | angina without evidence for infarction | acute myocardial infarction |
| CD PAI Pain (chest)* | none | pain, but no treatment req | pain controlled with non-opioids | pain controlled with opioids | uncontrollable pain |
| CD PER Pericardial | none | asymptomatic, effusion, no intervention req | pericarditis (rub, chest pain, ECG changes) | symptomatic effusion; drainage req | tamponade, drainage urgently req; or constrictive pericarditis req surgery |
| CD TAC Sinus tachycardia* | none | mild | moderate | severe | life threatening |
| CD OTH Other* | none | mild | moderate | severe | life threatening |
| **Coagulation** | | | | | |
| CG FIB Fibrinogen | WNL | 0.99-0.75 x N | 0.74-0.50 x N | 0.49-0.25 x N | ≤0.4 x N |
| CG PT Prothrombin time | WNL | 1.01-1.25 x N | 1.26-1.50 x N | 1.51-2.00 x N | >2.00 x N |
| CG PTT Partial thrombo-plastin time | WNL | 1.01-1.66 x N | 1.67-2.33 x N | 2.34-3.00 x N | >3.00 x N |
| CG OTH Other* | none | mild | moderate | severe | life threatening |
| **Dentition (teeth)** | | | | | |
| DE DEC Tooth decay* | none | mild | moderate | severe | - |
| DE PAI Toothache* | none | pain, but no treatment req | pain controlled witn non-opioids | pain controlled with opioids | uncontrollable pain |
| DE OTH Other* | none | mild | moderate | severe | life threatening |
| **Endocrine** | | | | | |
| EN AME Amenorrhea | none | irregular menses | ≥3 mths | - | - |
| EN CUS Cushingoid | normal | mild | pronounced | - | - |
| EN FLA Hot flashes | none | mild or <1/day | moderate & ≥1/day | frequent & interferes with normal function | - |
| EN GYN Gyneco-mastia | normal | mild | pronounced or painful | - | - |
| EN IMP impotence / Libido | normal | decrease in normal function | - | absence of function | - |
| EN OTH Other | none | mild | moderate | severe | life threatening |
| **Flu-Like Symptoms** | | | | | |
| FL FEV Fever in absence of infection (incl drug fever) | none | 37.1-38.0°C 98.7-100.4°F | 38.1-40.0°C 100.5-104.0°F | >40.0°C >104.0°F for <24hrs | >40.0°C (104.0°F)  for >24hrs or fever accompanied by hypotension |
|  | Fever felt to be caused by drug allergy should be coded as ALLERGY (HS LER). Non-allergic drug fever (eg. as from biologics) should be coded under FLU-LIKE SYMPTOMS (FL FEV). If fever is due to infection, code INFECTION only (IN FEC or IN NEU). | | | | |
| FL HAY Hayfever* (incl sneezing, nasal stuffiness, post-nasal drip) | none | mild | moderate | severe | - |
| FL JOI Arthralgia* (joint pain) | none | mild | moderate | severe | - |
| FL LET Lethargy* (fatigue, malaise) | none | mild, or fall of 1 level in performance status | moderate, or fall of 2 levels in perf. status | severe, or fall of 3 levels in perf. status | - |
| FL MYA Myalgia*  (muscle ache) | none | mild | moderate | severe | - |
| FL RIG Rigors/Chills*(Gr 3 incl cyanosis) | none | mild or brief | pronounced and/or prolonged | cyanosis | - |

**ANNEXE III (suite 2) : ECHELLE DE TOXICITE NCI-CTCAE (VERSION 3)**

| **Grade** | **0** | **1** | | | **2** | **3** | **4** |
| --- | --- | --- | --- | --- | --- | --- | --- |
| **Gastrointestinal** | | | | | | | |
| FL SWE Sweating* (diaphoresis) | none | | mild | moderate | | severe | - |
| FL OTH Other* | none | | mild | moderate | | severe | life threatening |
| GI ANO Anorexia* | none | mild | | | moderate | severe | dehydration |
| GI APP Appetite-increased* | none | mild | | | moderate | - | - |
| GI ASC Ascites (non-malignant)* | none | mild | | | moderate | severe | life threatening |
| GI DIA Diarrhea | none | increase of 2-3 stools/ day; or mild increase in loose watery colostomy output compared to pre-trt | | | increase of 4-6 stools/ day, or nocturnal stools; or moderate increase in loose watery colostomy output compared to pre-trt | increase of 7-9 stools/ day, or incontinence, malabsorption; or severe increase in loose watery colostomy output compared to pre-trt | increase of ≥10 stools/ day or grossly bloody diarrhea; or grossly bloody colostomy output or loose watery colostomy output req parenteral support; dehydration |
| GI DPH Esophagitis/ dysphagia/ odynophagia*  (incl recall reaction) | none | dys. or odyn. not req trt, or painless ulcers on esophagoscopy | | | dys. or odyn. req trt | dys. or odyn. lasting >14 days despite trt | dys. or odyn. with 10% loss of body wt, dehydration, hosp. req |
| GI DRY Mouth, nose dryness* | none | mild | | | moderate | severe | - |
| GI FIS Fistula  (intestinal, esophageal, rectal)* | none | - | | | - | req intervention | req operation |
| GI GAS Flatulence* | none | mild | | | moderate | severe | - |
| GI HEA Heartburn* (incl dyspepsia) | none | mild | | | moderate | severe | - |
| GI HEM Gastrointestinal bleeding | none | mild, no transfusion | | | gross, 1-2 units transfusion per episode | gross, 3-4 units transfusion per episode | massive, >4 units transfusion per episode |
|  | Bleeding resulting from thrombocytopenia should be coded under BL HEM, not GI | | | | | | |
| GI NAU Nausea | none | able to eat reasonable intake | | | intake significantly decreased but can eat | no significant intake | - |
| GI OBS Small bowel obstruction* | none | - | | | intermittent, no intervention | req intervention | req operation |
| GI PAI Gastrointestinal pain/cramping* (incl rectal pain) | none | pain, but no treatment req | | | pain controlled with non-opioids | pain controlled with opioids | uncontrollable pain |
| GI PRO Proctitis (rectal) | none | perianal itch, hemorrhoids | | | tenesmus or ulcerations relieved with therapy, anal fissure | tenesmus or ulcerations or other symptoms not relieved with therapy | mucosal necrosis with hemorrhage or other life threatening proctitis |
| GI STO Stomatitis/oral | none | painless ulcers, erythema, or mild soreness | | | painful erythema, edema, or ulcers but can eat | painful erythema, edema, or ulcers, and cannot eat | mucosal necrosis and/or req parenteral or entral support, dehydration |
| GI TAS Taste, sense of smell altered* | none | mild | | | moderate | severe | - |
| GI ULC Gastritis/ulcer* | none | antacid | | | req vigorous medical management or non-surgical trt | uncontrolled by medical management; req surgery for GI ulceration | perforation or bleeding |
| GI VOM Vomiting | none | 1 episode in 24hrs | | | 2-5 episodes in 24hrs | 6-10 episodes in 24hrs | >10 episodes in 24hrs or req parenteral support, dehydration |
| GI OTH Other* | none | mild | | | moderate | severe | life threatening |

**ANNEXE III (suite 3) : ECHELLE DE TOXICITE NCI-CTCAE (VERSION 3)**

| **Grade** | **0** | **1** | **2** | **3** | **4** |
| --- | --- | --- | --- | --- | --- |
| **Genitourinary** | | | | | |
| GU BLA Bladder changes* | none | light epithelial atrophy, or minor telangiectasia | generalized telangiectasia | severe generalized telangiectasia (often with petechiae) or reduction in bladder capacity (<15ml) | necrosis, or contracted bladder (capacity <100ml), or fibrosis |
| GU CRE Creatinine | WNL | <1.5 x N | 1.5-3.0 X N | 3.1-6.0 x N | >6.0 x N |
| GU CYS Cystitis* (non-bacterial) | none | mild symptoms req no intervention | symptoms relieved completely with therapy | symptoms not relieved despite therapy | severe (life threatening) cystitis |
|  | Urinary tract infection should be coded under infection, not GU | | | | |
| GU FIS Fistula* (vaginal, vesicovaginal) | none | - | - | req intervention | req operation |
| GU FRE Frequency* | none | freq of urination or nocturia twice pre-trt habit | freq of urination or nocturia <hourly | frq with urgency and nocturia ≥hourly | - |
| GU HEM Hematuria, bleeding per vagina | neg | micro only | gross, no clots | gross + clots | req tranfusion |
|  | Bleeding resulting from thrombocytopenia should be coded under BL HEM not GU | | | | |
| GU INC Incontinence* | none | mild | moderate | severe | - |
| GU OBS Ureteral obstruction* | none | unilateral, no surgery | bilateral, no surgery req | no complete bilateral, but stents, nephrostomy tubes or surgery req | complete bilateral obstruction |
| GU PAI Genito-urinary pain* (eg dysuria, dysmenorrhea, dyspareunia) | none | pain, but no treatment req | pain controlled with non-opioids | pain controlled with opioids | uncontrollable pain |
| GU PRT Proteinuria | no change | 1+ or <0.3g% or <3g/L | 2-3+ or 0.3-1.0g% or 3-10g/L | 4+ or >1.0g% or >10g/L | nephrotic syndrome |
| GU VAG Vaginitis*  (+/- vaginal discharge) (non-infectious) | none | mild, no trt req | moderate, relieved with trt | severe, not relieved with trt | life threatening |
| GU OTH Other* | none | mild | moderate | severe | life threatening |
| **Hepatic** | | | | | |
| HP ALK Alk Phos or 5'nucleotidase | WNL | ≤2.5 x N | 2.6-5.0 x N | 5.-20.0 x N | >20.0 x N |
| HP ALT Transaminase SGPT (ALT) | WNL | ≤2.5 x N | 2.6-5.0 x N | 5.-20.0 x N | >20.0 x N |
| HP AST Transaminase SGOT (AST) | WNL | ≤2.5 x N | 2.6-5.0 x N | 5.-20.0 x N | >20.0 x N |
| HP BIL Bilirubin | WNL | - | <1.5 x N | 1.5-3.0 x N | >3.0 x N |
| HP CLI Liver (clinical) | no change from baseline | - | - | precoma | hepatic coma |
| HP LDH LDH* | WNL | <2.5 x N | 2.6-5.0 x N | 5.1-20.0 x N | >20.0 x N |
| HP OTH Other* | none | mild | moderate | severe | life threatening |
|  | Viral Hepatitis should be coded as infection rather than liver toxicity. | | | | |

**ANNEXE III (suite 4) : ECHELLE DE TOXICITE NCI-CTCAE (VERSION 3)**

| **Grade** | **0** | **1** | **2** | **3** | **4** |
| --- | --- | --- | --- | --- | --- |
| **Hypersensitivity** | | | | | |
| HS ANG Angio edema* | none | mild | moderate | severe | life threatening |
| HS BRO Bronchospasm* | none | mild | moderate | severe | life threatening |
| HS CDP Chest tightness* | none | mild | moderate | severe | life threatening |
| HS COU Cough* | none | mild | moderate | severe | life threatening |
| HS CYA Cyanosis (face, nails)* | none | mild | moderate | severe | life threatening |
| HS DIA Diarrhea* | none | mild | moderate | severe | life threatening |
| HS DIZ Dizziness, light-headed feeling* | none | mild | moderate | severe | life threatening |
| HS EDE Swelling* | none | mild | moderate | severe | life threatening |
| HS FEV Drug fever* | none | mild | moderate | severe | life threatening |
| HS FLU Flushing* | none | mild | moderate | severe | life threatening |
| HS GIP Abdominal pain* | none | mild | moderate | severe | life threatening |
| HS HBP Hypertension* | none | mild | moderate | severe | life threatening |
| HS LBP Hypotension* | none | mild | moderate | severe | life threatening |
| HS NAU Nausea* | none | mild | moderate | severe | life threatening |
| HS PAI Pain* | none | mild | moderate | severe | life threatening |
| HS PRU Pruritus* | none | mild | moderate | severe | life threatening |
| HS RAS Rash* | none | mild | moderate | severe | life threatening |
| HS RIG Chills, rigors* | none | mild | moderate | severe | life threatening |
| HS SOB Dyspnea | none | mild | moderate | severe | life threatening |
| HS SWE Sweating, diaphoresis* | none | mild | moderate | severe | life threatening |
| HS TAC Tacchycardia* | none | mild | moderate | severe | life threatening |
| HS TRE Tremors, shaking, jitteriness* | none | mild | moderate | severe | life threatening |
| HS URT Urticaria* | none | mild | moderate | severe | life threatening |
| HS VIS Visual disturbances* | none | mild | moderate | severe | life threatening |
| HS VOM Vomiting* | none | mild | moderate | severe | life threatening |
| HS OTH Other* | none | mild | moderate | severe | life threatening |
| **Infection** | | | | | |
| IN FEC Infection | none | mild, no active trt | moderate, localized infect req active trt | severe, systemic infect req parenteral trt, specify site | life threatening sepsis, specify site |
| IN NEU Febrile neutropenia*  Absolute gran. count <1.0x^106/l^, fever ≥38.5°C treated with (or ought to | none | - | - | present | - |
| have been treated with) IV antibiotics | Fever felt to be caused by drug allergy should be coded as ALLERGY (HS LER). Non-allergic drug fever (eg. as from biologics) should be coded under FLU-LIKE SYMPTOMS (FL FEV). If fever is due to infection, code INFECTION only (IN FEC or IN NEU). | | | | |

**ANNEXE III (suite 5) : ECHELLE DE TOXICITE NCI-CTCAE (VERSION 3)**

| **Grade** | **0** | **1** | **2** | **3** | **4** |
| --- | --- | --- | --- | --- | --- |
| **Metabolic (SI units)** | | | | | |
| MT AMY Amylase | WNL | <1.5 x N | 1.5-2.0 x N | 2.1-5.0 x N | >5.1 x N |
| MT HCA Hypercalcemia | <2.64 mmol/L | 2.64-2.88 | 2.89-3.12 | 3.13-3.37 | ≥3.37 |
| MT LCA Hypocalcemia | >2.10 mmol/L | 2.10-1.93 | 1.92-1.74 | 1.73-1.51 | ≤1.50 |
| MT HGL Hyperglycemia | <6.44 mmol/L | 6.44-8.90 | 8.91-13.8 | 13.9-27.8 | >27.8 or ketoacidosis |
| MT LGL Hypoglycemia | >3.55 mmol/L | 3.03-3.55 | 2.19-3.02 | 1.66-2.18 | <1.66 |
| MT LKA Hypokalemia* | no change or >3.5 mmol/L | 3.1-3.5 | 2.6-3.0 | 2.1-2.5 | ≤2.0 |
| MT LMA Hypomagne- semia | >0.70 mmol/L | 0.70-0.58 | 0.57-0.38 | 0.37-0.30 | ≤0.29 |
| MT LNA Hyponatre- mia* | no change or >135 mmol/L | 131-135 | 126-130 | 121-125 | ≤120 |
| MT OTH Other* | none | mild | moderate | severe | life threatening |
| **Neurologic** | | | | | |
| NE CER Cerebellar | none | slight incoordination, dysdiadochokinesis | intention tremor, dysmetria, slurred speech, nystagmus | locomotor ataxia | cerebellar necrosis |
| NE CON Constipation | none or no change | mild | moderate | severe, obstipation | ileus >96hrs |
| NE COR Cortical, somnolence  (incl drowsiness) | none | mild somnolence | moderate somnolence | severe somnolence, confusion, disorientation, hallucinations | coma, seizures, toxic psychosis |
| NE DIZ Dizziness* (incl light headedness) | none | mild | moderate | severe (incl fainting) | - |
| NE EXT Extrapyrami- -dal / Involuntary movement* | none | mild agitation (incl restlessness) | moderate agitation | torticollis, oculogyric crisis, severe agitation | - |
| NE HED Headache | none | mild | moderate or severe but transient | unrelenting & severe | - |
| NE HER Altered hearing | none or no change | asymptomatic, hearing changes on audiometry only | tinnitus, symptomatic hearing changes not req hearing aid or trt | hearing changes interfering with function but correctable with hearing aid or trt | hearing changes or deafness not correctable |
| NE INS Insomnia* | none | mild | moderate | severe | - |
| NE MOO Mood | no change | mild anxiety or depression | moderate anxiety or depression | severe anxiety or depression | suicidal ideation |
| NE MOT Motor | none or no change | subjective weakness; no objective findings | mild objective weakness without significant impairment of function | objective weakness with impairment of function | paralysis |
| NE PAI Neurologic  pain* (eg. jaw pain) | none | pain but no treatment req | pain controlled with non-opioids | pain controlled with opioids | uncontrollable pain |
| NE PER Personality change* | no change | change, not disruptive to pt or family | disruptive to pt or family | harmful to others or self | psychosis |
| NE SEN Sensory | none or no change | mild paresthesias, loss of deep tendon reflexes (incl tingling) | mild or moderate objective sensory loss; moderate paresthesias | sensory loss or paresthesias that interfere with function | - |
| NE VIS Vision | none or no change | blurred vision | - | symptomatic subtotal loss of vision | blindness |
| NE OTH Other* | none | mild | moderate | severe | life threatening |

**ANNEXE III (suite 6) : ECHELLE DE TOXICITE NCI-CTCAE (VERSION 3)**

| **Grade** | **0** | **1** | **2** | **3** | **4** |
| --- | --- | --- | --- | --- | --- |
| **Ocular** | | | | | |
| OC CAT Cataract* | none | mild | moderate | severe | - |
| OC CJN Conjunctivitis/ Keratitis | none | erythema or chemosis not req steroids or antibiotics | req trt with steroids or antibiotics | corneal ulceration or visible opacification | - |
| OC DRY Dry eye | normal | mild | req artificial tears | severe | req enucleation |
| OC GLA Glaucoma | no change | - | - | yes | - |
| OC PAI Eye pain* | none | pain, but no treatment req | pain controlled with non-opioids | pain controlled with opioids | uncontrollable pain |
| OC TEA Tearing* (watery eyes) | none | mild | moderate | severe | - |
| OC OTH Other | none | mild | moderate | severe | life threatening |
| **Osseous (bone)** | | | | | |
| OS PAI Bone pain* | none | pain, but no treatment req | pain controlled with non-opioids | pain controlled with opioids | uncontrollable pain |
| OS OTH Other*  (eg. avascular necrosis) | none | mild | moderate | severe | life threatening |
| **Other** | | | | | |
| OT OTH Other | none | mild | moderate | severe | life threatening |
|  | For toxicities which do not have an existing code, but do fit into an existing toxicity category, use "other" variable in the appro-priate toxicity category. Only toxicities which do not fit into existing categories should be coded OTHER OTHER (OT OTH). | | | | |
| **Pulmonary** | | | | | |
| PU CMD Carbon Monoxide Diffusion Capacity (DLCO)* | >90% of pretreatment value | decrease to 76-90% of pre-trt | decrease to 51-75% of pre-trt | decrease to 26-50% of pre-trt | decrease to ≤25% of pre-trt |
| PU COU Cough* | none | mild | moderate | severe | - |
| PU EDE Pulmonary Edema* | none | - | out-pt management | in-pt management | req intubation |
| PU EFF Pleural effusion* (non-malignant) | none | mild | moderate | severe | life threatening |
| PU FIB Pulmonary fibrosis* | normal | radiographie changes, no symptoms | - | changes with symptoms | - |
| PU HEM Hemophtysis* | none | mild, no transfusion | gross, 1-2 units transfusion per episode | gross, 3-4 units transfusion per episode | massive, >4 units transfusion per episode |
|  | Bleeding resulting from thrombocytopenia should be coded under BL HEM, not PU | | | | |
| PU HIC Hiccough | none | mild | moderate | severe | - |
| PU PAI Pulmonary pain* | none | pain, but no treatment req | pain controlled with non-opioids | pain controlled with opioids | uncontrollable pain |
| PU PNE Pneumonitis* (non infectious) | normal | radiographic changes, symptoms do not req steroids | steroids req | oxygen req | req assisted ventilation |
| PU SOB Shortness of breath (SOB) (last wheezing) | none or no change | asymptomatic, with abnormally to PFT's | dyspnea on significant exertion | dyspnea at normal level of activity apnea without cyanosis | dyspnea at rest, apnea with cyanosis |
| PU VOI Voice  changes* (incl hoarse-ness, loss of voice) | none | mild | moderate | severe | - |
| PU OTH Other* | none | mild | moderate | severe | life threatening |
|  | Pneumonia is considered infection and not graded as pulmonary toxicity unless felt to be resultant from pulmonary changes directly induced by treatment. | | | | |

**ANNEXE III (suite 7) : ECHELLE DE TOXICITE NCI-CTCAE (VERSION 3)**

| **Grade** | **0** | **1** | **2** | **3** | **4** |
| --- | --- | --- | --- | --- | --- |
| **Skin** | | | | | |
| SK ALO Alopecia | no loss | mild hair loss | pronounced or total head hair loss | total body hair loss | - |
| SK CHA Skin changes* (eg. photosensitivity) | none | localized pigmentation changes | generalized pigmenta-tion changes or atrophy | subcut fibrosis or localized shallow ulceration | generalized ulcerations or necrosis |
| SK DES Desquamation* | none | dry desquamation | moist desquamation | confluent moist desquamation | - |
| SK DRY Dry skin | none | mild | moderate | severe | - |
| SK FAC Flushing*  (eg. facial) | none | mild | moderate | severe | - |
| SK HEM Bruising/bleeding | none | mild, no transfusion | gross, 1-2 units transfusion per episode | gross, 3-4 units tranfusion per episode | massive, >4 units transfusion per episode |
|  | Bleeding resulting from thrombocytopenia should be coded under BL HEM, not SK. | | | | |
| SK LTO Local Toxicity (reaction at IV site) | none | pain | pain & swelling, with inflammation or phlebitis | ulceration | plastic surgery indicated |
| SK NAI Nail changes* | none | mild | moderate | severe | - |
| SK PAI Skin pain* (incl scalp pain) | none | pain, but no treatment req | pain controlled with non-opioids | pain controlled with opioids | uncontrollable pain |
| SK RAS Rash/itch* (not due to allergy) (incl recall reaction) | none or no change | scattered macular or papular eruption or erythema that is asymptomatic | scattered macular or papular eruption or erythema with pruritus or other associated symptoms | generalized asymptomatic macular, papular or vesicular eruption | exfoliative dermatitis or ulcerating dermatitis |
| SK OTH Other* | none | mild | moderate | severe | life threatening |
| **Weight** | | | | | |
| WT GAI Weight gain | <5.0% | 5.0-9.9% | 10.0-19.9% | ≥20.0% | - |
| WT LOS Weight loss | <5.0% | 5.0-9.9% | 10.0-19.9% | ≥20.0% | - |

# ANNEXE IV : Resumes des caractéristiques des produits

### IV-1 Irinotécan

RÉSUMÉ DES CARACTÉRISTIQUES DU PRODUIT

Mis à jour : 21/11/2006

1. DÉNOMINATION DU MÉDICAMENT

CAMPTO 100 mg/5 ml, solution à diluer pour perfusion (IV)

2. COMPOSITION QUALITATIVE ET QUANTITATIVE

Irinotecan....................................................................................................................................... 17,33 mg

(Sous forme de chlorhydrate d'irinotecan trihydraté............................................................................. 20,00 mg)

pour 1 ml de solution

Un flacon de 5 ml contient 100 mg de chlorhydrate d'irinotecan trihydraté.

Pour les excipients, voir rubrique 6.1

3. FORME PHARMACEUTIQUE

Solution à diluer pour perfusion.

4. DONNÉES CLINIQUES

4.1       Indications thérapeutiques

CAMPTO est indiqué dans le traitement des cancers colorectaux avancés:

• en association avec le 5-fluorouracile (5FU)et l'acide folinique (AF) chez les patients n'ayant pas reçu de chimiothérapie antérieure pour le stade avancé de leur maladie,

• en monothérapie après échec d'un traitement ayant comporté du 5-FU.

CAMPTO en association avec le cetuximab est indiqué dans le traitement des patients présentant un cancer colorectal métastatique exprimant lerécepteur dufacteur de croissance épidermique (EGFR) après échec d’une chimiothérapie à base d’irinotecan.

4.2       Posologie et mode d'administration

Réservé à l'adulte. La solution de CAMPTO doit être perfusée dans une veine périphérique ou centrale.

Posologie recommandée

En monothérapie (chez les patients prétraités) :

La posologie recommandée est de 350 mg/m² de CAMPTO administrés en perfusion intraveineuse de 30 à 90 minutes toutes les 3 semaines (cf. "Mode d'emploi et instructions concernant la manipulation" et "Mises en garde spéciales et précautions particulières d'emploi").

En association (chez les patients non prétraités) :

L'efficacité et la tolérance de CAMPTO en association avec le 5-fluorouracile (5-FU) et l'acide folinique (AF) ont été évaluées selon le schéma suivant (cf. "Propriétés pharmacodynamiques") :

CAMPTO et 5-FU/AF : schéma toutes les 2 semaines.

La dose recommandée est de 180 mg/m² de CAMPTO en perfusion intraveineuse de 30 à 90 minutes toutes les 2 semaines, suivie d'une perfusion d'acide folinique et de 5-FU.

Concernant la posologie et le mode d’administration de l’irinotecan administré en association avec le cetuximab, se référer au Résumé des Caractéristiques du Produit de ce médicament. La dose d’irinotecan utilisée est généralement la même que celle administrée au cours des derniers cycles du précédent traitement à base d’irinotecan. Irinotecan doit être administré au moins une heure après la fin de la perfusion du cetuximab.

Ajustements posologiques

CAMPTO doit être administré après récupération convenable de tous les effets indésirables, c'est-à-dire grade 0 ou 1 selon les critères NCI-CTC (National Cancer Institute Common Toxicity Criteria) et après disparition complète d'une diarrhée liée au traitement.

Avant chaque administration du traitement, les doses de CAMPTO et de 5-FU si nécessaire, devront être réduites, en tenant compte des effets indésirables de plus haut grade observés et liés à l'administration précédente. Le traitement doit être retardé d'une ou deux semaines afin de permettre une récupération de tous les effets indésirables liés au traitement.

La posologie de CAMPTO et/ou de 5-FU si nécessaire, devra être réduite de 15 à 20% en cas de survenue des effets indésirables suivants :

• toxicité hématologique : neutropénie grade 4, neutropénie fébrile (neutropénie grade 3-4 et fièvre grade 2-4), thrombopénie et leucopénie (grade 4),

• toxicité non hématologique (grade 3- 4).

Les recommandations de modification de doses du cetuximab, administré en association avec l’ironetecan, doivent être en accord avec le Résumé des Caractéristiques du Produit du cetuximab.

Durée du traitement :

Le traitement par CAMPTO doit être poursuivi jusqu'à progression objective de la maladie ou apparition d'une toxicité inacceptable.

Populations à risque :

• Patients présentant une insuffisance hépatique :

     En monothérapie : la bilirubinémie (jusqu'à 3 fois la limite supérieure de la normale (LSN)), chez les patients ayant un indice de performance ≤ 2, conditionne la posologie initiale de CAMPTO

     Chez ces patients ayant une hyperbilirubinémie et un taux de prothrombine supérieur à 50%, la clairance d'irinotecan est diminuée (voir "Propriétés pharmacocinétiques") et le risque de toxicité hématopoïétique est donc augmenté.

**Une surveillance hebdomadaire de la numération et de la formule sanguine doit alors être réalisée.**

• Pour les patients ayant une bilirubinémie ≤ 1,5 fois la LSN, la dose recommandée de CAMPTO est de 350 mg/m^2^.

• Pour les patients ayant une bilirubinémie comprise entre 1,5 et 3 fois la LSN, la dose recommandée de CAMPTO est de 200 mg/m^2^.

• Pour les patients ayant une bilirubinémie > 3 fois la LSN, CAMPTO est contre-indiqué. (cf. "Contre-indications" et "Mises en garde spéciales et précautions particulières d'emploi").

Il n'y a pas de donnée disponible chez les patients présentant une insuffisance hépatique traités par CAMPTO en association.

• Patients présentant une insuffisance rénale :

     Aucune étude spécifique n'ayant été réalisée chez les insuffisants rénaux, CAMPTO n'est pas recommandé chez les patients présentant une insuffisance rénale (voir "Mises en garde spéciales et précautions particulières d'emploi" et "Propriétés pharmacocinétiques").

• Sujets âgés :

     Aucune étude pharmacocinétique spécifique n'a été réalisée chez le sujet âgé.

     Toutefois, la dose doit être choisie avec prudence dans cette population en raison de la fréquence plus importante de l'altération des fonctions biologiques. Cette population demande une surveillance plus étroite.

     (voir "Mises en garde spéciales et précautions particulières d'emploi").

4.3       Contre-indications

• Maladie inflammatoire chronique de l'intestin et/ou avec une occlusion intestinale. (cf. "Mises en garde spéciales et précautions particulières d'emploi").

• Antécédents d'hypersensibilité sévère au chlorhydrate d'irinotecan trihydraté ou à l'un des excipients du CAMPTO.

• Grossesse et allaitement (cf. "Grossesse et allaitement" et "Mises en garde spéciales et précautions particulières d'emploi").

• Bilirubinémie > 3 fois la limite supérieure normale (LSN) (voir "Mises en garde spéciales et précautions particulières d'emploi").

• Insuffisance médullaire sévère.

• Indice de performance de grade OMS > 2.

• Association avec le millepertuis (voir « Interactions avec d'autres médicaments et autres formes d'interactions »).

Pour les contre-indications d’utilisation de cetuximab, se référer au résumé des caractéristiques du produit de ce médicament.

4.4       Mises en garde spéciales et précautions particulières d'emploi

| L'utilisation de CAMPTO doit être réservée aux unités spécialisées dans l'administration de cytotoxiques et doit être uniquement administré sous le contrôle d'un médecin qualifié dans l'utilisation des chimiothérapies anticancéreuses. |
| --- |

Etant donné la nature et la fréquence de survenue des effets indésirables, l'utilisation de CAMPTO ne sera décidée qu'après avoir pesé les avantages attendus par rapport aux éventuels risques thérapeutiques dans les cas suivants :

• chez les patients présentant un facteur de risque notamment un indice de performance de grade OMS = 2 (ou un indice de KARNOFSKY < 50) ;

• dans les rares cas, où il est prévisible que le patient n'observera pas les recommandations de prise en charge des effets indésirables (nécessité d'un traitement antidiarrhéique immédiat et suffisamment prolongé et de la prise abondante de liquide dès qu'une diarrhée tardive apparaît). Un suivi strict du patient à l'hôpital est recommandé.

Lorsque CAMPTO est utilisé en monothérapie, il est habituellement prescrit selon un schéma d'administration toutes les 3 semaines. Cependant, un schéma d'administration hebdomadaire (voir "Propriétés pharmacologiques") peut être envisagé chez des patients nécessitant un suivi plus rapproché ou qui ont un risque particulier de neutropénie sévère.

Diarrhée tardive :

Les patients doivent être avertis du risque de diarrhée tardive survenant plus de 24 heures après l'administration de CAMPTO à tout moment de l'intercure.

En monothérapie, le délai médian d'apparition de la première selle liquide est de 5 jours après la perfusion de CAMPTO. En cas d'apparition de cet effet indésirable, les patients doivent prévenir rapidement leur médecin et débuter immédiatement un traitement adapté.

Le risque de diarrhée est augmenté chez les patients ayant reçu une radiothérapie abdomino-pelvienne, chez les patients ayant une hyperleucocytose initiale et ceux avec un indice de performance de grade OMS≥2 et chez les femmes. Si la diarrhée n'est pas traitée correctement, elle peut menacer le pronostic vital, particulièrement en cas de neutropénie concomitante.

Dès la première selle liquide, le patient doit boire abondamment des boissons riches en électrolytes et doit débuter immédiatement un traitement antidiarrhéique approprié. A cet effet, l'ordonnance de ce traitement antidiarrhéique sera établie dans le service où CAMPTO a été administré. A sa sortie de l’hôpital, le patient devra se procurer le médicament prescrit afin de pouvoir traiter la diarrhée dès sa survenue. De plus, le patient doit informer le médecin ou le service ayant administré CAMPTO de l’apparition de la diarrhée tardive.

Le traitement antidiarrhéique actuellement recommandé consiste en de fortes doses de lopéramide (~~4~~ mg lors de la première prise puis 2 mg toutes les 2 heures). Ce traitement doit être poursuivi au moins pendant 12 heures après la dernière selle liquide et sans modification de posologie. En aucun cas, le lopéramide ne doit être administré, à cette posologie, plus de 48heures consécutives, en raison du risque d'ileus paralytique.

Une antibiothérapie prophylactique à large spectre peut être associée au traitement antidiarrhéique dans les cas de diarrhée concomitante à une neutropénie sévère (nombre de neutrophiles < 500/mm^3^).

Une hospitalisation, associée à une antibiothérapie, est recommandée dans les cas suivants afin de contrôler la diarrhée :

• diarrhée accompagnée de fièvre,

• diarrhée sévère (demandant une réhydratation parentérale),

• diarrhée persistante après 48 heures de traitement à forte dose de lopéramide.

Le lopéramide ne doit pas être administré à titre prophylactique, même chez des patients ayant présenté une diarrhée tardive lors de cures précédentes.

Chez les patients ayant présenté une diarrhée tardive sévère, une réduction de la posologie est recommandée pour les cycles suivants (cf. "Posologie et mode d'administration").

Hématologie

Une surveillance hebdomadaire de l'hémogramme est recommandée pendant le traitement par CAMPTO. Les patients doivent être avertis du risque de neutropénie et de l'importance de la présence d'une fièvre. Une neutropénie fébrile (température >38°C et nombre de neutrophiles <1000/mm^3^) doit être traitée en urgence en milieu hospitalier, par des antibiotiques à large spectre par voie intraveineuse.

Chez les patients ayant eu une toxicité hématologique sévère, une réduction de dose est recommandée pour les administrations suivantes (cf. «Posologie et mode d’administration»).

Chez les patients ayant une diarrhée sévère, le risque d'infection et de toxicité hématologique est augmenté. Un hémogramme doit être réalisé en cas de diarrhée sévère.

Insuffisance hépatique

Avant la première administration de CAMPTO et avant chaque cycle, il est nécessaire de réaliser un bilan hépatique.

Une surveillance hebdomadaire de la numération sanguine doit être réalisée chez les patients ayant une bilirubinémie comprise entre 1,5 et 3 fois la LSN, en raison de la diminution de la clairance de l’irinotecan (voir "Propriétés pharmacocinétiques") et de l’augmentation du risque d’hématotoxicité.

CAMPTO ne doit pas être administré aux patients ayant une bilirubinémie > 3 fois la LSN (voir "Contre-indications").

Nausées et vomissements

Un traitement antiémétique prophylactique est recommandé avant chaque administration de CAMPTO. Des nausées et vomissements ont été fréquemment rapportés.

Les patients chez lesquels des vomissements sont associés à la diarrhée tardive, doivent être hospitalisés le plus rapidement possible.

Syndrome cholinergique aigu

Si un syndrome cholinergique aigu apparaît (défini par une diarrhée précoce et un ensemble de symptômes tels que hypersudation, crampes abdominales, larmoiements, myosis, et hypersalivation) du sulfate d'atropine (0,25 mg par voie sous-cutanée) doit être administré en dehors de ses contre-indications cliniques (voir "Effets indésirables"). Des précautions doivent être prises chez les patients asthmatiques.

Lorsqu'un syndrome cholinergique aigu a été observé lors de la première administration, l'utilisation de sulfate d'atropine à titre prophylactique est recommandée lors des administrations ultérieures de CAMPTO.

Troubles respiratoires

Les cas de pathologie pulmonaire interstitielle avec infiltrats pulmonaires sont peu fréquents sous CAMPTO. La pathologie pulmonaire interstitielle peut être fatale. Le risque de développement de pathologie pulmonaire interstitielle peut être favorisé par l’administration concomitante de médicaments pneumotoxiques, la radiothérapie, et l’administration de facteurs de croissance. Chez les patients présentant des facteurs de risques, la survenue de symptômes respiratoires devra être étroitement surveillée avant et pendant le traitement par CAMPTO.

Personnes âgées

En raison de la fréquence plus élevée de l'altération des fonctions biologiques en particulier de la fonction hépatique chez les personnes âgées, le choix de la posologie doit être fait avec précaution dans cette population (voir "Posologie et mode d'administration").

Patients avec occlusion intestinale

Ces patients ne doivent pas être traités par CAMPTO avant la résolution de l'occlusion intestinale (voir "Contre-indications").

Patients avec fonction rénale altérée

Aucune étude dans cette population n'a été faite (voir "Posologie et Mode d'Administration" et "Propriétés Pharmacocinétiques").

Autres

Ce médicament contenant du sorbitol, il n’est pas conseillé en cas d’intolérance héréditaire au fructose. De rares cas d’insuffisance rénale, d’hypotension ou d’hypovolémie ont été observés chez des patients ayant présenté des épisodes de déshydratation associés à une diarrhée et/ou à des vomissements, ou à une infection. Des mesures contraceptives doivent être prises pendant le traitement et poursuivies au moins trois mois après l'arrêt du traitement.

L’irinotecan ne doit pas être administré de façon concomitante à de puissants inhibiteurs du cytochrome CYP450 3A4 (comme le kétoconazole) ou inducteurs (comme la rifampicine, la carbamazépine, le phénobarbital, la phénytoïne ou le millepertuis) (voir "Interactions avec d'autres médicaments et autres formes d'interactions").

4.5       Interactions avec d’autres médicaments et autres formes d’interactionss

L'interaction entre irinotecan et les agents bloquants neuromusculaires ne peut pas être établie. Du fait de l’activité anticholinestérase de CAMPTO, les médicaments avec une activité anticholinestérase peuvent prolonger les effets bloquants neuromusculaires du suxaméthonium et le blocage neuromusculaire d'agents non-dépolarisants peut être antagonisé.

Plusieurs études ont montré que l’administration concomitante d’anti-convulsivants inducteurs du cytochrome CYP450 3A4 (comme la carbamazépine, le phénobarbital ou la phénytoïne) entraînait une diminution de l’exposition à l’irinotécan, au SN-38 ainsi qu’au SN38 glucuronidé et réduisait leurs effets pharmacodynamiques. Les effets de ces anti-convulsivants se traduisent par une diminution de 50% ou plus des aires sous la courbe (AUC) du SN-38 et du SN-38 glucuronidé. En plus de l’induction des enzymes du cytochrome CYP450 3A4, l’augmentation de la glucuronidation et de l’excrétion biliaire peut jouer un rôle sur la réduction de l’exposition à l’irinotecan et à ses métabolites.

Une étude a montré que la co-administration de l’irinotécan avec le kétoconazole comparée à l’irinotécan seul a entraîné une diminution de 87% de l’AUC de l’APC et à une élévation de 109% de l’AUC du SN-38.

Une attention particulière est requise chez les patients recevant de façon simultanée des médicaments connus pour inhiber (comme le kétoconazole) ou induire ( comme la rifampicine, la carbamazépine, le phénobarbital ou la phénytoïne) le métabolisme du cytochrome CYP450 3A4. L'administration concomitante d'irinotécan avec un inducteur ou inhibiteur de cette voie métabolique peut altérer le métabolisme de l'irinotécan et doit être évitée (voir "Mises en gardes spéciales et précautions d'emploi").

Dans une étude pharmacocinétique avec un faible nombre de patients (n=5) dans laquelle l’irinotécan a été administré à la dose de 350 mg/m² avec 900 mg de millepertuis *(Hypericum perforatum)*, une baisse de 42% des concentrations plasmatiques du SN-38, métabolite actif de l’irinotécan, a été observée.

Le millepertuis diminue les taux plasmatiques du SN-38. Le millepertuis ne doit donc pas être administré avec l’irinotécan (voir "Contre-indications").

L’administration concomitante de 5-fluorouracile et d'acide folinique dans le schéma d’association ne modifie pas les paramètres pharmacocinétiques de l'irinotecan.

L’influence de l’irinotecan sur sur le profil de tolérance du cetuximab, et vice versa, n’a pas été mis en évidence.

4.6       Grossesse et allaitement

Grossesse

Il n'y a aucune information concernant l'utilisation de CAMPTO chez la femme enceinte. Chez le lapin et le rat, des effets embryotoxiques, foetotoxiques et tératogènes ont été observés. Aussi, CAMPTO ne doit pas être utilisé pendant la grossesse (voir "Contre-indications" et "Mises en garde spéciales et précautions particulières d'emploi").

Femmes en âge de procréer :

Les femmes en âge de procréer recevant du CAMPTO doivent éviter toute grossesse et informer immédiatement leur médecin si elles venaient à être enceintes (voir "Contre-indications" et "Mises en garde spéciales et précautions particulières d'emploi").

Allaitement

Chez les rats qui allaitent, l'irinotecan marqué au C^14^ a été détecté dans le lait. Il n'existe pas de données relatives au passage de CAMPTO dans le lait maternel humain. En conséquence, en raison du risque potentiel de manifestations indésirables chez le nourrisson, l'allaitement doit être interrompu pendant la durée du traitement par CAMPTO (voir "Contre-indications").

4.7       Effets sur l'aptitude à conduire des véhicules et à utiliser des machines

Les patients doivent être avertis du risque potentiel de vertiges ou de troubles visuels pouvant se produire dans les 24 heures après l'administration du produit, et doivent éviter la conduite de véhicule ou l'utilisation d'une machine si de tels symptômes apparaissent.

4.8       Effets indésirables

Les effets indésirables détaillés dans ce paragraphe concernent l’irinotecan.

Aucune influence du cetuximab sur le profil de tolérance de l’irinotecan, et vice versa n’a été mise en évidence. En association avec le cetuximab, d’autres effets indésirables ont été rapportés et étaient ceux attendus avec le cetuximab (comme éruption acnéïforme chez 88% des patients). Par conséquent, se référer aussi au Résumé des Caractéristiques du Produit du cetuximab.

Les effets indésirables suivants possiblement ou probablement liés à l'administration de CAMPTO ont été analysés sur une population de 765 patients à la dose recommandée de 350mg/m² en monothérapie, et de 145 patients traités par CAMPTO en association avec 5-FU/ AF toutes les 2 semaines à la dose recommandée de 180 mg/m².

**TROUBLES GASTRO-INTESTINAUX**

*Diarrhée tardive :*

La diarrhée tardive (survenant plus de 24 heures après l'administration de CAMPTO) constitue une toxicité dose-limitante de CAMPTO.

En monothérapie:

Une diarrhée sévère est observée chez 20% des patients qui ont suivi les recommandations de prise en charge de la diarrhée. Une diarrhée sévère est retrouvée dans 14% des cycles évaluables.

Le délai médian d'apparition de la première selle liquide est de 5 jours après la perfusion de CAMPTO.

En association:

Une diarrhée sévère est observée chez 13,1% des patients qui ont suivi les recommandations de prise en charge de la diarrhée. Une diarrhée sévère est retrouvée dans 3,9% des cycles évaluables.

De rares cas de colite pseudomembraneuse ont été rapportés dont un cas avec une documentation bactériologique (*Clostridium difficile*).

*Nausées et vomissements :*

En monothérapie:

Des nausées et vomissements sévères sont observés chez environ 10 % des patients ayant reçu un traitement antiémétique.

En association:

Une incidence moindre des nausées et vomissements sévères est observée (respectivement 2,1% et 2,8% des patients)

*Déshydratation:*

Des épisodes de déshydratation généralement associés à une diarrhée et/ou des vomissements sont rapportés.

De rares cas d’insuffisance rénale, d’hypotension ou de collapsus cardio-vasculaire ont été observés chez des patients ayant présenté des épisodes de déshydratation associés à une diarrhée et/ou à des vomissements

*Autres événements gastro-intestinaux :*

Une constipation relative à CAMPTO et/ ou au lopéramide est observée:

• en monothérapie: chez moins de 10 % des patients

• en association: chez 3,4% des patients traités

De rares cas d'occlusion intestinale, d'iléus ou d’hémorragie gastrointestinale et de rares cas de colites, incluant des typhlites, des colites ischémiques et ulcéreuses ont été rapportés. De rares cas de perforations intestinales sont rapportés.

D'autres effets peu sévères incluant anorexie, douleurs abdominales et stomatite ont été également observés.

De rares cas de pancréatite symptomatique ou asymptomatique liés au traitement par irinotécan, ont été observés.

**ATTEINTES HEMATOLOGIQUES**

La neutropénie constitue une toxicité dose limitante. La neutropénie était réversible et non cumulative; le délai médian d'apparition du nadir était de 8 jours que ce soit en monothérapie ou en association.

En monothérapie:

La neutropénie est observée chez 78,7 % des patients et est sévère (nombre de neutrophiles <500/mm^3^) dans 22,6 % des cas. Parmi les cycles évaluables, 18% se compliquent d’une neutropénie <1000/mm^3^ dont 7,6 % d’une neutropénie <500/mm^3^.

La récupération totale est en général atteinte le 22^ème^ jour.

Une fièvre accompagnée d’une neutropénie sévère est rapportée chez 6,2 % des patients et dans 1,7% des cycles.

***Des épisodes infectieux sont survenus chez environ 10,3 % des patients (2,5 % des cycles), ont été associés à une neutropénie sévère chez environ 5,3 % des patients (1,1% des cycles), et ont entraîné 2 décès.***

Une anémie a été rapportée chez environ 58,7 % des patients (8 % avec une hémoglobine < 8 g/dl et 0,9 % avec une hémoglobine < 6,5 g/dl).

Une thrombocytopénie (< 100000/mm^3^) a été observée chez 7,4 % des patients et 1,8% des cycles dont 0,9 % des patients avec des plaquettes ≤ 50000 /mm^3^, soit 0,2% des cycles. Presque tous les patients ont normalisé leur numération plaquettaire le 22ème jour.

En association:

Une neutropénie est observée chez 82,5 % des patients et est sévère (nombre de neutrophiles <500/mm^3^) dans 9,8 % des cas. Parmi les cycles évaluables, 67,3% se compliquent d’une neutropénie <1000/mm^3^ dont 2,7% d’une neutropénie <500/mm^3^. La récupération totale est en général atteinte dans les 7 à 8 jours.

Une fièvre accompagnée d’une neutropénie sévère est rapportée chez 3,4 % des patients et dans 0,9% des cycles.

Des épisodes infectieux sont survenus chez environ 2% des patients (0,5% des cycles), ont été associés à une neutropénie sévère chez environ 2,1 % des patients (0,5% des cycles), et ont entraîné 1 décès.

Une anémie a été rapportée chez environ 97,2% des patients (2,1 % avec une hémoglobine < 8 g/dl).

Une thrombocytopénie (< 100000/mm^3^) a été observée chez 32,6% des patients et 21,8% des cycles. Aucune thrombocytopénie sévère n’a été observée (<50000 mm^3^).

Un cas de thrombocytopénie périphérique avec anticorps antiplaquettes est rapporté dans l'expérience clinique obtenue après la mise sur le marché.

INFECTION

De rares cas d’insuffisance rénale, d’hypotension ou de collapsus cardio-vasculaire ont été observés chez des patients atteints d’infection systémique grave.

EFFETS INDESIRABLES GENERAUX ET REACTIONS AU SITE D’INJECTION

*Syndrome cholinergique aigu:*

Un syndrome de type cholinergique aigu, transitoire et sévère est observé chez 9% des patients traités par monothérapie et chez 1,4 % des patients traités par association. Les principaux symptômes sont définis par une diarrhée précoce et un ensemble de symptômes tels que crampe abdominale, conjonctivite, rhinite, hypotension, vasodilatation, hypersudation, refroidissement, malaise, vertige, troubles visuels, myosis, larmoiement et hypersalivation survenant pendant ou dans les 24 heures suivant l'administration de CAMPTO. Les symptômes cèdent à l’administration d’atropine (voir «Mises en garde spéciales et précautions particulières d’emploi»).

L'asthénie sévère est observée chez moins de 10% des patients traités par monothérapie et chez 6,2% des patients traités par association. L'imputabilité à CAMPTO n'est pas clairement établie. Une fièvre isolée non accompagnée d'infection ou de neutropénie sévère est survenue chez 12 % des patients traités par monothérapie et chez 6,2 % des patients traités par association.

Des réactions modérées au site d’injection ont été rapportées peu fréquemment.

*Troubles cardiovasculaires:*

De rares cas d’hypertension pendant ou suivant la perfusion ont été rapportés.

*Troubles respiratoires:*

Les cas de pathologie pulmonaire interstitielle avec infiltrats pulmonaires sont peu fréquents sous CAMPTO.

Des effets précoces tels que dyspnée ont été rapportés (voir rubrique 4.4).

*Atteintes cutanées et sous-cutanées:*

L'alopécie est très fréquente et réversible.

Des réactions cutanées d’intensité modérée et peu fréquentes ont été rapportées.

*Troubles du système immunitaire:*

Des réactions allergiques d’intensité modérée et peu fréquentes et de rares cas de réactions de type anaphylactique/anaphylactoïde ont été rapportés**.**

*Troubles musculo-squelettiques:*

Des effets précoces tels que contraction musculaire ou crampes et paresthésies ont été rapportés.

**Examens biologiques**

En monothérapie, une augmentation transitoire mineure à modérée des taux sériques soit des transaminases, soit des phosphatases alcalines, soit de la bilirubine est observée respectivement chez 9,2%, 8,1% et 1,8% des patients, en l'absence de progression des métastases hépatiques. Une augmentation transitoire mineure à modérée des taux sériques de créatinine est observée chez 7,3 % des patients.

En association, une élévation sérique transitoire (grade 1 et 2) des ALAT, ASAT, phosphatases alcalines ou bilirubine a été observée chez 15%, 11%, 11% et 10% des patients respectivement, en l'absence de progression des métastases hépatiques. Des grades 3 transitoires ont été observés chez 0%, 0%, 0% et 1% des patients respectivement. Aucun grade 4 n'a été observé.

Dans de très rares cas, une élévation de l’amylase sérique et/ou de la lipase sérique a été rapportée.

De rares cas d’hypokaliémie et d’hyponatrémie principalement liées à une diarrhée et des vomissements ont été rapportés.

**TROUBLES DU SYSTEME NERVEUX**

De très rares cas de troubles du langage transitoires associés aux perfusions de CAMPTO ont été rapportés.

4.9       Surdosage

Des cas de surdosages pouvant être fatals à des doses supérieures approximativement à deux fois la dose thérapeutique recommandée, ont été rapportés.

Les effets indésirables les plus fréquents ont été les neutropénies sévères et les diarrhées sévères. Il n'existe aucun antidote connu en cas de surdosage par CAMPTO.

Par conséquent, en cas de surdosage, il est recommandé de prévenir la déshydratation causée par la diarrhée et de traiter les complications infectieuses.

5. PROPRIÉTÉS PHARMACOLOGIQUES

5.1       Propriétés pharmacodynamiques

Cytotoxique inhibiteur de la topoisomérase I. Code ATC: L01XX19

(L : anticancéreux et immunosuppresseur)

**Données précliniques**

L'irinotecan est un dérivé hemi-synthétique de la camptothécine. Il s'agit d'un agent antinéoplasique qui agit comme inhibiteur spécifique de l'ADN topoisomérase I. L'irinotecan est métabolisé par la carboxylestérase dans la plupart des tissus en un métabolite actif, le SN-38 qui s'est révélé plus actif que l'irinotecan sur la topoisomérase I purifiée et plus cytotoxique sur plusieurs lignées de cellules tumorales murines ou humaines. L'inhibition de l'ADN topoisomérase I par l'irinotecan ou le SN-38 induit des lésions simple-brin de l'ADN qui bloquent la fourche de réplication de l'ADN et sont responsables de l'activité cytotoxique. Celle-ci est fonction du temps du contact avec les cellules et est spécifique de la phase S.

*In vitro*, l'irinotecan et le SN-38 ne sont pas significativement reconnus par la P-glycoprotéine MDR et exercent des effets cytotoxiques sur des lignées cellulaires résistantes à la doxorubicine et à la vinblastine.

Par ailleurs, l'irinotecan possède un large spectre d'activité antitumorale expérimentale *in vivo* sur les tumeurs murines (adénocarcinome du canal pancréatique PO3, adénocarcinome mammaire MA 16/C, adénocarcinomes coliques C38 et C51) et sur des xénogreffes humaines (adénocarcinome colique Co-4, adénocarcinome mammaire Mx-1, adénocarcinomes gastriques ST-15 et SC-16). L'irinotecan est également actif sur des tumeurs murines exprimant la P-glycoprotéine MDR (leucémies P388 résistants à la vincristine ou à la doxorubicine).

Le principal effet pharmacologique de l'irinotecan non lié à son activité antitumorale est l'inhibition de l'acétylcholinestérase.

**Données cliniques**

En monothérapie:

• Des essais cliniques de phase II/III ont été conduites chez plus de 980 patients atteints d'un cancer colorectal métastatique en échec d'un traitement antérieur par 5FU et traités toutes les 3 semaines. L'efficacité antitumorale de CAMPTO a été évaluée chez 765 patients en progression documentée sous 5FU à l'entrée dans l'essai.

|  | Phases III | | | | | |
| --- | --- | --- | --- | --- | --- | --- |
|  | CAMPTO versus soins palliatifs | | | CAMPTO versus 5FU | | |
|  | CAMPTO | Soins palliatifs | p | CAMPTO | 5FU | p |
|  | n=183 | n= 90 |  | n=127 | n=129 |  |
| Survie sans progression à 6 mois (%) | NA | NA | -- | 33,5 * | 26,7 | p=0,03 |
| Survie à 1 an (%) | 36,2* | 13,8 | p=0,0001 | 44,8* | 32,4 | p=0.0351 |
| Survie médiane (mois) | 9,2* | 6,5 | p= 0,0001 | 10,8* | 8,5 | p=0,0351 |

NA : Non Applicable

* : différence statistiquement significative

Dans les essais de phase II, réalisés chez 455 patients traités par le schéma d'administration toutes les 3 semaines, la survie sans progression à 6 mois a été de 30% et la survie médiane de 9 mois. Le temps médian jusqu’à progression était de 18 semaines.

De plus, des essais de phase II, non comparatifs, ont été réalisés chez 304 patients traités avec un schéma d'administration hebdomadaire, dont 193 patients à la dose de 125 mg/m², en perfusion intraveineuse de 90 minutes, 4 semaines consécutives suivies de 2 semaines de repos. Dans ces essais, le temps médian jusqu'à progression était de 17 semaines, et la survie médiane était de 10 mois. Un profil de tolérance similaire à celui du schéma toutes les 3 semaines a été observé avec le schéma hebdomadaire, chez les 193 patients ayant débuté le traitement à la dose de 125 mg/m². Le délai médian d'apparition de la première selle liquide était de 11 jours.

En association :

• Un essai de phase III a été réalisé chez 385 patients non prétraités pour un cancer colorectal métastatique recevant soit un schéma d’administration toutes les 2 semaines, (cf. "Posologie et modes d'administration"), soit un schéma d’administration hebdomadaire.

Dans le schéma toutes les 2 semaines, au jour 1, l'administration de CAMPTO à la dose de 180 mg/m² une fois toutes les 2 semaines est suivie d'une perfusion d'acide folinique (200 mg/m² en perfusion de 2 heures) et de 5-fluorouracile (400 mg/m² en bolus suivi par 600 mg/m² en perfusion de 22 heures).

Au jour 2, l'acide folinique et le 5-fluorouracile sont administrés aux mêmes doses et selon le même schéma. Dans le schéma hebdomadaire, l'administration de CAMPTO à la dose de 80 mg/m² est suivie d'une perfusion d'acide folinique (500 mg/m² en perfusion de 2 heures), puis de 5-fluorouracile (2300 mg/m² en perfusion de 24 heures) pendant 6 semaines.

Dans l'essai d'association selon les 2 schémas décrits ci-dessus, l'efficacité de CAMPTO a été évaluée chez 198 patients

|  | Résultats globaux (n=198) | | Schéma hebdomadaire (n=50) | | Schéma toutes les 2 semaines (n=148) | |
| --- | --- | --- | --- | --- | --- | --- |
|  | CAMPTO + 5FU/AF | 5FU/AF | CAMPTO + 5FU/AF | 5FU/AF | CAMPTO ^+^  5FU/AF | 5FU/AF |
| Taux de réponse (%) | 40,8* | 23,1* | 51,2* | 28,6* | 37,5* | 21,6* |
| p | p<0,001 | | p=0,045 | | p=0,005 | |
| Temps médian jusqu'à | 6,7 | 4,4 | 7,2 | 6,5 | 6,5 | 3,7 |
| progression (mois) |  |  |  |  |  |  |
| p |  |  |  |  |  |  |
|  | p<0,001 | | NS | | p=0,001 | |
| Durée médiane des | 9,3 | 8,8 | 8,9 | 6,7 | 9,3 | 9,5 |
| réponses (mois) |  | |  | |  | |
| p | NS | | p=0,043 | | NS | |
| Durée médiane des | 8,6 | 6,2 | 8,3 | 6,7 | 8,5 | 5,6 |
| réponses et des |  | |  | |  | |
| stabilisations (mois) |  | |  | |  | |
| p | p<0,001 | | NS | | p=0,003 | |
| Temps médian jusqu'à | 5,3 | 3,8 | 5,4 | 5,0 | 5,1 | 3,0 |
| échec du traitement (mois) |  | |  | |  | |
| p | p=0,0014 | | NS | | p<0,001 | |
| Survie globale médiane | 16,8 | 14,0 | 19,2 | 14,1 | 15,6 | 13,0 |
| (mois) |  | |  | |  | |
| p | p=0,028 | | NS | | p=0,041 | |

5FU: 5-fluorouracile

AF : acide folinique

NS: non significatif

*: sur patients évaluables

Dans le schéma hebdomadaire, l'incidence des diarrhées sévères est de 44,4 % chez les patients traités par CAMPTO en association avec 5FU/AF et de 25,6 % chez les patients traités par 5FU/AF seul.

L'incidence des neutropénies sévères (nombre de neutrophiles<500/mm^3^) est de 5,8% chez les patients traités par CAMPTO en association avec 5FU/AF et 2,4 % chez les patients traités par 5FU/AF seul.

De plus, le temps médian de détérioration définitive du Performance Status est significativement plus long dans le groupe CAMPTO en association que dans le groupe 5FU/AF seul (p=0,046).

La qualité de vie a été évaluée dans cet essai de phase III à l'aide du questionnaire EORTC QLQ-C30. La détérioration définitive survient constamment plus tardivement dans les groupes CAMPTO. L'évolution du score global de Qualité de vie est légèrement meilleure dans le groupe CAMPTO en association bien que non significative, montrant que CAMPTO est efficace en association tout en conservant la qualité de vie.

En association avec le cetuximab:

Le cetuximab, en association avec l’irinotecan, a été étudié dans 2études cliniques. Au total, 356patients atteints d’un cancer colorectal métastatique exprimant l'EGFR, après échec récent d’un traitement cytotoxique à base d’irinotecan et ayant un indice de performance de Karnofsky d’au moins 60, mais la majorité des patients ayant reçu l’association avaient un indice de performance de Karnofsky supérieur ou égal à 80.

EMR62202‑007: cette étude randomisée a comparé l’association du cetuximab et de l’irinotecan (218patients) avec le cetuximab en monothérapie (111patients).

IMCLCP02‑9923: cette étude à un bras, ouverte, a étudié l’association chez 138patients.

Les données d’efficacité générées par ces études sont résumées dans le tableau suivant:

| **Etude** | **N** | **ORR** | | **DCR** | | **PFS (mois)** | | **OS (mois)** | |
| --- | --- | --- | --- | --- | --- | --- | --- | --- | --- |
|  |  | **n (%)** | **IC 95%** | **n (%)** | **IC 95%** | **Médiane** | **IC 95%** | **Médiane** | **IC 95%** |
| **Cetuximab + irinotecan** |  |  |  |  |  |  |  |  |  |
| EMR 62 202-007 | 218 | 50 (22,9) | 17,5; 29,1 | 121 (55,5) | 48,6; 62,2 | 4,1 | 2,8; 4,3 | 8,6 | 7,6;   9,6 |
| IMCL CP02-9923 | 138 | 21 (15,2) | 9,7; 22,3 | 84 (60,9) | 52,2; 69,1 | 2,9 | 2,6; 4,1 | 8,4 | 7, .2; 10,3 |
| **Cetuximab** | | | |  |  |  |  |  |  |
| EMR 62 202-007 | 111 | 12 (10,8) | 5,7; 18,1 | 36 (32,4) | 23,9; 42,0 | 1,5 | 1,4; 2,0 | 6,9 | 5,6;   9,1 |

DCR=*disease control rate*, taux de contrôle de la maladie (patients avec réponse complète, réponse partielle ou stabilisation de la maladie pendant au moins 6 semaines), IC=intervalle de confiance, ORR=*objective response rate*, taux de réponses objectives (patients avec réponse complète ou réponse partielle), OS=*overall survival*, durée de survie globale, PFS=*progression-free survival*, survie sans progression

L'efficacité de l’association du cetuximab et de l’irinotecan était supérieure à celle du cetuximab en monothérapie en termes de taux de réponses objectives (ORR), de taux de contrôle de la maladie (DCR) et de survie sans progression (PFS). Dans l’essai clinique randomisé, aucun effet sur la durée de survie globale n’a été démontrée (*Hazard ratio* 0,91; p=0,48).

**Données pharmacocinétiques / pharmacodynamiques**

L'intensité des toxicités majeures rencontrées avec CAMPTO (par ex. la leuconeutropénie et diarrhée) est en rapport avec l'exposition (AUC) à la molécule-mère et au métabolite SN38. Des corrélations significatives ont été observées entre la toxicité hématologique (diminution du taux des globules blancs et des polynucléaires neutrophiles au nadir) ou l’intensité de la diarrhée et les valeurs des aires sous la courbe (AUC) de l'irinotecan et de son métabolite SN 38 en monothérapie.

5.2       Propriétés pharmacocinétiques

Au cours d’un essai de phase I chez 60patients traités avec le schéma d’administration recommandé (perfusion intraveineuse de 30 minutes à raison de 100 à 750mg/m² toutes les 3 semaines), l’irinotecan a montré un profil d’élimination biphasique ou triphasique.

La clairance plasmatique moyenne est de 15 l/h/m² et le volume de distribution à l'état d'équilibre (Vss) de 157 l/m². La demi-vie plasmatique moyenne de la première phase, du modèle triphasique, est de 12 minutes, celle de la seconde phase de 2,5 heures et la demi-vie terminale de 14,2heures.

Le SN-38 a montré un profil d'élimination biphasique avec une demi-vie d'élimination terminale moyenne de 13,8 heures.

Les moyennes des concentrations des pics plasmatiques de l’irinotecan et du SN-38, obtenus à la fin de la perfusion à la dose recommandée de 350mg/m2 sont respectivement de 7,7μg/ml et 56ng/ml, avec des aires sous la courbe (AUC) correspondantes de 34μg.h/ml et 451ng.h/ml.

Une large variabilité interindividuelle des paramètres pharmacocinétiques est observée principalement pour le métabolite SN-38.

Une étude pharmacocinétique de population a été réalisée, chez 148 patients atteints d’un cancer colorectal métastatique et traités avec des doses et des schémas d’administration différents, au cours d’essais de phase II. Les paramètres pharmacocinétiques estimés avec un modèle à trois compartiments étaient semblables à ceux observés dans l’étude de phase I. Toutes les études ont montré que l’exposition à l'irinotecan (CPT-11) et au SN-38 augmente proportionnellement avec la dose administrée de CPT-11; la pharmacocinétique est indépendante du nombre de cycles administrés et du schéma d'administration.

*In vitro*, la liaison aux protéines plasmatiques est d’environ 65% pour l’irinotecan et de 95% pour le SN‑38.

L'équilibre de masse et les études de métabolisme avec le Carbone 14, ont montré que plus de 50 % de la dose d'irinotecan administrée par voie intraveineuse est excrétée sous forme inchangée, dont 33 % principalement dans les fécès via la bile et 22 % dans les urines.

Deux voies métabolisent chacune, au moins 12 % de la dose :

• l’hydrolyse par carboxylesterase avec formation du métabolite actif, le SN-38, qui est principalement éliminé par glucuronidation, puis par excrétion biliaire et rénale (moins de 0,5 % de la dose d’irinotecan).

     Le SN-38 glucuronidé est par la suite probablement hydrolysé dans l'intestin.

• l’oxydation dépendante du cytochrome P450 3A, aboutissant à l'ouverture extérieure du noyau piperidine avec la formation d'APC (le dérivé de l’acide aminopentanoique) et de NPC (le dérivé de l’amine primaire).

L’irinotecan inchangé est l'entité principale dans le plasma, suivi par l’APC, le SN-38 glycuroconjugué et le SN-38. Seul le SN-38 possède une activité cytotoxique significative.

La clairance d’irinotecan est diminuée d’environ 40 % chez les patients ayant une bilirubinémie comprise entre 1,5 et 3 fois la LSN. Chez ces patients, la posologie de 200 mg/m² d’irinotecan entraîne une exposition plasmatique comparable à celle observée chez les patients ayant des paramètres hépatiques normaux et recevant la posologie de 350 mg/m².

5.3       Données de sécurité précliniquess

L'irinotecan et le SN-38 se sont révélés mutagènes dans le test d'aberration chromosomique *in vitro* dans les cellules CHO ainsi que dans le test du micronoyau *in vivo* chez la souris.

Toutefois, aucun effet mutagène de ces substances n'a été mis en évidence dans le test d'Ames.

Chez les rats traités une fois par semaine pendant 13 semaines à la dose maximale de 150 mg/m² (dose qui est moins de la moitié de la dose recommandée chez l'Homme), aucune tumeur imputable au traitement n'a été rapportée 91 semaines après la fin du traitement. Des études de toxicité à dose unique ou répétée avec CAMPTO ont été réalisées chez la souris, le rat et le chien. Les principaux effets toxiques ont été relevés dans les systèmes hématopoïétiques et lymphatiques.

Chez le chien, une diarrhée tardive associée à une atrophie et une nécrose localisée de la muqueuse intestinale a été rapportée.

Une alopécie a également été observée chez le chien.

La sévérité de ces effets était dose-dépendante et réversible.

6. DONNÉES PHARMACEUTIQUES

6.1       Liste des excipients

Sorbitol

Acide lactique

Hydroxyde de sodium (qs pH 3,5)

Eau pour préparations injectables

6.2       Incompatibilités

Aucune incompatibilité n'est connue à ce jour.

Ne pas mélanger avec d'autres médicaments.

6.3       Durée de conservation

Avant dilution : 3 ans.

Après reconstitution : voir rubrique 6.4 : "Précautions particulières de conservation".

6.4       Précautions particulières de conservation

A conserver à l'abri de la lumière.

La solution de CAMPTO ne contenant pas de conservateur doit être utilisée immédiatement après dilution. Cependant, si la dilution est réalisée sous stricte condition aseptique, la solution de CAMPTO pour perfusion conservée à température ambiante peut être utilisée (temps de perfusion compris) dans les 12 heures après la préparation ou dans les 24 heures après la préparation si elle est conservée au réfrigérateur (+ 2° à + 8° C).

6.5       Nature et contenu de l'emballage extérieur

Flacon en verre brun de 5 ml, avec un bouchon en élastomère butylhalogéné avec surface interne revêtue de téflon.

6.6       Instructions pour l'utilisation, la manipulation et l'élimination

Comme tous les agents antinéoplasiques, CAMPTO doit être préparé et manipulé avec précaution. Il est indispensable d'utiliser des lunettes de protection, un masque et des gants.

En cas de contact cutané avec la solution à diluer ou la solution à perfuser, il convient d'éliminer immédiatement et soigneusement le produit à l'eau et au savon. En cas de contact d'une muqueuse avec la solution à diluer ou la solution à perfuser, celle-ci doit être lavée immédiatement à grande eau.

**Préparation de la solution à perfuser**

Comme tous les autres médicaments utilisés par voie injectable, la solution de CAMPTO POUR PERFUSION DOIT ÊTRE PRÉPARÉE DE FAÇON ASEPTIQUE (voir "Précautions particulières de conservation").

En cas de présence de précipité dans les flacons ou dans la solution diluée, le produit doit être jeté selon les procédures classiques de traitement des déchets cytotoxiques.

A l'aide d'une seringue graduée, prélever dans le flacon la quantité voulue de la solution de CAMPTO en veillant à respecter les conditions d'asepsie et l'injecter dans une poche ou un flacon de perfusion de 250 ml contenant soit une solution de chlorure de sodium à 0,9 %, soit une solution glucosée à 5 %. Mélanger soigneusement la solution à perfuser par rotation manuelle.

**Elimination des déchets**

Tout le matériel utilisé pour la dilution et l'administration doit être détruit conformément aux procédures classiques hospitalières de traitement des déchets cytotoxiques.

7. TITULAIRE DE L'AUTORISATION DE MISE SUR LE MARCHÉ

PFIZER HOLDING FRANCE

23-25, avenue du Docteur Lannelongue

75014 PARIS

8. PRÉSENTATIONS ET NUMÉROS D'IDENTIFICATION ADMINISTRATIVE

• 558 824-5 : 5 ml en flacon (verre brun) ; boîte de 1

9. DATE DE PREMIÈRE AUTORISATION/DE RENOUVELLEMENT DE L'AUTORISATION

Sans objet.

10. DATE DE MISE À JOUR DU TEXTE

Sans objet.

CONDITIONS DE PRESCRIPTION ET DE DÉLIVRANCE

Liste I.

Médicament soumis à prescription hospitalière. Prescription réservée aux spécialistes en oncologie ou en hématologie ou aux médecins compétents en cancérologie. Médicament nécessitant une surveillance particulière pendant le traitement

### IV-2 Gemcitabine

RÉSUMÉ DES CARACTÉRISTIQUES DU PRODUIT

Mis à jour : 06/07/2006

1. DÉNOMINATION DU MÉDICAMENT

GEMZAR 1000 mg, poudre pour solution pour perfusion

2. COMPOSITION QUALITATIVE ET QUANTITATIVE

Gemcitabine............................................................................................................... 1000 mg

Sous forme de chlorhydrate de gemcitabine............................................................. 1140 mg

Pour un flacon

Pour les excipients, voir rubrique 6.1.

3. FORME PHARMACEUTIQUE

Poudre pour solution pour perfusion.

4. DONNÉES CLINIQUES

4.1. Indications thérapeutiques

La gemcitabine est indiquée dans le traitement des patients atteints :

• de cancer bronchique non à petites cellules, localement avancé ou métastatique,

• d’adénocarcinome du pancréas localement avancé ou métastatique,

• de cancer de la vessie, au stade invasif,

• de cancer du sein métastatique, en rechute après une chimiothérapie adjuvante/néoadjuvante, en association au paclitaxel. La chimiothérapie antérieure doit avoir comporté une anthracycline sauf si celle-ci est cliniquement contre-indiquée.

4.2. Posologie et mode d'administration

Posologie:

* Adultes :

• Cancer bronchique non à petites cellules

*En monochimiothérapie:*

La dose recommandée est de 1000mg/m², administrée en perfusion intraveineuse de 30 minutes. L'administration doit être répétée une fois par semaine pendant trois semaines, suivie d'une semaine de repos. Ce cycle de quatre semaines sera alors renouvelé. Une réduction ou un report de la dose avant chaque administration de la chimiothérapie, pourra être envisagé en fonction de la tolérance individuelle des patients.

*En association:*

La gemcitabine en association avec le cisplatine peut être administrée selon deux schémas posologiques; l’un est basé sur des cycles de trois semaines, l’autre sur des cycles de quatre semaines.

L’administration par cycles de trois semaines est le schéma  usuel; le cycle de trois semaines comprend une administration de 1250mg/ m² de gemcitabine en perfusion intraveineuse de 30 minutes, les Jours 1 et 8 suivie d’une semaine de repos pour un cycle de 21 jours. Ce cycle de trois semaines sera alors renouvelé. Une réduction ou un report de la dose avant chaque administration de la chimiothérapie pourra être envisagé en fonction de la tolérance individuelle des patients.

Le cycle de quatre semaines comprend une administration de 1000mg/m² de gemcitabine en perfusion intraveineuse de 30 minutes, les Jours 1, 8 et 15 suivie d’une semaine de repos pour un cycle de 28 jours. Ce cycle de quatre semaines sera alors renouvelé. Une réduction ou un report de la dose avant chaque administration de la chimiothérapie pourra être envisagé en fonction de la tolérance individuelle des patients.

**Adénocarcinome du pancréas**

La dose recommandée est de 1000mg/m², administrée en perfusion I.V. de 30 minutes. L’administration doit être répétée une fois par semaine pendant 7 semaines consécutives suivies d’une semaine de repos. Puis, à partir du cycle suivant, l’administration doit être répétée une fois par semaine pendant 3 semaines consécutives suivies d’une semaine de repos. Les doses pourront être réduites avant chaque administration de la chimiothérapie, en fonction de la tolérance individuelle des patients à la gemcitabine.

**Cancer de la vessie, au stade invasif**

*En association:*

La dose recommandée de gemcitabine, en association avec le cisplatine, est de 1000mg/m² en perfusion intraveineuse de 30 minutes, les Jours 1, 8 et 15 suivie d’une semaine de repos pour un cycle de 28 jours. Le cisplatine est donné à la dose recommandée de 70mg/m² à J2. Ce cycle de quatre semaines sera alors renouvelé. Une réduction ou un report de la dose avant chaque administration de la chimiothérapie pourra être envisagé en fonction de la tolérance individuelle des patients.

Dans une étude clinique, en association au cisplatine à la dose de 100mg/m², la myélosuppression a été plus importante.

**Cancer du sein**

*Utilisation en association avec le paclitaxel:*

Administration au jour 1 du paclitaxel (175mg/m²) en perfusion intraveineuse d'environ 3 heures suivie de l'administration de gemcitabine (1250mg/m²) en perfusion intraveineuse de 30 minutes aux jours 1 et 8 de chaque cycle de 21 jours. Une réduction de dose pendant ou avant chaque cycle pourra être envisagée en fonction de la tolérance individuelle des patients.

**Quelle que soit l’indication**

Les patients recevant de la gemcitabine doivent être soumis, avant chaque administration, à une surveillance hématologique : numération formule sanguine et plaquettes. Si nécessaire, la dose de gemcitabine sera adaptée comme indiqué ci-dessous.

| **Nombre absolu** |  | **Nombre de plaquettes** | **% de la dose totale** |
| --- | --- | --- | --- |
| **De granulocytes (x 106/l)** |  | **(x 106/l)** |  |
| > 1.000 | et | > 100.000 | 100 |
| 500 – 1.000 | ou | 50.000 – 100.000 | 75 |
| <500 | ou | <50.000 | 0 |

Un examen clinique et des contrôles périodiques des fonctions hépatique et rénale devront être faits afin de détecter une toxicité non-hématologique. Les doses pourront être réduites avant chaque administration de la chimiothérapie, en fonction de la tolérance individuelle des patients.

***Le traitement pourra être suspendu, suivant l’avis du médecin, jusqu’à résolution de la toxicité.***

* Patients âgés :

La gemcitabine a été bien tolérée par les patients de plus de 65 ans. Les données pharmacocinétiques suggèrent que l'âge n'a pas d'effet sur le métabolisme du médicament.

* Enfants:

La gemcitabine a été étudiée chez les enfants dans des études limitées de phase I et de phase II dans différents types de tumeur. Les données de ces études ne permettent pas de définir le rapport bénéfice / risque et la dose de la gemcitabine chez les enfants.

Mode d’administration

Voie intraveineuse stricte.

La gemcitabine est bien tolérée au cours de la perfusion et est généralement facile à administrer. Les cas de réaction au site de l'injection sont rares; aucun cas de nécrose cutanée n’a été rapporté.

En cas d'extravasation, l'administration sera interrompue immédiatement.

**Modalités de manipulation**

| La préparation des solutions injectables de cytotoxiques doit être obligatoirement réalisée par un personnel spécialisé et entraîné ayant une connaissance des médicaments utilisés, dans des conditions assurant la protection de l'environnement et surtout la protection du personnel qui manipule. Elle nécessite un local de préparation réservé à cet usage. Il est interdit de fumer, de manger, de boire dans ce local. Les manipulateurs doivent disposer d'un ensemble de matériel approprié à la manipulation notamment blouses à manches longues, masques de protection, calot, lunettes de protection, gants à usage unique stériles, champs de protection du plan de travail, conteneurs et sacs de collecte des déchets. Les excréta et les vomissures doivent être manipulés avec précaution. Les femmes enceintes doivent être averties et éviter la manipulation des cytotoxiques. Tout contenant cassé doit être traité avec les mêmes précautions et considéré comme un déchet contaminé. L'élimination des déchets contaminés se fait par incinération dans des conteneurs rigides étiquetés à cet effet. |
| --- |

Ces dispositions peuvent être envisagées dans le cadre du réseau de cancérologie (circulaire DGS/DH/98 N° 98/188 du 24 mars 1998) en collaboration avec toute structure adaptée et remplissant les conditions requises.

4.3.      Contre-indication

• Hypersensibilité connue à la gemcitabine et aux excipients

• Association concomitante de la gemcitabine et de la radiothérapie, en raison du risque de radiosensibilisation, d'apparition de fibroses pulmonaires et oesophagiennes sévères.

• En association avec le vaccin antiamarile (fièvre jaune) (cf. rubrique 4.5 Interactions avec d’autres médicaments ou autres formes d’interactions).

• Association gemcitabine/cisplatine chez l'insuffisant rénal sévère.

4.4. Mises en garde spéciales et précautions particulières d'emploi

Mises en gardes

En cas d'utilisation successive, la possibilité de radiosensibilisation grave justifie qu'un intervalle d'au moins 4 semaines sépare la chimiothérapie par la gemcitabine de la radiothérapie. Ce délai peut être raccourci si l'état clinique du patient l'exige.

Une toxicité accrue a été démontrée en cas d'allongement du temps de perfusion et d'augmentation de la fréquence d'administration.

Comme d'autres agents cytotoxiques, la gemcitabine peut induire une aplasie médullaire qui se traduit par une anémie, une leucopénie et une thrombocytopénie. Cette thrombocytopénie est souvent sévère et nécessite parfois le recours aux transfusions plaquettaires. Toutefois, la myélosuppression est de courte durée et ne nécessite généralement pas de réduction de la dose et rarement l'arrêt du traitement.

Hypersensibilité : de rares cas de réaction anaphylactique ont été rapportés.

Ce médicament est déconseillé avec les vaccins vivants atténués, la phénytoïne ou la fosphénytoïne (cf. rubrique 4.5 Interactions avec d’autres médicaments ou autres formes d’interactions).

Précautions d’emploi

Les patients recevant de la gemcitabine doivent faire l'objet d'un suivi attentif. Les paramètres biologiques doivent être contrôlés par un laboratoire d'analyses médicales. Le traitement de l'éventuelle toxicité du médicament peut être requis.

Le traitement doit être instauré avec prudence chez les patients dont la fonction médullaire est déficiente. Comme pour d'autres cytotoxiques, il convient d'envisager la possibilité d'aplasie médullaire cumulée en cas de chimiothérapie combinée ou séquentielle.

Les patients recevant de la gemcitabine doivent faire l'objet, avant chaque administration, d'un suivi hématologique avec numération formule sanguine et plaquettes. Une suspension ou une modification du traitement doit être envisagée chaque fois qu'une toxicité médullaire induite par le médicament est décelée (voir rubrique 4.2). Les numérations globulaires peuvent continuer à diminuer après l'arrêt du traitement.

La gemcitabine sera utilisée avec prudence chez les insuffisants hépatiques en l’absence d’étude. Une insuffisance rénale avec une clairance de la créatinine comprise entre 30ml/mn et 80ml/mn n’a pas d’effet significatif sur la pharmacocinétique de la gemcitabine.

L'utilisation de la gemcitabine devra être évitée chez la femme enceinte ou allaitante (voir rubrique 4.6).

Ce médicament contient 17,5 mg de sodium par flacon : en tenir compte chez les personnes suivant un régime hyposodé strict.

4.5. Interactions avec d'autres médicaments et autres formes d'interactions

Interactions communes à tous les cytotoxiques

En raison de l’augmentation du risque thrombotique lors des affections tumorales, le recours à un traitement anticoagulant est fréquent. La grande variabilité de la coagulabilité au cours de ces affections, à laquelle s’ajoute l’éventualité d’une interaction entre les anticoagulants oraux et la chimiothérapie anticancéreuse, imposent, s’il est décidé de traiter le patient par anticoagulant oraux, d’augmenter la fréquence des contrôles de l’INR.

Association contre-indiquée (cf. rubrique 4.3 Contre-indications)

**+ Vaccin antiamarile (fièvre jaune)**

Risque de maladie vaccinale généralisée mortelle.

Associations déconseillées (cf. rubrique 4.4. Mises en garde spéciales)

**+ Phénytoïne (et, par extrapolation, fosphénytoïne)**

Risque de survenue de convulsions par diminution de l'absorption digestive de la seule phénytoïne par le cytotoxique, ou bien risque de majoration de la toxicité ou de perte d'efficacité du cytotoxique par augmentation de son métabolisme hépatique par la phénytoïne ou la fosphénytoïne.

**+ Vaccins vivants atténués sauf antiamarile**

Risque de maladie vaccinale généralisée éventuellement mortelle. Ce risque est majoré chez les sujets déjà immunodéprimés par la maladie sous-jacente

Utiliser un vaccin inactivé lorsqu'il existe (poliomyélite).

Association à prendre en compte

**+ Immunosuppresseurs**

Immunodépression excessive avec risque de syndrome lymphoprolifératif.

4.6. Grossesse et allaitement

L’innocuité de la gemcitabine chez la femme enceinte n’a pas été établie. Le médicament s’étant révélé embryotoxique, fœtotoxique et tératogène dans les expérimentations animales, l’utilisation de la gemcitabine doit être évitée au cours de la grossesse et de l’allaitement à cause du risque potentiel pour le fœtus et l’enfant.

4.7. Effets sur l'aptitude à conduire des véhicules et à utiliser des machines

La gemcitabine pouvant induire une somnolence, les patients doivent s’abstenir de conduire un véhicule ou d’utiliser des machines tant que ce type de réaction n’a pas été exclu.

4.8. Effets indésirables

* Hématologiques

La gemcitabine peut induire une aplasie médullaire, entraînant une anémie, une leucopénie et une thrombocytopénie. La myélosuppression est généralement modérée, elle est plus prononcée pour la lignée granulocytaire. La thrombocytémie est un autre effet fréquemment rapporté.

* Hépatiques :

Des augmentations des transaminases hépatiques sont observées. Elles sont habituellement faibles, transitoires et ne nécessitent que rarement l'arrêt du traitement. La prudence s'impose toutefois chez les patients dont la fonction hépatique est altérée.

* Oeso-Gastro-intestinaux :

Nausées, parfois accompagnées de vomissements. Ces effets secondaires justifient des mesures thérapeutiques dans approximativement 20% des cas, mais n'imposent que rarement la diminution de la dose et sont faciles à traiter par les anti-émétiques classiques. Diarrhées, toxicité buccale à type de mucite.

* Pulmonaires :

Dans les heures qui suivent l'injection de gemcitabine, les patients peuvent présenter une dyspnée qui est généralement d'intensité faible et de courte durée. Elle nécessite rarement une réduction de la posologie et disparaît habituellement sans traitement spécifique. Son mécanisme est inconnu et sa relation avec la gemcitabine n'est pas claire.

Des cas d'œdème pulmonaire, de pneumopathies interstitielles et de syndrome de détresse respiratoire de l'adulte (ARDS), d'étiologie inconnue ont été rapportés au cours du traitement par gemcitabine. Dès leur survenue, l'arrêt de la gemcitabine doit être envisagé.

* Rénaux :

Une protéinurie et une hématurie modérées surviennent chez près de la moitié des patients, mais sont rarement significatives sur le plan clinique ; elles ne sont habituellement pas associées à des modifications de la créatinine sérique ou de l'urémie. On a cependant rapporté quelques cas d’insuffisance rénale d'étiologie incertaine.

Aucune toxicité rénale cumulative n'a été observée. (voir rubrique 4.4).

Des manifestations cliniques compatibles avec un syndrome hémolytique et urémique ont été rapportées chez les patients recevant de la gemcitabine. Le traitement par gemcitabine doit être interrompu dès les premiers signes d'anémie hémolytique micro-angiopathique tels qu'une chute brutale de l'hémoglobine avec thrombocytopénie concomitante, élévation de la bilirubine sérique, de la créatinine sérique, de l'urée sanguine ou de la LDH. L'insuffisance rénale peut ne pas être réversible même à l'arrêt du traitement et une dialyse peut être nécessaire.

* Allergiques :

Des éruptions peuvent survenir et s’accompagner de prurit. L'éruption est habituellement faible, ne nécessite pas de réduction posologique et répond à un traitement local. Une desquamation, une vésiculation et une ulcération sont des effets secondaires rapportés occasionnellement.

Un bronchospasme a parfois été rapporté. Ce bronchospasme est habituellement d'intensité modérée et passager, mais il peut requérir un traitement parentéral. La gemcitabine ne doit pas être administrée aux patients ayant une hypersensibilité connue à ce produit. De rares cas de réaction anaphylactique ont été rapportés.

* Cardiaques :

Des cas d'infarctus du myocarde, d'insuffisance cardiaque congestive et d'arythmie ont été observés.

On a rapporté quelques cas d'hypotension.

* Cutanés:

Des manifestations cutanéo-musculaires sévères à type de dermato-polymyosite, au niveau du site antérieurement irradié, ont été rapportées après administration successive de radiothérapie et gemcitabine.

* Autres :

Un syndrome grippal rarement sévère peut survenir. Il est généralement de courte durée et nécessite rarement une diminution de la posologie. Fièvre, céphalées, dorsalgie, frissons, myalgies, asthénie et anorexie sont les symptômes les plus communément rapportés.

De même, une toux, une rhinite, des malaises, des sueurs et une insomnie sont couramment signalés. La fièvre et l'asthénie sont également rapportées comme symptômes isolés. Le mécanisme à la base de cette toxicité est inconnu. Le paracétamol peut en atténuer les symptômes.

Oedème périphérique, très rarement œdème facial. L'œdème périphérique est habituellement modéré et n'impose que rarement une réduction de la posologie, mais peut être douloureux ; il est généralement réversible après l'arrêt de la gemcitabine. Le mécanisme à la base de cette toxicité est inconnu. Il n'y a aucune association avec des signes d'insuffisance cardiaque, hépatique ou rénale.

Les effets secondaires suivants sont aussi couramment rapportés : alopécie (en général minime), somnolence.

4.9. Surdosage

Il n'y a pas d'antidote connu à la gemcitabine. Des doses uniques allant jusqu'à 5,7g/m² ont fait l'objet de perfusion IV en 30 minutes toutes les deux semaines avec une toxicité acceptable sur le plan clinique.

Si on suspecte un surdosage, le patient fera l'objet d'un suivi comprenant les numérations globulaires appropriées et recevra, si nécessaire, un traitement d'appoint.

5. PROPRIÉTÉS PHARMACOLOGIQUES

5.1. Propriétés pharmacodynamiques

Antimétabolite

Code ATC: L01BC05

(L: anticancéreux – immunosuppresseurs)

Activité cytotoxique in vitro :

La gemcitabine possède une activité cytotoxique significative sur diverses cellules murines et cellules tumorales humaines en culture. La gemcitabine est un antimétabolique spécifique de la phase S du cycle cellulaire (phase de synthèse de l'ADN), elle bloque, dans certaines circonstances, la progression cellulaire au-delà de la phase G1/S. In vitro, l'action cytotoxique de la gemcitabine dépend à la fois de sa concentration et du temps.

Activité antitumorale préclinique:

Dans les modèles de tumeurs chez l'animal, l'activité antitumorale de la gemcitabine dépend du schéma d'administration. Administrée quotidiennement, la gemcitabine entraîne la mort des animaux avec une activité antitumorale minimale. Toutefois, lorsque l'on a recours à un schéma thérapeutique avec administration tous les trois ou quatre jours, la gemcitabine peut être administrée à des doses non létales pourvues d'une excellente action antitumorale sur un grand nombre de tumeurs de la souris.

Métabolisme cellulaire et mécanismes d'action :

La gemcitabine (dFdC) est métabolisée dans les cellules par des nucléosides kinases en nucléosides diphosphate (dFdCDP) et triphosphate (dFdCTP) actifs. L'action cytotoxique de la gemcitabine semble due à l'inhibition de la synthèse de l'ADN par la double action du dFdCDP et du dFdCTP. D'abord, le dFdCDP inhibe la ribonucléotide réductase qui agit comme unique catalyseur des réactions qui produisent des désoxynucléosides triphosphates destinés à la synthèse de l'ADN. L'inhibition de cette enzyme par le dFdCDP entraîne une réduction des concentrations de désoxynucléosides en général et du dCTP en particulier. En second lieu, le dFdCTP entre en compétition avec le dCTP pour son incorporation dans l'ADN (auto-potentialisation).

De la même manière, une faible quantité de gemcitabine peut aussi être incorporée dans l'ARN. Ainsi, la réduction de la concentration intracellulaire du dCTP potentialise l'incorporation du dFdCTP dans l'ADN. L'ADN polymérase epsilon est incapable d'écarter la gemcitabine et de réparer les chaînes d'ADN en cours de formation.

Après incorporation de la gemcitabine dans l'ADN, un nucléotide supplémentaire s'ajoute aux chaînes d'ADN en cours d'élongation. A la suite de cette adjonction, on assiste à une inhibition complète de la synthèse de l'ADN (terminaison de chaîne masquée). Après son incorporation dans l'ADN, la gemcitabine induit le processus de lyse cellulaire programmée, connu sous le nom d'apoptose.

5.2. Propriétés pharmacocinétiques

Pharmacocinétique de la gemcitabine :

Les pics plasmatiques mesurés immédiatement après l'administration d'une dose de 1000mg/m² en perfusion de 30 minutes varient de 10 à 40 µg/ml. La demi-vie moyenne terminale est de 17 minutes (extrêmes : 11 et 26 min).

Le volume de distribution moyen du compartiment central est de 11 l/m² (extrêmes : 5 et 21l/m²) et le volume de distribution moyen à l'état d'équilibre (V_ss_) de 17 l/m² (extrêmes : 9et 30 l/m²). La fixation aux protéines plasmatiques est négligeable et la distribution tissulaire non extensive. La clairance systémique moyenne est de 90 l/h/m² (extrêmes : 40et 130l/h/m²). Chez la femme, la clairance est approximativement 30 % plus faible que chez l'homme. Néanmoins, aux doses recommandées, ceci ne requiert pas de diminution de la posologie. Moins de 10 % de la dose administrée sont excrétés sous forme inchangée dans les urines. La valeur de la clairance rénale moyenne est de 2 à 7 l/h/m².

La pharmacocinétique de la gemcitabine chez les patientes atteintes de cancer du sein n’a pas été différente de celle observée chez les patientes atteintes d’un autre type de tumeur solide. Lorsqu’ils sont administrés en association, la pharmacocinétique de la gemcitabine et la pharmacocinétique du paclitaxel ne sont pas modifiées par rapport à leurs pharmacocinétiques en monothérapie. La clairance systémique moyenne du paclitaxel est de 7,89 L/h/m² après administration de paclitaxel en monothérapie et de 7,71 L/h/m² après administration de l’association. La clairance systémique moyenne de la gemcitabine est de 78,7 L/h/m² après administration de gemcitabine en monothérapie et de 76,4 L/h/m² après administration de l’association.

Métabolisme :

La gemcitabine est rapidement métabolisée par la cytidine déaminase dans le foie, les reins, le sang et les autres tissus. Le métabolisme intracellulaire de la gemcitabine produit des mono, di et triphosphates de gemcitabine (dFdCMP, dFdCDP et dFdCTP) parmi lesquels les dFdCDP et dFdCTP sont considérés actifs. Ces métabolites intracellulaires n'ont pas été détectés dans le plasma et l'urine. Le métabolite principal, 2'-déoxy-2', 2'-difluorouridine (dFdU) présent dans le plasma et l'urine est quant à lui inactif.

*Cinétique du dFdCTP* :

Ce métabolite se trouve dans les cellules mononucléaires circulantes. Les informations qui suivent ont trait à ces cellules.

La demi-vie d'élimination terminale varie de 0,7 à 12 heures. Les concentrations intracellulaires augmentent en fonction de la dose de gemcitabine : des doses comprises entre 35 et 350mg/m²/30min donnent des concentrations à l'état d'équilibre de 0,4 à 5µg/ml. Au-delà de concentrations plasmatiques de gemcitabine de 5 µg/ml, les taux de dFdCTP cessent d'augmenter, ce qui suggère que la formation est saturable dans ces cellules. Les concentrations plasmatiques de la gemcitabine consécutives à une dose de 1000 mg/m²/30 min sont supérieures à 5 µg/ml pendant près de 30 minutes après la fin de la perfusion et supérieures à 0,4 µg/ml pendant l'heure qui suit.

*Cinétique du dFdU :*

Le pic plasmatique, 3 à 15 minutes après la fin d'une perfusion de 1000 mg/m² en 30 minutes varie de 28 à 52 µg/ml. Les concentrations les plus basses après une administration hebdomadaire, s'échelonnent de 0,07 à 1,12 µg/ml, sans accumulation apparente. Les concentrations plasmatiques diminuent selon une courbe triphasique. La demi-vie moyenne de la phase terminale est de 65 heures (extrêmes : 33 et 84 heures). Le dFdU formé représente 91 à 98 % de la clairance de la gemcitabine. Le volume de distribution moyen du compartiment central est de 18 l/m² (extrêmes : 11 et 22 l/m²) et le volume de distribution moyen à l'état d'équilibre (V_ss_) de 150 l/m² ( extrêmes : 96 et 228 l/m²). La distribution tissulaire est importante et la clairance moyenne apparente représente 2,5 l/h/m² (extrêmes : 1 et 4 l/h/m²). L'élimination se fait par voie urinaire.

*Elimination globale :*

Pendant la semaine qui suit l'administration, 92 à 98 % de la dose de gemcitabine administrée sont retrouvés, 99 % dans les urines, essentiellement sous forme de dFdU ; 1 % s'élimine par voie fécale.

5.3. Données de sécurité précliniques

Lors d'études à doses répétées, pouvant atteindre 6 mois, réalisées chez la souris et le chien, l'observation principale a été la suppression de l'hématopoïèse. Ces effets, liés aux propriétés cytotoxiques de la substance étaient réversibles après l'arrêt du traitement. La sévérité de l'effet dépendait du schéma thérapeutique et de la dose.

***Oncogénicité, mutagénicité, fertilité* :**

Des altérations cytogénétiques ont été provoquées par la gemcitabine lors d'un test *in vivo*. La gemcitabine a  provoqué des mutations génétiques lors d'un test *in vitro* sur une lignée de cellules de lymphome de souris (L5178Y). La gemcitabine entraîne chez les souris mâles une hypospermatogénèse, réversible, dépendante du schéma thérapeutique et de la dose. Bien que les études animales aient montré un effet de la gemcitabine sur la fertilité masculine, cet effet n'a pas été observé sur la fertilité féminine.

6. DONNÉES PHARMACEUTIQUES

6.1. Liste des excipients

Mannitol, acétate de sodium, acide chlorhydrique, hydroxyde de sodium.

6.2. Incompatibilités

Ce médicament ne doit pas être mélangé avec d’autres médicaments que ceux mentionnés en 6.6.

6.3. Durée de conservation

Avant reconstitution:

3 ans

Après reconstitution:

La stabilité physico-chimique de la solution reconstituée a été démontrée pendant 24 heures à une température ne dépassant pas 30°C. La solution reconstituée ne doit pas être réfrigérée (risque de cristallisation).

Toutefois d’un point de vue microbiologique, la solution reconstituée doit être utilisée immédiatement.

6.4. Précautions particulières de conservation

A conserver à une température ne dépassant pas 30°C.

6.5. Nature et contenu de l'emballage extérieur

Poudre pour solution pour perfusion en flacon (verre) de 50 ml; bouchon (bromobutyle).

6.6. Instructions pour l'utilisation, la manipulation et l'élimination

La manipulation de ce cytotoxique par le personnel infirmier ou médical nécessite un ensemble de précautions permettant d’assurer la protection du manipulateur et de son environnement (voir rubrique 4.2 Posologie et mode d’administration).

Le seul diluant recommandé pour reconstituer la poudre stérile de gemcitabine est le chlorure de sodium à 0,9 % pour préparations injectables sans conservateurs. Bien qu’aucune incompatibilité n’ait été démontrée, il est néanmoins recommandé de ne pas mélanger les solutions de gemcitabine avec d’autres médicaments. Pour des raisons de solubilité, la limite supérieure de concentration de la gemcitabine après reconstitution est de 40 mg/ml. La reconstitution à des concentrations supérieures à 40 mg/ml est à éviter en raison d’une dissolution incomplète.

Reconstitution: ajouter au moins 5 ml de chlorure de sodium pour injection à 0,9 % au flacon à 200 mg ou au moins 25 ml de chlorure de sodium à 0,9 % pour préparations injectables, au flacon à 1000 mg. Agiter jusqu’à dissolution. Les solutions de gemcitabine peuvent être administrées comme préparées ci-dessus ou être diluées avec du chlorure de sodium pour préparations injectables à 0,9 %.

Avant d’être administrées, les substances pour usage parentéral doivent faire l’objet d’une inspection visuelle pour détecter la présence éventuelle de particules ou d’une décoloration.

Comme tout cytostatique, le chlorhydrate de gemcitabine doit être manipulé avec prudence. Les produits non utilisés doivent être détruits conformément aux procédures hospitalières de traitement des déchets cytotoxiques.

7. TITULAIRE DE L’AUTORISATION DE MISE SUR LE MARCHÉ

LILLY France s.a.S.

13, rue Pagès

92158 suresnes cedex

8. PRÉSENTATIONS ET NUMÉROS D’IDENTIFICATION ADMINISTRATIVE

• 559 675-3: poudre pour solution pour perfusion en flacon (verre)

9. DATE DE PREMIÈRE AUTORISATION/DE RENOUVELLEMENT DE L’AUTORISATION

10. DATE DE MISE À JOUR DU TEXTE

CONDITIONS DE PRESCRIPTION ET DE DÉLIVRANCE

Liste I.

Médicament soumis à prescription hospitalière. Prescription réservée aux spécialistes en oncologie ou en hématologie ou aux médecins compétents en cancérologie. Médicament nécessitant une surveillance particulière pendant le traitement.

###

### IV-3 5-Fluorouracile (5-FU)

RÉSUMÉ DES CARACTÉRISTIQUES DU PRODUIT

Mis à jour : 30/05/2005

1. DÉNOMINATION DU MÉDICAMENT

FLUOROURACILE DAKOTA 50 mg/ml, solution à diluer pour perfusion

2. COMPOSITION QUALITATIVE ET QUANTITATIVE

Fluorouracile ................................................................................................................. 50 mg

pour 1 ml de solution à diluer

Un flacon de 5 ml renferme 250 mg de fluorouracile

Un flacon de 10 ml renferme 500 mg de fluorouracile

Un flacon de 20 ml renferme 1 g de fluorouracile

Un flacon de 100 ml renferme 5 g de fluorouracile

Un flacon de 200 ml renferme 10 g de fluorouracile

Pour les excipients, voir rubrique 6.1.

3. FORME PHARMACEUTIQUE

Solution à diluer pour perfusion.

4. DONNÉES CLINIQUES

4.1. Indications thérapeutiques

• Adénocarcinomes digestifs évolués.

• Cancers colorectaux après résection en situation adjuvante.

• Adénocarcinomes mammaires après traitement locorégional ou lors des rechutes.

• Adénocarcinomes ovariens.

• Carcinomes épidermoïdes des voies aérodigestives supérieures et oesophagiennes.

4.2. Posologie et mode d'administration

Posologie

• En monothérapie :

Posologie moyenne de 400 à 600 mg/m²/j, 3 à 6 jours par mois en perfusion IV d’une heure environ.

• En association à d’autres cytotoxiques :

300 à 600 mg/m²/j, 2 à 5 jours par cycles espacés de 3 à 4 semaines.

Plus exceptionnellement :

• FLUOROURACILE DAKOTA peut être administré en perfusion intra-artérielle hépatique lente (4 à 6 heures) à la posologie de 600 mg/m² de façon hebdomadaire.

• Il est parfois utilisé en perfusion veineuse continue à la posologie de 700 mg à

1 g/m² sur 3 à 5 jours consécutifs.

Ces modes d’administration doivent être réservés aux services spécialisés.

La dose de 1 g/m² par injection ne doit pas être dépassée dans la majorité des indications.

Mode d’administration :

Voie intraveineuse.

Exceptionnellement, en perfusion intra-artérielle hépatique lente.

En cas d’extravasation, l’administration sera interrompue immédiatement.

Dilutions :

15 ml de solution injectable peuvent être mélangés à 250 ml de solutions suivantes :

• Chlorure de sodium à 0,9 %,

• Glucose à 5 %,

• Glucose à 10 %,

• Glucose à 2,5 % + chlorure de sodium à 0,45 %,

• Solution de Ringer,

• Solution de Hartmann.

Ne pas administrer par voie intramusculaire.

En cas d’extravasation, l’administration sera interrompue immédiatement.

Modalités de manipulation

| La préparation des solutions injectables de cytotoxiques doit être obligatoirement réalisée par un personnel spécialisé et entraîné ayant une connaissance des médicaments utilisés, dans des conditions assurant la protection de l’environnement et surtout la protection du personnel qui manipule. Elle nécessite un local de préparation réservé à cet usage. Il est interdit de fumer, de manger, de boire dans ce local. Les manipulateurs doivent disposer d’un ensemble de matériel approprié à la manipulation notamment blouses à manches longues, masques de protection, calot, lunettes de protection, gants à usage unique stériles, champs de protection du plan de travail, conteneurs et sacs de collecte des déchets.Les excréta et les vomissures doivent être manipulés avec précaution. Les femmes enceintes doivent être averties et éviter la manipulation des cytotoxiques. Tout contenant cassé doit être traité avec les mêmes précautions et considéré comme un déchet contaminé. L’élimination des déchets contaminés se fait par incinération dans des conteneurs rigides étiquetés à cet effet. |
| --- |

Ces dispositions peuvent être envisagées dans le cadre du réseau de cancérologie (circulaire DGS/DH/98 N° 98/188 du 24 mars 1998) en collaboration avec toute structure adaptée et remplissant les conditions requises.

4.3. Contre-indications

Ce médicament NE DOIT PAS ETRE UTILISE dans les cas suivants :

• hypersensibilité à ce produit,

• femme enceinte ou qui allaite (voir rubrique 4.6),

• malades en mauvais état nutritionnel,

• hypoplasie médullaire,

• infection potentiellement sévère,

• en association avec :

• le vaccin contre la fièvre jaune (voir rubrique 4.5)

Ce médicament est généralement déconseillé en association avec les vaccins vivants atténués, la phénytoïne et la warfarine.

4.4. Mises en garde spéciales et précautions particulières d'emploi

Mises en garde spéciales

La survenue de stomatite et surtout de diarrhée doit imposer un arrêt du traitement jusqu’à disparition des symptômes ; il en est de même si l’on constate la formation d’ulcération gastro-intestinale ou la survenue d’hémorragie quelle qu’en soit la localisation.

Le traitement sera arrêté en cas de granulopénie au-dessous de 2 000 globules blancs par mm^3^ ou de thrombopénie au-dessous de 80 000 plaquettes par mm^3^.

La survenue de manifestations cardiaques doit conduire à l’arrêt immédiat de la perfusion continue de 5-FU. Dans ce cas, sa réintroduction ne doit pas être envisagée.

Les plus grandes précautions doivent être prises lors de l’utilisation du fluorouracile chez le malade débilité, chez ceux ayant subi antérieurement une irradiation pelvienne à hautes doses, chez ceux ayant été traité préalablement par des agents alkylants, en présence d’un envahissement de la moelle osseuse par des tumeurs métastatiques ou encore chez l’insuffisant hépatique ou rénal.

La survenue rare d’accident toxique aigu très sévère (stomatite, diarrhée, neutropénie, encéphalopathie), survenant à la première administration de 5-FU, doit faire évoquer un déficit de l’activité dihydropyrimidine déshydrogénase (DPD), enzyme participant au catabolisme du 5-FU (l’incidence du déficit total en DPD est de 0,01 % dans la population causasienne).

Précautions particulières d’emploi

La formule sanguine sera contrôlée régulièrement pendant la phase initiale, puis toutes les semaines ou tous les quinze jours en période d’entretien. Chez les patients présentant des antécédents cardiaques, alcooliques et/ou tabagiques, il conviendra de pratiquer une surveillance cardiaque intensive et continue au cours des 3 premières cures de 5-FU, lors d’une perfusion IV continue.

La posologie devra être diminuée de moitié ou du tiers dans les cas suivants :

• intervention chirurgicale dans les 30 jours précédant le traitement ;

• troubles graves de la fonction hépatique,

• lorsque les troubles de l’hématopoïèse se manifestent par une granulopénie de 2 000 à 3000 globules blancs par mm^3^ ou de thrombopénie au-dessous de 100 000 plaquettes par mm^3^.

4.5. Interactions avec d'autres médicaments et autres formes d'interactions

En raison de l’augmentation du risque thrombotique lors des affections tumorales, le recours à un traitement anticoagulant est fréquent. La glande variabilité intra-individuelle de la coagulabilité au cours de ces affections, à laquelle s’ajoute l’éventualité d’une interaction entre les anticoagulants oraux et la chimiothérapie anticancéreuse, imposent, s’il est décidé de traiter le patient par anticoagulants oraux, d’augmenter la fréquence des contrôles de l’INR.

Associations contre-indiquées

Vaccins contre la fièvre jaune: risque de maladie vaccinale généralisée mortelle.

Associations déconseillées

Vaccins vivants atténués (sauf fièvre jaune).

Risque de maladie vaccinale généralisée éventuellement mortelle. Ce risque est majoré chez les sujets déjà immunodéprimés par la maladie sous-jacente. Utiliser un vaccin inactivé lorsqu’il existe (poliomy élite).

Phénytoïne (et par extrapolation fosphenytoïne): risque de survenue de convulsions par diminution de l’absorption digestive de la phénytoïne par le cytotoxique ou risque d’augmentation de la toxicité ou de la perte d’efficacité du cytotoxique due à l’augmentation du métabolisme hépatique par la phénytoïne.

Associations à prendre en compte

Immunosuppresseurs (ciclosporine, tacrolimus, sirolimus).

Immunodépression excessive avec risque de lymphoprolifération.

Interactions spécifiques au fluorouracile

Association déconseillée

**+ Warfarine**: augmentation importante de l’effet de l’anticoagulant oral et du risque hémorragique.

Si l’association ne peut pas être évitée, contrôle plus fréquent du taux de prothrombine et surveillance de l’INR.

Associations à prendre en compte

**+ Acide folinique**: potentialisation des effets, à la fois cytotoxiques et indésirables de fluorouracile.

**+ Interferon alpha**: augmentation de la toxicité gastro-intestinale du fluorouracile.

**+ Metronidazole**: augmentation de la toxicité du fluorouracile par diminution de sa clairance.

4.6. Grossesse et allaitement

Le fluorouracile est contre-indiqué pendant la grossesse et allaitement.

4.7. Effets sur l'aptitude à conduire des véhicules et à utiliser des machines

Sans objet.

4.8. Effets indésirables

• Troubles digestifs :

stomatite, mucite, diarrhée (voir rubrique 4.4) ; anorexie, nausées, vomissements ; exceptionnellement : hémorragies digestives.

• Troubles dermatologiques :

coloration brunâtre du trajet veineux, hyperpigmentation, alopécie. Dermatite, éruption de prédominance palmoplantaire (syndrome mains-pieds), rash, urticaire, photosensibilisation.

• Troubles cardiovasculaires :

quelques cas de douleurs précordiales, de modifications transitoires de l’ECG et, exceptionnellement d’infarctus du myocarde ont été rapportés, le plus souvent lors de la première cure et plus précisément vers le 2^ème^ ou 3^ème^ jour.

• Troubles hématologiques :

leucopénie, thrombocytopénie et plus rarement anémie.

• Troubles neurologiques :

ataxie cérébelleuse.

• Troubles oculaires :

hypersécrétion lacrymale.

4.9. Surdosage

Le surdosage se traduit par une majoration des effets secondaires et en particulier des troubles digestifs et hématologiques. Le traitement par Fluorouracile sera impérativement arrêté et un traitement symptomatique sera mis en place.

5. PROPRIÉTÉS PHARMACOLOGIQUES

5.1. Propriétés pharmacodynamiques

Classe pharmacothérapeutique : ANTIMETABOLITES

(L : Antinéoplasiques et immunomodulateurs)

Code ATC: L01BC02

Antinéoplasique cytostatique de la classe des antimétabolites (antipyrimidine). Afin de mieux comprendre l’activité de fluorouracile, il faut rappeler que l’uracile joue un double rôle fondamental dans les tissus à croissance rapide : d’une part, en étant le précurseur (via la thymidilate-synthétase) de la thymine, base nécessaire à la synthèse d’ADN qui préside à la division cellulaire ; d’autre part, en entrant dans la composition des ARNs qui président à la synthèse des protéines et des enzymes cellulaires. Ainsi, le fluorouracile exerce plusieurs effets antimétaboliques :

• tout d’abord, il est métabolisé en 5-fluorodéoxyuridine 5'-monophosphate (FdUMP) qui, en présence de 6-méthylènetétrahydrofolate, se lie à la thymidilate-synthétase, bloquant la méthylation de l’uracile en thymine, provoquant ainsi une inhibition de la synthèse d’ADN qui freine la prolifération cellulaire ;

• d’autre part, il est phosphorylé et triphophaté (FUTP) et incorporé à la place de l’uracile dans les ARNs, entraînant des erreurs de lecture du code génétique lors de la synthèse de protéines et d’enzymes, et de la production de coenzymes inefficaces et de ribosomes immatures ;

• enfin, le fluorouracile inhibe l’uridine-phosphorylase.

5.2. Propriétés pharmacocinétiques

Injecté par voie veineuse, le fluorouracile disparaît rapidement du sang circulant, sa demi-vie étant d’environ six minutes. Cela tient à la fois à :

• sa diffusion tissulaire, très rapide et très sélective dans les tissus tumoraux et à croissance rapide (moelle, muqueuse intestinale) : à la 4^ème^ heure, ces tissus présentent des concentrations six à huit fois supérieures à celles des tissus à croissance normale.

Ceux-ci ne contiennent pratiquement plus de fluorouracile à la 24^ème^ heure, alors que le tissu tumoral contient toujours la même quantité de produit ; à noter que le produit diffuse dans le LCR avec des concentrations plus faibles mais plus durables.

• Son métabolisme très rapide en produits inactifs (CO_2_, urée, %-fluoro-%-alanine...).

Environ 15 % du produit sont éliminés par voie rénale, et 60 à 80 % sont éliminés par voie respiratoire sous forme de CO_2_.

Compte-tenu du faible taux d’excrétion par voie rénale, une réduction de la dose chez les malades rénaux n’est à priori pas nécessaire. En cas d’anurie, procéder à une adaptation de la posologie.

Etant donné que le 5-FU est métabolisé essentiellement dans le foie, une réduction de la dose doit être envisagée chez les patients atteints d’un trouble grave de la fonction hépatique.

Le 5-FU est catabolisé en métabolites inactifs (dihydro-5-fluorouracile, acide 5-fluorouréidopropionique, %-fluoro-$-alanine) par l’intermédiaire de la dihydropyrimidine déshydrogénase (DPD). Un déficit en DPD peut conduire à une augmentation de la toxicité du 5-FU (voir rubrique 4.4).

5.3. Données de sécurité précliniques

Sans objet.

6. DONNÉES PHARMACEUTIQUES

6.1. Liste des excipients

Hydroxyde de sodium, eau pour préparations injectables.

6.2. Incompatibilités

Ce médicament ne doit pas être mélangé avec d’autres médicaments, à l’exception de ceux mentionnés en 6.6.

6.3. Durée de conservation

Avant dilution: 2 ans.

Après dilution: la stabilité physico-chimique a été démontrée pendant 8 heures à une température comprise entre + 15°C et + 25°C.

Toutefois, d’un point de vue microbiologique, une utilisation immédiate est recommandée.

6.4. Précautions particulières de conservation

A conserver à une température comprise entre + 15°C et + 25°C.

Ce médicament est sensible à la lumière, conserver le conditionnement primaire dans l’emballage extérieur.

Une exposition à une température inférieure à +15°C risque de provoquer l’apparition de particules, phénomène réversible par simple réchauffage du flacon.

6.5. Nature et contenu de l'emballage extérieur

5 ml en flacon (verre) avec bouchon (bromobutyle) ; boîte de 6 ou 12.

10 ml en flacon (verre) avec bouchon (bromobutyle) ; boîte de 6.

20 ml en flacon (verre) avec bouchon (bromobutyle) ; boîte de 6.

100 ml en flacon (verre) avec bouchon (bromobutyle) ; boîte de 5.

200 ml en flacon (verre) avec bouchon (bromobutyle ou chlorobutyle): boîte de 2, 4 ou 5.

6.6. Instructions pour l'utilisation, la manipulation et l'élimination

Dilutions

15 ml de solution injectable peuvent être mélangés à 250 ml de solutions suivantes :

• Chlorure de sodium à 0,9 %,

• Glucose à 5 %,

• Glucose à 10 %,

• Glucose à 2,5 % + chlorure de sodium à 0,45 %,

• Solution de Ringer,

• Solution de Hartmann.

La manipulation de ce cytotoxique par le personnel infirmier ou médical nécessite un ensemble de précautions permettant d’assurer la protection du manipulateur et de son environnement (voir rubrique 4.2).

7. TITULAIRE DE L’AUTORISATION DE MISE SUR LE MARCHÉ

DAKOTA PHARM

1-13, boulevard Romain Rolland

75159 PARIS CEDEX 14

8. PRÉSENTATIONS ET NUMÉROS D’IDENTIFICATION ADMINISTRATIVE

• 560 295-6 : 5 ml en flacon (verre) ; boîte de 6

• 560 296-2 : 5 ml en flacon (verre) ; boîte de 12

• 560 297-9 : 10 ml en flacon (verre) ; boîte de 6

• 560 298-5 : 20 ml en flacon (verre) ; boîte de 6

• 562 249-1 : 100 ml en flacon (verre) ; boîte de 5

• 564 743-3 : 200 ml en flacon (verre); boîte de 2

• 564 745-6 : 200 ml en flacon (verre); boîte de 4

• 564 746-2 : 200 ml en flacon (verre); boîte de 5

9. DATE DE PREMIÈRE AUTORISATION/DE RENOUVELLEMENT DE L’AUTORISATION

10. DATE DE MISE À JOUR DU TEXTE

CONDITIONS DE PRESCRIPTION ET DE DÉLIVRANCE

Liste I.

Médicament soumis à prescription hospitalière. Prescription réservée aux spécialistes en oncologie ou en hématologie ou aux médecins compétents en cancérologie. Médicament nécessitant une surveillance particulière pendant le traitement.

### IV-4 Acide folinique

**Nom commerciale: FOLINATE DE CALCIUM   DAKOTA Pharm®   100 mg, 200 mg, 350 mg**

**FORMES et PRÉSENTATIONS** : Lyophilisat pour usage parentéral à 100 mg, 200 mg et 350 mg; *f*lacons, boîtes unitaires.

**COMPOSITION**

Folinate de calcium (DCI), exprimé en acide folinique,  par flacon : 100 mg ou 200 mg ou 350 mg

(sous forme de folinate de calcium : 108,04 mg/fl 100 mg ; 216,08 mg/fl 200 mg ; 378,14 mg/fl 350 mg)

*Excipients :* chlorure de sodium, hydroxyde de sodium.

**INDICATIONS**

Traitement des cancers colorectaux, en association avec le 5-fluorouracile.

**POSOLOGIE ET MODE D'ADMINISTRATION**

**Posologie :**
Schéma thérapeutique le plus utilisé :

- Acide folinique : 200 mg/m^2^/jour en injection intraveineuse lente (10 minutes).
  L'administration d'acide folinique doit être faite avant celle du 5-fluorouracile ;
- 5-fluorouracile : 300 mg/m^2^/jour en perfusion courte ou en bolus.

L'acide folinique et le 5-fluorouracile seront administrés 5 jours de suite toutes les 3 à 4 semaines.

**Mode d'administration :**
Voie intraveineuse.
Diluer le contenu du flacon de 100 mg dans 5 ml d'eau ppi ou du flacon de 200 mg dans 10 ml d'eau ppi ou du flacon de 350 mg dans 17,5 ml d'eau ppi.
La solution obtenue est compatible avec le glucosé isotonique ou le sérum physiologique.

*Recommandations d'utilisation :*

- Utiliser préférentiellement une aiguille de diamètre inférieur à 0,8 mm.
- Maintenir l'aiguille perpendiculaire à la surface et au milieu durant la perforation.
- Tout au long de l'opération, ne pas tourner l'aiguille pour éviter toute détérioration du bouchon.

**CONTRE-INDICATIONS**

Liées à l'administration du 5-fluorouracile :

- Grossesse.
- Allaitement.

**MISES EN GARDE et PRÉCAUTIONS D'EMPLOI**

**Précautions d'emploi :**
L'acide folinique doit être administré avant le 5-fluorouracile et par voie intraveineuse exclusivement.
De plus, ce médicament étant un antagoniste du méthotrexate, il ne doit pas être administré en même temps, sauf dans le cas de protocoles particuliers.
Chez les sujets âgés et chez ceux qui ont eu une radiothérapie au préalable, il est prudent de débuter par des doses faibles de 5-fluorouracile (250 mg en injection bolus).
En cas de diarrhée et de stomatite, il est prudent de réduire les doses de 5-fluorouracile.

**INTERACTIONS**

**Interactions médicamenteuses :**

**Nécessitant des précautions d'emploi :**

- Phénobarbital, primidone, phénytoïne : diminution des taux plasmatiques des anticonvulsivants inducteurs enzymatiques, par augmentation de leur métabolisme hépatique dont les folates représentent un des cofacteurs.

**A prendre en compte :**

- 5-fluorouracile : potentialisation des effets, à la fois cytostatiques et indésirables, du 5-fluorouracile.

**GROSSESSE et ALLAITEMENT**

**Grossesse :**
Les études réalisées chez l'animal ont mis en évidence, à fortes doses, un effet malformatif du folinate de calcium.
Du fait de la co-prescription avec un antimitotique, ce médicament est contre-indiqué en cas de grossesse.

**Allaitement :**
L'utilisation de ce médicament est contre-indiquée en cas d'allaitement du fait de l'administration concomitante de 5-fluorouracile.

**EFFETS INDÉSIRABLES**

Ils sont fonction de la dose et du schéma d'administration du 5-fluorouracile :

- diarrhée : chez le sujet âgé, risque de déshydratation (cf Mises en garde/Précautions d'emploi) ;
- mucite, stomatite (cf Mises en garde/Précautions d'emploi) ;
- réactions cutanées : sécheresse de la peau, érythème ;
- conjonctivite, larmoiement ;
- toxicité hématologique modérée.

**SURDOSAGE**

L'acide folinique est atoxique : aucun surdosage n'a été observé, y compris lors de l'administration de très fortes doses. En effet, la dose nécessaire à l'organisme est seule utilisée, l'excédent est éliminé.

**PHARMACODYNAMIE**

L'acide folinique permet de stabiliser le complexe formé entre le 5-fluorouracile et la thymidylate synthétase, ce qui inhibe l'activité de cette enzyme indispensable à la synthèse de L'ADN. De la sorte, l'acide folinique augmente la cytotoxicité du 5-fluorouracile.

**PHARMACOCINÉTIQUE**

La forme active de l'acide folinique est la forme l. Dans le composé racémique (d,l), 50 % de la dose administrée est active. Par dosage microbiologique, le pic plasmatique de l'acide folinique est obtenu en 30 minutes après administration intramusculaire, avec une biodisponibilité relative de près de 100 %. 35 à 40 % de la dose administrée d'acide folinique est transformée en son métabolite actif, le 5-méthyl-tétrahydrofolate. Le temps de demi-vie terminale de l'acide folinique sous forme lévogyre varie de 30 à 60 minutes, alors que celui de la forme inactive (d) est beaucoup plus long, entre 6 et 8 h. Le temps de demi-vie terminal du 5-méthyltétrahydrofolate est d'environ 3 h. L'excrétion de l'acide folinique est majoritairement rénale, sous forme de 5 et 10-formyl-tétrahydrofolates inactifs.

**SÉCURITE PRÉCLINIQUE**

Les études de toxicité embryofoetale ont été réalisées chez le rat et le lapin aux doses de 500 mg puis 1250 mg/m^2^/j de folinate de calcium. A ces deux doses sont observées des anomalies viscérales dose-dépendantes dans les deux espèces.

**INCOMPATIBILITÉS**

Agents oxydants.

**CONDITIONS DE CONSERVATION**

A conserver à une température inférieure à 25 °C et à l'abri de la lumière.
*Après reconstitution :* la solution (lyophilisat reconstitué dans l'eau ppi) se conserve 4 jours à une température inférieure à 25 °C. La solution reconstituée se conserve 4 jours à une température inférieure à 25 °C dans une solution isotonique de chlorure de sodium à 0,9 % ou dans une solution glucosée à 5 %.

**RENSEIGNEMENTS ADMINISTRATIFS**

AMM 341 193.3 (1996 rév 28. 08. 2003) 100 mg.

341 195.6 (1996 rév 28. 08. 2003) 200 mg.

341 196.2 (1996 rév 28. 08. 2003) 350 mg.

Disponible dans les seules pharmacies hospitalières. Collect.

**Laboratoires DAKOTA Pharm**
1-13, bd Romain-Rolland. 75159 Paris cdx 14
Info médic :
Tél : 08 00 22 25 55

# ANNEXE V : lettre d'information au patient(V3-1)

**essai de phase II randomisé multicentrique d'un traitement par gemcitabine ou d'un TRAITEMENT SEQUENTIEL Par Folfiri.3(CPT-11/Acide folinique/5-FU) / gEMcitabine chez les patients atteints d’un CAncer du pancréas métastatique NON PRE-TRAITE.**

**ETUDE firgem**

*** Il vous est demandé de lire attentivement cette fiche d'information avant de prendre votre décision***

*** Demandez à votre médecin de vous expliquer tout ce que vous n'avez pas pu comprendre ***

**INRODUCTION GENERALE :**

Madame, Monsieur,

L’Association des Gastro-Entérologues Oncologues (AGEO), aussi appelée Promoteur, organise une Recherche Biomédicale dans le traitement de votre maladie (ou étude), à laquelle votre médecin, aussi appelé Investigateur, vous a invité à prendre part. Après avoir été complètement informé par votre médecin et lui avoir posé toutes les questions que vous souhaitiez, et après un délai de réflexion, vous serez libre d’y participer ou non. Avant de prendre votre décision, il est important que vous preniez connaissance des informations relatives à son déroulement qui sont contenues dans ce document. Un délai de réflexion vous sera accordé avant de donner votre réponse. Si vous décidez d’y participer, vous devrez signer un Consentement qui ne déchargera en rien de leur responsabilité les organisateurs de cette étude; vous conserverez tous vos droits garantis par la loi.

Votre participation à cette étude est complètement volontaire et après la signature du Consentement, vous pourrez décider à tout moment de l’interrompre, sans que cela n’affecte la qualité des soins auxquels vous avez droit, ni vos relations avec l’équipe médicale et sans aucun préjudice d’aucune sorte.

*En participant à cette étude, vous contribuez à un effort de recherche qui pourra aider d’autres personnes.*

**INFORMATIONS RELATIVES A L’ETUDE** :

En pratique courante, une molécule anti-cancéreuse, appelée gemcitabine (GEMZAR®), est aujourd’hui indiquée chez les patients ayant une tumeur du pancréas. De plus, deux autres molécules, le 5-fluorouracile associé à l'acide folinique (FU/FOL), et l'irinotecan (CAMPTO®), sont utilisés et sont considérés actuellement comme des traitements standards dans d'autres tumeurs digestive (comme le cancer du colon). Le 5-FU a d'ailleurs été longtemps considéré comme un traitement standard dans votre maladie. Tous ces médicaments sont commercialisés. Plus récemment ils ont été associés (FU/FOL/Irinotecan) et ont montré des résultats prometteurs dans le traitement de tumeurs du pancréas.

Afin de mettre au point un traitement plus performant de votre maladie, l’AGEO propose d'évaluer la tolérance et l'efficacité, d’un traitement par l’assocation FU/FOL/Irinotecan en alternance avec la gemcitabine. Le but de cette étude est d’évaluer l’efficacité et la tolérance de cette séquence thérapeutique en comparaison avec le traitement de référence, la gemcitabine seule.

Si vous acceptez de participer à cette étude, votre médecin pratiquera des examens pour vérifier que vous pouvez participer à l'étude. Ces tests incluent des prélèvements sanguins, un examen médical général (incluant l'histoire de la maladie et la prise de médicaments concomitants), un scanner initial. Pour une plus grande sécurité, la mise en place d’une chambre implantable vous sera proposée, elle permettra de faciliter et de sécuriser les perfusions de chimiothérapie.

**DEROULEMENT DE L’ETUDE** :

**Votre traitement** : le choix du traitement (association FU/FOL/Irinotecan en alternance avec la gemcitabine ou la gemcitabine seule) sera établi par un tirage au sort centralisé dans l’ensemble de la France.

• Si vous devez recevoir le traitement à l’étude (association FU/FOL/Irinotecan en alternance avec la gemcitabine), il sera réalisé selon le schéma qui vous est remis avec cette notice et consistera en :

- une perfusion intraveineuse d’'irinotecan, administré en 60 minutes, le 1er et le 3ème jour de chaque cycle de chimiothérapie. Vous recevrez l'acide folinique et le 5-fluorouracile, en perfusion continue pendant 48 h soit entre le 1er et le 3ème jour.

Pour ces perfusions vous pourrez choisir d’être hospitalisé 48h00 ou de venir en hôpital de jour le premier et le troisième jour de traitement. Ceci sera répété 4 fois, toutes les 2 semaines, pour une durée totale, selon votre tolérance à ce traitement, de 2 mois environ. Des examens sanguins seront effectués avant chaque perfusion afin de contrôler l'effet du traitement sur votre organisme et plus particulièrement sur la moelle osseuse, les reins et le foie. Un scanner sera réalisé 2 mois après votre première perfusion d’'irinotecan pour évaluer l'efficacité de ce traitement.

- 2 semaines après votre dernière perfusion d’'irinotecan, la gemcitabine vous sera administrée par perfusion, pendant 100 minutes. Selon votre tolérance à ce produit, ceci sera répété toutes les semaines, 3 semaines sur 4 (1 semaine de repos après trois perfusions hebdomadaires), pour une durée totale de 2 mois environ. Des examens sanguins seront effectués avant chaque perfusion afin de contrôler l'effet du traitement sur votre organisme et plus particulièrement sur la moelle osseuse, les reins et le foie. Un scanner sera réalisé 2 mois après votre première perfusion de gemcitabine pour évaluer l'efficacité de ce traitement.

L’alternance de ces deux types de chimiothérapie sera réalisée pour une durée totale dépendant de votre tolérance à ces produits et de leur efficacité sur votre maladie.

• Si vous devez recevoir la gemcitabine seule, elle vous sera administrée par perfusion, pendant 100 minutes. Selon votre tolérance à ce produit, ceci sera répété toutes les semaines, 3 semaines sur 4 (1 semaine de repos après trois perfusions hebdomadaires). Des examens sanguins seront effectués avant chaque perfusion afin de contrôler l'effet du traitement sur votre organisme et plus particulièrement sur la moelle osseuse, les reins et le foie. Après votre première perfusion de gemcitabine, un scanner sera réalisé tous les 2 mois pour évaluer l'efficacité de ce traitement.

Par ailleurs, quelque soit votre traitement, et afin d’évaluer votre qualité de vie, il vous sera proposer, tous les deux mois, de remplir un questionnaire.

**EFFETS INDESIRABLES POTENTIELS**

Comme tous les médicaments de ce type, les traitements que vous allez recevoir peuvent être responsables d'effets secondaires indésirables. Pendant ce traitement, vous pourrez présenter :

1. Des nausées et des vomissements qui seront prévenus ou arrêtés par des médicaments correcteurs (antiémétiques) qui vous seront prescrits.
2. Une chute de vos globules blancs circulants qui peut favoriser des infections.
3. Une fatigue anormale.
4. Vous pourrez présenter une diarrhée qui pourra être sévère. Vous devrez prendre du loperamide (Imodium) pour le traitement de cette diarrhée. En cas de diarrhée sévère et persistante, votre médecin décidera d'une éventuelle hospitalisation pendant quelques jours. Une note spécifique de recommandations et une ordonnance vous seront remises à votre sortie de l'hôpital. Il vous est demandé de lire attentivement et de suivre rigoureusement ces recommandations.
5. Une constipation liée à l’irinotecan et/ou au lopéramide. De rares cas d’occlusion, d’hémorragie gastro-intestinale voire de perforation digestive ont été rapportés.
6. Une chute de cheveux réversible peut se produire et vous pourrez avoir une prescription de prothèse capillaire si vous le désirez.
7. Une toxicité cutanée (rougeur, de démangeaison, peau des plantes et des paumes qui pelle).
8. Pendant la perfusion d'irinotecan, vous pouvez ressentir une transpiration abondante, des crampes abdominales, une diarrhée, une augmentation de la salivation, des troubles visuels. Quand ils apparaissent, ces symptômes sont en général modérés et sont bien prévenus ou pris en charge par une injection d'atropine si vous ne présentez pas de contre-indication à ce médicament.
9. Une inflammation de la bouche appelée mucite.
10. De rares cas d’élévation de l’amylase, de la lipase, des enzymes du foie et de la bilirubine.
11. Des effets peu sévères tel qu'une perte d'appétit.

Il est important que vous informiez votre médecin de tout événement secondaire qui pourrait survenir entre deux cures, même si vous pensez qu'il n'est pas relié aux médicaments administrés. En particulier, vous devez informer votre médecin de toute diarrhée ou fièvre qui surviennent entre deux cures de chimiothérapie. De même, toute prescription de médicament doit faire l'objet d'une demande auprès de votre médecin qui vous administre les produits de l'essai.

Tous ces effets secondaires, dont nous tenons à vous donner le descriptif détaillé, ne surviennent pas chez tous les patients et lorsqu’ils surviennent, sont le plus souvent modérés et ne nécessitent pas de modification du traitement. Ils ne justifient pas l’interruption systématique du traitement. Les effets secondaires n’influencent en aucun cas l’efficacité du traitement.

Si, au cours de l'essai, le traitement ne s'avère pas efficace ou source d'effets secondaires indésirables importants, un autre traitement vous sera proposé.

Durant ce traitement, il existe un risque important de malformation de l'enfant à naître en cas de grossesse. Vous ou votre partenaire devez utiliser une contraception efficace pendant cette période. Avant de commencer le traitement, votre médecin s’assurera que vous avez le meilleur moyen adapté à votre cas.

Vous pouvez joindre le Docteur ............................................ et l'équipe soignante au numéro de téléphone suivant. .......................

Votre dossier médical reste naturellement confidentiel. Il pourra être consulté par les autorités de tutelle ou par le personnel mandaté par le promoteur, soumis au secret professionnel. Toutes les données recueillies au cours du protocole seront enregistrées pour un traitement informatisé. Le fichier informatique utilisé pour réaliser la présente recherche a fait l'objet d'une autorisation de la CNIL en application des articles 40-1 et suivant la loi "Informatique et Liberté". Vous pouvez exercer vos droits d'accès et de rectification de ces données auprès du Dr.................................

Si vous acceptez de participer à cet essai, nous vous demandons de bien vouloir signer l'attestation suivante en accord avec la loi du 20 décembre 1988 visant à protéger les personnes se soumettant à une étude biomédicale. Votre consentement écrit ne décharge en aucun cas les médecins qui vous soignent de leur responsabilité médicale, mais vous est demandé afin de confirmer que vous avez été correctement informé de l'étude à laquelle vous allez participer.

Le promoteur de cet essai a souscrit une assurance couvrant la responsabilité de l'investigateur pour la durée de cet essai. Néanmoins, votre affiliation à un régime de sécurité sociale est obligatoire pour pouvoir participer à cette étude.

Cet essai a été soumis au Comité Consultatif de Protection des Personnes dans la Recherche Biomédicale du Groupe Hospitalier Pitié Salpêtrière et a reçu un avis favorable le 02/05/2007.

Conformément à la loi N°2002-303 du 4 mars 2002 vous avez la possibilité d’être tenu informé des résultats globaux de la recherche une fois que celle-ci sera achevée.

**(bras expérimental; bras A)**

# ANNEXE VI : Consentement Eclaire(V3-1)

**essai de phase II randomisé multicentrique d'un traitement par gemcitabine ou d'un TRAITEMENT SEQUENTIEL Par Folfiri.3(CPT-11/Acide folinique/5-FU) / gEMcitabine chez les patients atteints d’un CAncer du pancréas métastatique NON PRE-TRAITE.**

**ETUDE firgem**

Je soussigné(e) NOM : .................................................... Prénom : ................................... certifie avoir été informé(e) par le Dr.................................................. sur le déroulement et l'objet de la recherche intitulée " **Essai de phase II, randomisé, multicentrique d'un traitement par gemcitabine ou d'un traitement séquentiel par FOLFIRI.3(cpt-11/acide folinique/5-fu) / gemcitabine chez les patients atteints d’un cancer du pancréas métastatique non pré-traité. Etude firgem**", évaluant la tolérance et l'efficacité dans ma maladie d’un traitement par l’association **Irinotecan/acide folinique/5-FU** en alternance avec la **gemcitabine**.

Je donne librement mon consentement pour participer à cette étude. Je peux à tout moment décider de me retirer de cette étude, sans avoir de justification à fournir et sans que cet arrêt n'affecte la qualité des soins que je suis en droit d'attendre.

Les objectifs, les bénéfices attendus, les contraintes et les risques m'ont été clairement présentés sur une note écrite qui m'a été remise.

J'ai eu la possibilité de poser les questions qui me paraissaient utiles et de recevoir des réponses claires.

J'accepte que les données me concernant ainsi que les résultats de mon traitement enregistrés à l'occasion de cette étude puissent faire l'objet d'un traitement informatisé, par le promoteur ou pour son compte. J'ai bien noté que le droit d'accès prévu par la loi "Informatiques & Libertés" (article 40) s'exerce à tout moment auprès du Dr ....................................... Je pourrai exercer mon droit de rectification auprès du Dr ...........................................

Je suis affilié à un régime de sécurité sociale.

J'accepte que mon dossier médical soit consulté directement par les Autorités de tutelle ou par le personnel mandaté par le promoteur soumis au secret professionnel. Dans tous les cas, mon nom restera confidentiel.

Si des informations nouvelles concernant l’**Irinotecan, l’acide folinique, le 5-Fu** et la **gemcitabine**, susceptibles de modifier mon consentement, survenaient pendant la durée de l'essai, j'en serais aussitôt averti(e).

Je pourrai demander tout complément d'information, si nécessaire, au Docteur .................................. au numéro de téléphone : ................................

Mon consentement ne décharge en rien l'investigateur et le promoteur de leurs responsabilités morales et légales et je conserve mes droits garantis par la loi.

Un double de ce consentement me sera remis après signature.

| Fait à ………… , le …….    Nom et Signature du Médecin : | Fait à ………… , le …….  Nom et Signature du Patient : |
| --- | --- |

# ANNEXE VIi : DECLARATION D'EVENEMENT INDESIRABLE GRAVE


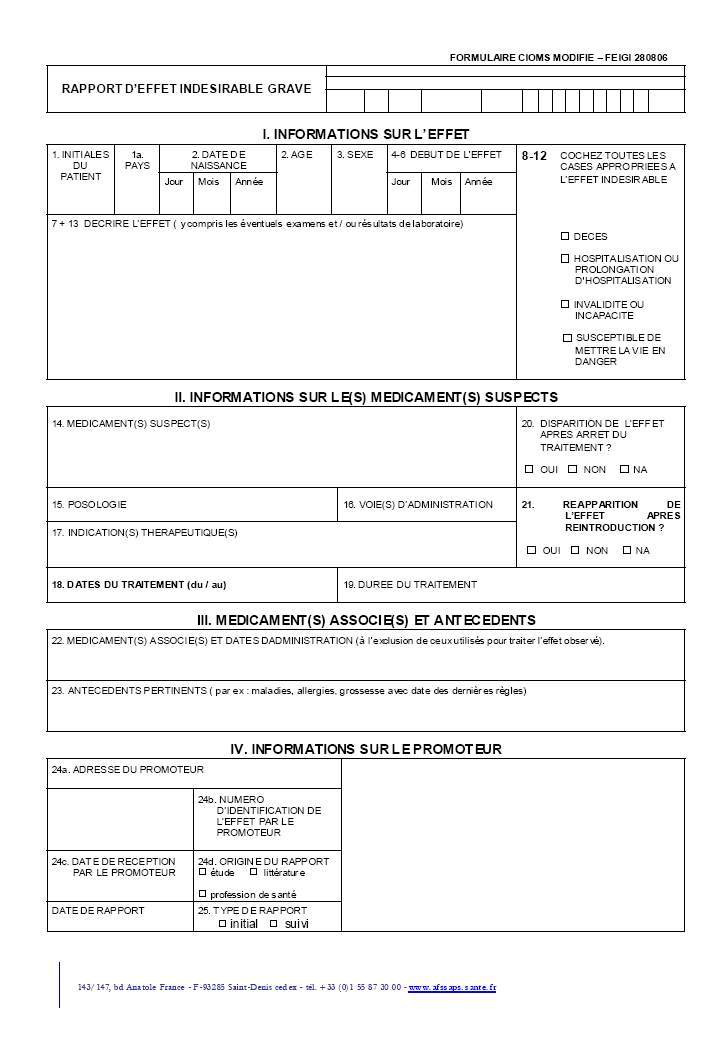


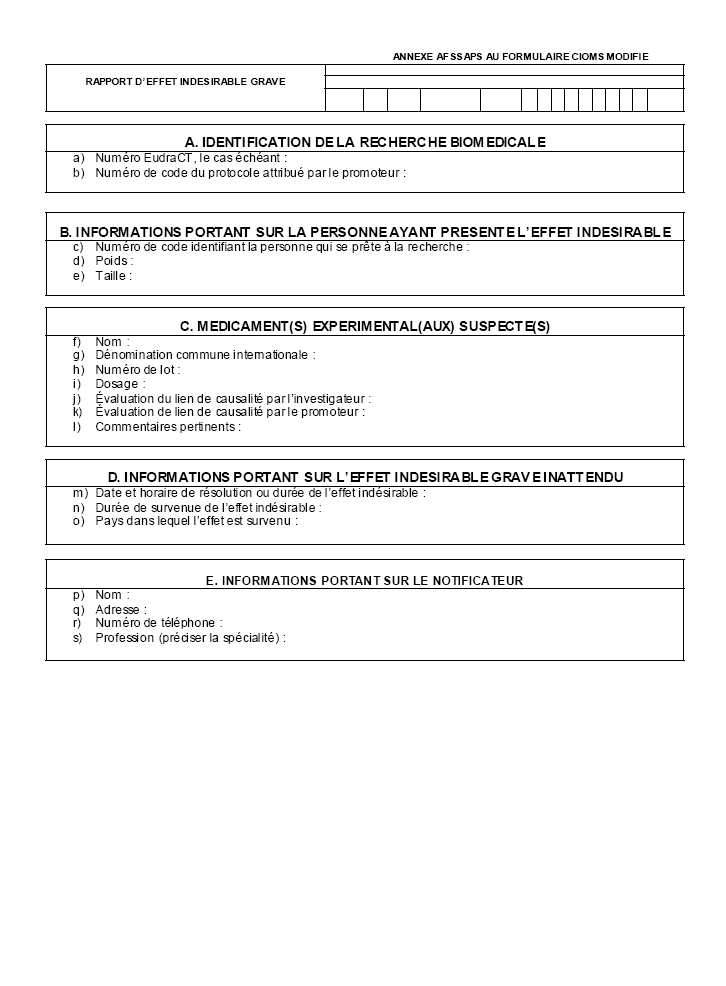


# ANNEXE ViII : assurance

#
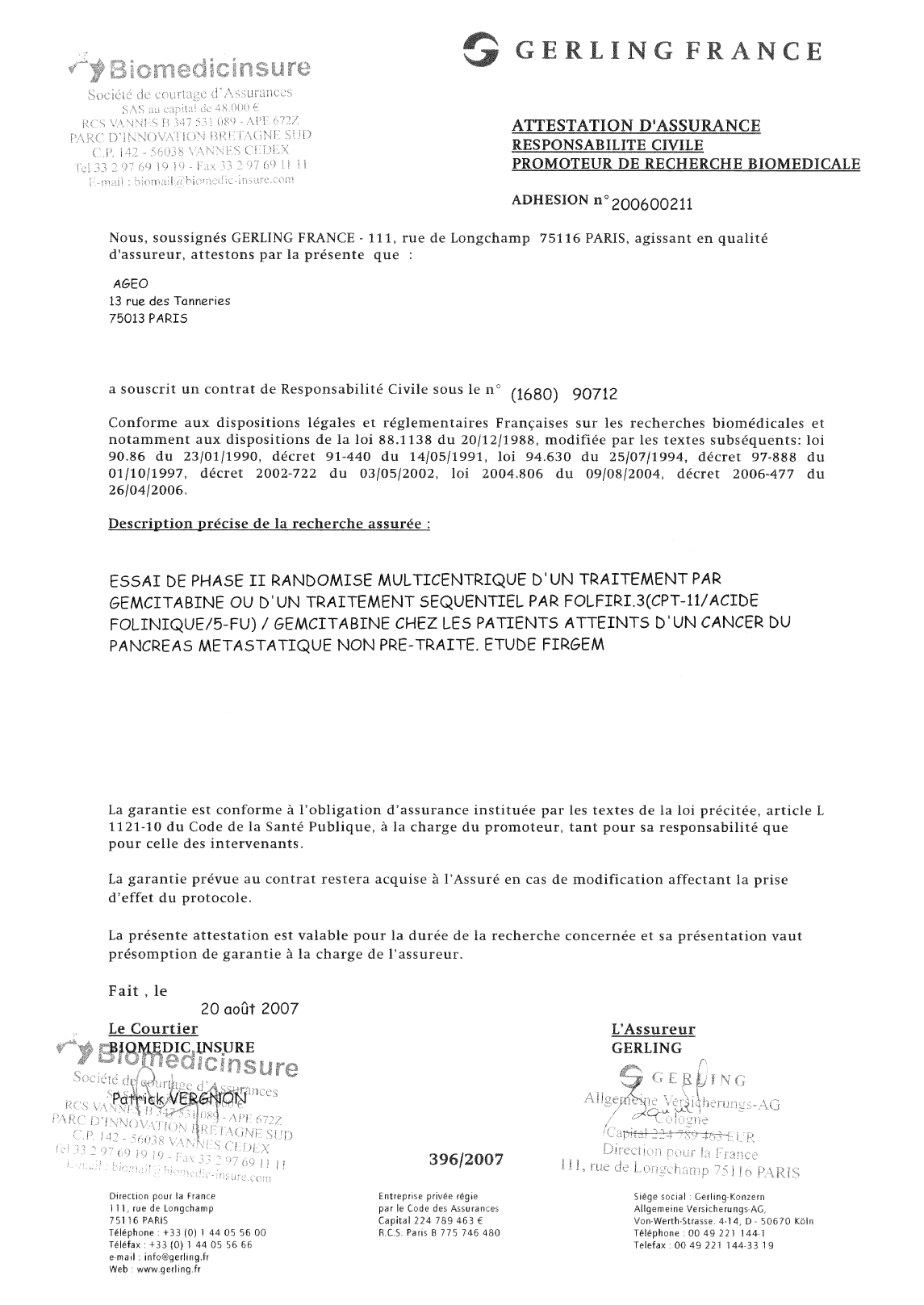


# ANNEXE IX : avis du cPP


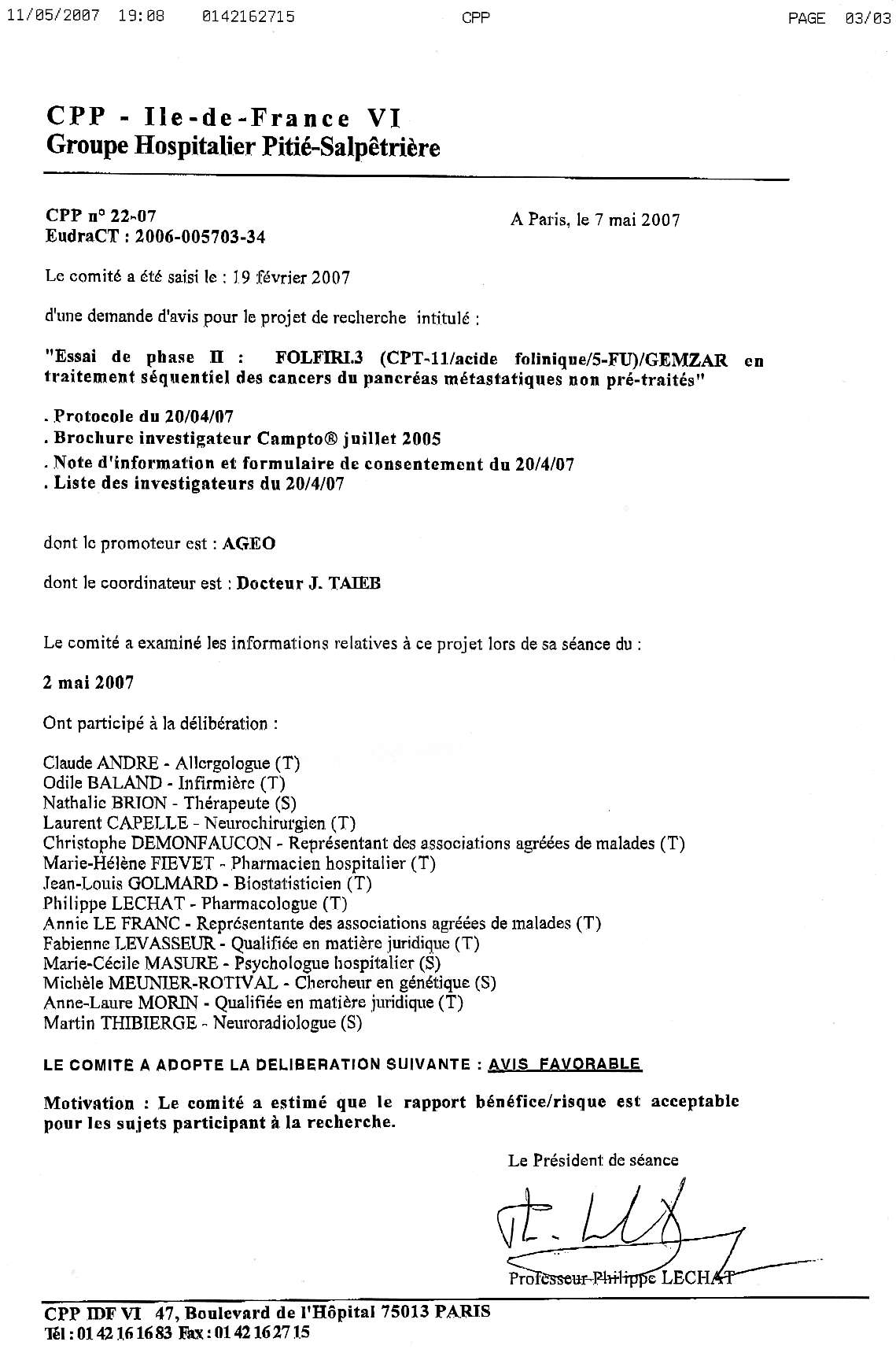


# ANNEXE X : autorisation de l'AFSSAPS

**
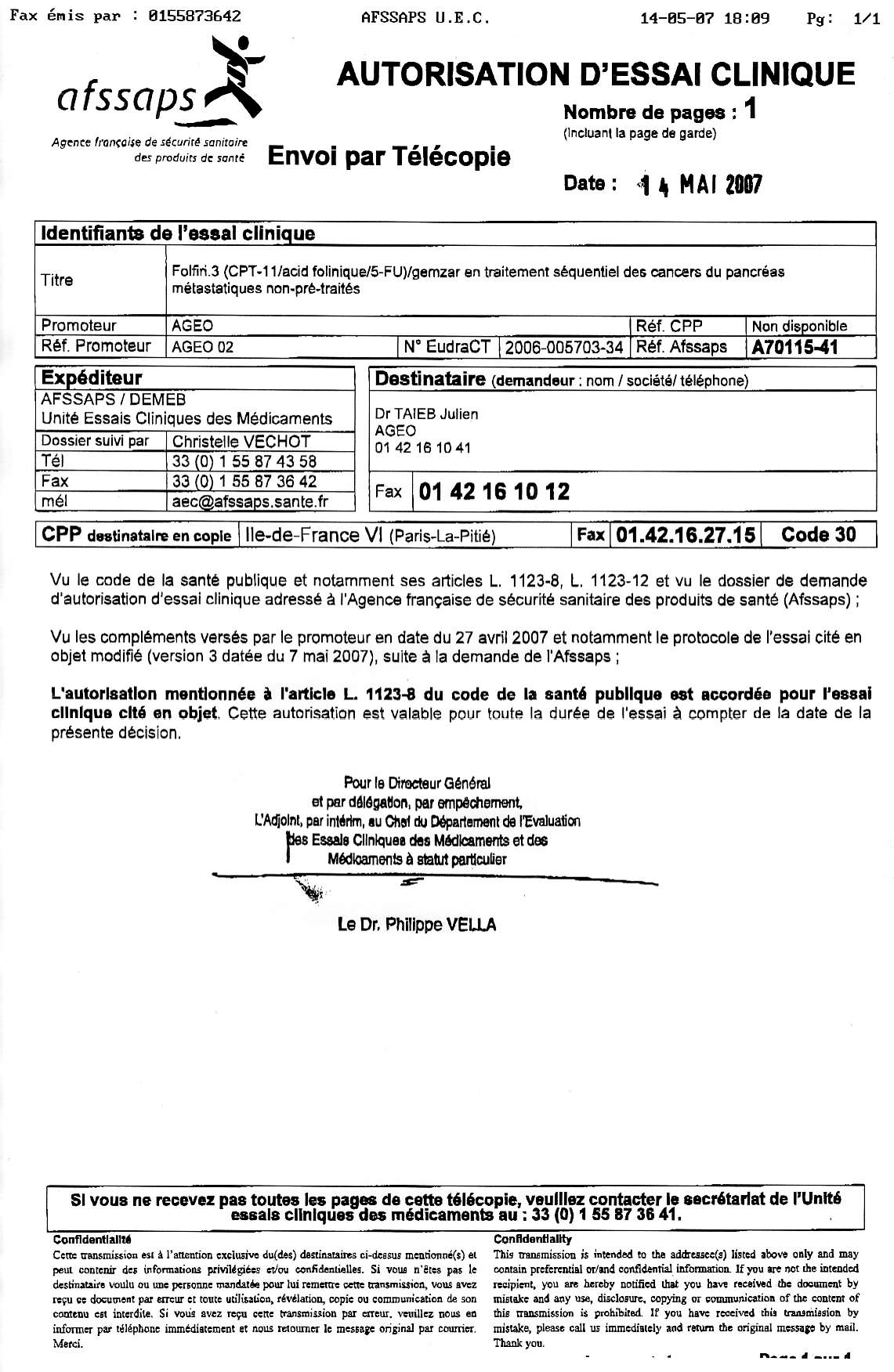
**

1. Bonnetain F et al. Longitudinal quality of life study in patients with metastatic gastric cancer. Analysis modalities and clinical applicability of QoL in randomized phase II trial in a digestive oncology. Gastroenterol Clin Biol 2005, 29(11):1113-24. [↑](#footnote-ref-2)
2. Bonnetain F et al. Longitudinal quality of life study in patients with metastatic gastric cancer. Analysis modalities and clinical applicability of QoL in randomized phase II trial in a digestive oncology. Gastroenterol Clin Biol 2005, 29(11):1113-24. [↑](#footnote-ref-3)
