# Supplementary material for: Sequential FOLFIRI.3 + Gemcitabine Improves Health-Related Quality of Life Deterioration-Free Survival of Patients with Metastatic Pancreatic Adenocarcinoma: A Randomized Phase II Trial
Source: PLoS One. 2015 May 26;10(5):e0125350. doi: 10.1371/journal.pone.0125350 (PMC4444351; doi:10.1371/journal.pone.0125350)
Supplement: S2 Protocol — (DOC) [file pone.0125350.s006.doc]

# summary of the protocol

| **Title of the trial** | A randomized multicenter phase II trial of a regimen of gemcitabine or a sequential chemotherapy FOLFIRI.3 (CPT-11 plus folinic acid plus 5-FU) followed by gemcitabine in patients with previously untreated metastatic pancreatic adenocarcinoma : the FIRGEM Study |
| --- | --- |
| **Sponsor** | AGEO |
| **Coordinating investigator** | Professeur Julien TAIEB  Hôpital Européen Georges Pompidou  20 rue Leblanc 75908 PARIS |
| **Investigation site** | Multicenter/ AGEO |
| **Endpoints** | Primary :   - 6 months progression free survival rate   Secondary:   - Objective response rate - Progression free survival - Overall survival - Survival to treatment failure (arm A) - Tolerance - Clinical benefit - Quality of life (EORTC QLQ-C30) |
| **Target population and eligibility criteria** | Inclusion criteria :   - Pathologically proven pancreatic adenocarcinoma (on primitive or metastatic lesion) - Metastatic disease - At least one measurable lesion (RECIST criteria) - 18 ≤ age ≤ 75 years - Life expectancy >12 weeks - Performance status OMS < 2 - No prior chemotherapy (adjuvant chemotherapy by gemcitabine is allowed if it was administered more than 12 months before the inclusion) - No prior radiation therapy (except if there is at least one measurable target outside irradiation area) - Pain well controlled before the inclusion - Neutrophiles ≥1500/mm^3^, platelets ≥ 100000/mm^3^, Hb ≥9g/dl - ASAT and ALAT < 5 UNL - Serum bilirubin ≤ 1,5 UNL - Normal renal function test (serum creatinine concentration ≤ 130 μmol/l or creatine clearance ≥ 60 ml/min) - The exam of reference prints (CTscan or RMN) must be made within 3 weeks before the treatment's initiation - Signed written informed consent obtained prior the inclusion     Exclusion criteria :   - Bile duct adenocarcinoma or ampullary tumors, - History of another major cancer within 5 years (except for basal cell skin cancer and carcinoma in situ of the cervix and adequately treated with curative intent), - Brain or bone metastasis from PC (no need of systematic CTscan), - Active infection, - Chronic diarrhoea or inflammatory bowel disease, - Symptomatic intestinal obstruction, - Uncontrolled hypercalcaemia, - Uncontrolled pain, - Significant history of cardiac or respiratory disease, - Pregnancy or breast-feeding. |
| **Method** | Multicentre, randomized, open phase II trial. Randomization will be done using minimization technique with the following stratification criteria :  -Center  -OMS PS 0 vs 1  -One vs >1 metastatic sites |
| **Number of subjects** | 98 patients |
| **Trial design and treatment duration** | **Patients will be randomized (1/1) to receive :**  **⏺ Arm A (experimental arm) : alternately,**  **- FOLFIRI.3 (4 courses per cycle; so ≈ 2 month)**  Day 1 : CPT11, 90 mg/m² intravenously (i.v.) for 60 minutes  Day 1 : • H_0_ - H_2_ , folinic acid dl 400 mg/m² (or L 200 mg/m²)  • H_2_ - H_48_ , continuous infusion of 5-FU at the dose of 2000 mg/m²  Day 3 : CPT11, 90 mg/m² intravenously for 60 minutes at H_48_  **This treatment will be repeated every 14 days (d1=d15)**  **Clinical, biological and morphological will be done after.**    **- GEMCITABINE (6 courses, one course three weeks out of four; so ≈ 2 month per cycle)**  Day 1: gemcitabine at the dose of 1000 mg/m² in 500 ml normal saline infusion at a fixed dose rate of 10 mg/m²/min (i.e. 100 min). This treatment is administered at D1, D8, D15 and at D29, D36, D43.  **Clinical, biological and morphological will be done after.**  **This therapeutic sequence (FOLFIRI.3 followed by GEMCITABINE) will be repeated until disease progression or unacceptable toxicity.**  **The response or the progression with each treatment will be censored. In case of progression or limiting toxicity with one of these two treatments, the other one will be continued until tumoral progression, limiting toxicity or patient’s refusal.**  **⏺ Arm B (reference arm) :**  Day 1: gemcitabine at the dose of 1000 mg/m² in 500 ml normal saline infusion at a fixed dose rate of 10 mg/m²/min (i.e. 100 min). This treatment is administered at D1, D8, D15 and at D29, D36, D43.  **Clinical, biological and morphological will be done after.**  **This treatment will be continued until tumoral progression, limiting toxicity or patient’s refusal (D1=D57).** |
| **Assessment criteria** | - Adverse events are graded according the NCI-CTG v3.0 criteria before each cycle. - Tumor response is evaluated every 2 months by a CT-scan according to the RECIST criteria. Iconographic documents used in the evaluation of the response will be reviewed by a committee of two independent radiologists. - Response and progression will be documented in each arm. Moreover the response or the progression with each treatment (FOLFIRI.3 and gemcitabine) in arm A - Progression free survival is defined in both arm as time from randomisation to first progression using the RECIST criteria or death. Progression free survival will be censored to assess study primary objective: 6 months progression free survival rate. - Survival to treatment failure will be defined for the experimental arm (A) as time from randomisation to time to progression (RECIST) with each treatment (FOLFIRI.3 and gemcitabine), or death. A sensitivity analysis will be performed on this parameter at 6 months using the fleming criteria. - Clinical benefit will be defined by a weight gain of more than 10%, an improvement in performance status (WHO) to one minimum point, and a significant reduction or a cessation of analgesics initially made ​​by the patients. - Health-related QoL was evaluated every 2 months with the EORTC QLQ-C30 questionnaire version 3.0. Time until definitive QoL deterioration will be defined by time between inclusion and a 5-point difference as compared to baseline, considered as the minimal clinically important difference (MCID) in QoL. |
| **Follow up after study treatment** | Patient will be considered as study outgoing thirty days after last administration of traitment.  Patients' survey will be done by the physician every 2 months for 2 years after the end of treatment or patient’s death. |
| **Statistical analysis** | The primary end point of this phase II study is progression free survival at 6 Months. A Fleming one step design was used to plan this trial. With the following assumptions  H0 : progression free survival at 6 months = 25% (unacceptable efficacy)  H1 : progression free survival at 6 months = 45%  With an α type I error = 5%and a β type II error = 10%, it was required to include 46 patients/arm.  At the end of the study among the 46 patients/arm:  - if we observed 15 or less than 15 patients with no progression or death (32.6%), the 6 Months progression free survival was significantly lesser than 30%. Treatment would be declared as not interesting for a phase III trial  - if we observed 16 or more than 16 patients with no progression or death (34.8%), the 6 Months progression free survival was significantly greater than 30%. Treatment would be declared interesting for a phase III trial  A total of 49 patients/arm is needed considering the risk of 5% of lost of sight patients. Finally it will be necessary to include a total of 98 patients.  The analyses will be performed on intent-to-treat principle.  Progression free survival and survival curves will be estimated using the Kaplan-Meier technique.  The safety data will be presented for all patients who start treatment, summarized according to the actual treatment received. (per protocol analyses)  The safety data of the different infusional schedules will be evaluated separately. |
| **Length of the study** | Overall length study (including the follow up period) is 30 months: November 2007–June 2010. Recruitment period is 24 months: November 2007 – December 2009. Individual length survey : 6 to 30 months. Final analysis: 2,5 years after the beginning of the study (June 2010). |
| **INSURANCE** | SM Biomedic |

**Dosage adjustment guidelines for toxicities**

| **Toxicity/grade NCI** | **1** | **2** | **3-4** |
| --- | --- | --- | --- |
| Anemia | No reduction of dose | No reduction of dose | No reduction of dose |
| Neutropenia  Thrombocytopenia | No reduction of dose | No reduction of dose *, ** | - 5-FU 1600 mg/m² (48h)  - CPT-11 80mg/m² at D1 and D3  - gemcitabine 800mg/m² |
| Diarrhea | No reduction of dose | No reduction of dose *, ** | - 5-FU Pas de réduction  - CPT-11 80mg/m² à J1 et J3 |
| Other*** | - | - | - 5-FU 1600 mg/m² (48h)  - CPT-11 80mg/m² at D1 and D3  - gemcitabine 800mg/m² |

* After recovery (toxicity de grade 0 ou 1)

** In case of non recovery after 2 weeks delay : (persistence diarrhea or neutropenia grade > 2 or thrombocytopenia grade > 1), we will realize the dose reductions as for grade 3-4.

*** except: alopecia, cholinergic syndrome, nausea / vomiting in the absence of adequate treatment

The introduction of hematopoietic growth factors: EPO and G-CSF is used in cases of hematologic toxicity and confirmed in accordance with the marketing authorization of these products.
