# Supplementary material for: Sequential FOLFIRI.3 + Gemcitabine Improves Health-Related Quality of Life Deterioration-Free Survival of Patients with Metastatic Pancreatic Adenocarcinoma: A Randomized Phase II Trial
Source: PLoS One. 2015 May 26;10(5):e0125350. doi: 10.1371/journal.pone.0125350 (PMC4444351; doi:10.1371/journal.pone.0125350)
Supplement: S1 Table — (DOC) [file pone.0125350.s007.doc]

**Table S1: health-related quality of life scores at baseline according to treatment arm**

|  | N | Mean (SD) |
| --- | --- | --- |
| Global Health Status |  |  |
| Arm gemcitabine alone | 28 | 59.2 (23.3) |
| Arm FOLFIRI + gemcitabine | 29 | 56.3 (21.7) |
| Physical Functioning |  |  |
| Arm gemcitabine alone | 28 | 75.4 (23.6) |
| Arm FOLFIRI + gemcitabine | 31 | 77.9 (22.4) |
| Emotional Functioning |  |  |
| Arm gemcitabine alone | 28 | 65.3 (23.3) |
| Arm FOLFIRI + gemcitabine | 29 | 66.3 (20.7) |
| Fatigue |  |  |
| Arm gemcitabine alone | 28 | 49.1 (28.9) |
| Arm FOLFIRI + gemcitabine | 31 | 45.6 (31.8) |
| Pain |  |  |
| Arm gemcitabine alone | 28 | 42.9 (32.2) |
| Arm FOLFIRI + gemcitabine | 31 | 36.6 (25.3) |
