# Supplementary material for: Sequential FOLFIRI.3 + Gemcitabine Improves Health-Related Quality of Life Deterioration-Free Survival of Patients with Metastatic Pancreatic Adenocarcinoma: A Randomized Phase II Trial
Source: PLoS One. 2015 May 26;10(5):e0125350. doi: 10.1371/journal.pone.0125350 (PMC4444351; doi:10.1371/journal.pone.0125350)
Supplement: S2 Table — (DOC) [file pone.0125350.s008.doc]

**Table S2**: Most Common Grade 3 or 4 Adverse Events according to treatment arm

|  | **Arm 1 gemcitabine alone**  **N=49** | **Arm 2  FOLFIRI.3 + gemcitabine**  **N=49** |
| --- | --- | --- |
|  | **N (%)** | **N (%)** |
| **All toxicities grade >2** | 32 (65.3) | 38 (77.5) |
| **Hematologic toxicity**  Neutropenia  Febrile neutropenia  Thrombocytopenia  Anaemia | 12 (24.5)  0 (0.0)  9 (18.8)  3 (6.0) | 24 (49.0)  2 (4.1)  9 (18.4)  6 (12.2) |
| **Gastrointestinal toxicity**  Nausea and vomiting  Diarrhoea | 2 (4.1)  0 (0.0) | 4 (8.2)  6 (12.2) |
