# Supplementary material for: Sequential FOLFIRI.3 + Gemcitabine Improves Health-Related Quality of Life Deterioration-Free Survival of Patients with Metastatic Pancreatic Adenocarcinoma: A Randomized Phase II Trial
Source: PLoS One. 2015 May 26;10(5):e0125350. doi: 10.1371/journal.pone.0125350 (PMC4444351; doi:10.1371/journal.pone.0125350)
Supplement: S3 Table — (DOC) [file pone.0125350.s009.doc]

**Table S3: Results of the Kaplan-Meier estimation of the health-related quality of life deterioration-free survival for a QLQ-C30 score considering non-responders patients in deterioration since baseline and comparison between treatment arms**

| Scores | Treatment arm | *N* (events) | Median | HR [CI 95%] |
| --- | --- | --- | --- | --- |
|  |  |  | [CI 95%] |  |
| Global health status | Arm 1 ^a^ | 49 (37) | 2.20 [0.03 - 7.92] | 1 |
|  | Arm 2 ^b^ | 49 (33) | 3.09 [1.15 - 11.01] | 0.86 [0.53-1.39) |
| Physical functioning | Arm 1 | 49 (38) | 1.84 [0.03- 4.21] | 1 |
|  | Arm 2 | 49 (31) | 5.03 [2.33 - 12.48] | 0.57 [0.35 - 0.95) |
| Emotional functioning | Arm 1 | 49 (38) | 1.91 [0.03 - 4.37] | 1 |
|  | Arm 2 | 49 (34) | 1.91 [0.92 - 11.01] | 0.68 [0.42 - 1.12] |
| Fatigue | Arm 1 | 49 (37) | 2.20 [0.03 - 7.92] | 1 |
|  | Arm 2 | 49 (31) | 5.03 [1.97 - 12.06] | 0.70 [0.43 - 1.14] |
| Pain | Arm 1 | 49 (37) | 3.98 [0.03 - 8.25] | 1 |
|  | Arm 2 | 49 (30) | 9.46 [3.81 - 12.48] | 0.63 [0.38 - 1.04] |

^a^ Arm 1: gemcitabine alone;

^b^ Arm 2: gemcitabine+FOLRIRI.3
