# Supplementary material for: Sequential FOLFIRI.3 + Gemcitabine Improves Health-Related Quality of Life Deterioration-Free Survival of Patients with Metastatic Pancreatic Adenocarcinoma: A Randomized Phase II Trial
Source: PLoS One. 2015 May 26;10(5):e0125350. doi: 10.1371/journal.pone.0125350 (PMC4444351; doi:10.1371/journal.pone.0125350)
Supplement: S4 Table — (DOC) [file pone.0125350.s010.doc]

**Table S4. Results of the multivariate Cox regression analysis for the QFS analysis of each targeted score of the QLQ-C30 considering non-responders patients in deterioration since baseline**

|  |  | ***N* (events)** | **HR [CI 95%]** |
| --- | --- | --- | --- |
| **Global health status** |  | 98 (70) |  |
| arm ^a^ | (arm 2) vs.(arm 1) |  | 0.93 [0.52 - 1.67] |
| number of metastatic sites | (2 or more) vs. 1 |  | 2.14 [1.03 - 4.44] |
| Interaction between arm and number of metastatic sites |  |  | 0.63 [0.22 - 1.77] |
| **Physical functioning** |  | 98 (69) |  |
| arm ^a^ | (arm 2) vs.(arm 1) |  | 0.54 [0.29 - 1.01] |
| number of metastatic sites | (2 or more) vs. 1 |  | 1.79 [0.87 - 3.68] |
| Interaction between arm and number of metastatic sites |  |  | 0.96 [0.34 - 2.71] |
| **Emotional functioning** |  | 98 (72) |  |
| arm ^a^ | (arm 2) vs.(arm 1) |  | 0.65 [0.36 - 1.19] |
| number of metastatic sites | (2 or more) vs. 1 |  | 1.80 [0.88 - 3.71] |
| Interaction between arm and number of metastatic sites |  |  | 0.96 [0.34 - 2.69] |
| **Fatigue** |  | 98 (68) |  |
| arm ^a^ | (arm 2) vs.(arm 1) |  | 0.69 [0.38 - 1.25] |
| number of metastatic sites | (2 or more) vs. 1 |  | 1.87 [0.88 - 3.97] |
| Interaction between arm and number of metastatic sites |  |  | 0.81 [0.28 - 2.33] |
| **Pain** |  | 98 (67) |  |
| arm ^a^ | (arm 2) vs.(arm 1) |  | 0.63 [0.34 - 1.17] |
| number of metastatic sites | (2 or more) vs. 1 |  | 1.88 [0.91 - 3.88] |
| Interaction between arm and number of metastatic sites |  |  | 0.81 [0.28 - 2.35] |

^a^ Arm 1: gemcitabine alone, Arm 2: gemcitabine + FOLFIRI.3
